# Oral and Topical Vitamin D, Sunshine, and UVB Phototherapy Safely Control Psoriasis in Patients with Normal Pretreatment Serum 25-Hydroxyvitamin D Concentrations: A Literature Review and Discussion of Health Implications

**DOI:** 10.3390/nu13051511

**Published:** 2021-04-29

**Authors:** Patrick J. McCullough, William P. McCullough, Douglas Lehrer, Jeffrey B. Travers, Steven J. Repas

**Affiliations:** 1 Medical Services Department, Summit Behavioral Healthcare, Ohio Department of Mental Health and Addiction Services, 1101 Summit Rd, Cincinnati, OH 45237, USA; 2 Department of Psychiatry, Wright State University Boonshoft School of Medicine, 3640 Colonel Glenn Hwy, Dayton, OH 45435, USA; Douglas.lehrer@wright.edu; 3 Rose-Hulman Institute of Technology, 5500 Wabash Ave, Terre Haute, IN 47803, USA; mcculloughw1999@gmail.com; 4 Department of Pharmacology & Toxicology, Wright State University Boonshoft School of Medicine, 3640 Colonel Glenn Hwy, Dayton, OH 45435, USA; jeffrey.travers@wright.edu; 5 Wright State University Boonshoft School of Medicine, 3640 Colonel Glenn Hwy, Dayton, OH 45435, USA; repas.3@wright.edu

**Keywords:** vitamin D_3_, D_2_, calcitriol, oral, topical, serum 25-hydroxyvitamin D, psoriasis, skin diseases, UVB, phototherapy, sunshine, COVID-19, regulatory T lymphocytes

## Abstract

Vitamin D, sunshine and UVB phototherapy were first reported in the early 1900s to control psoriasis, cure rickets and cure tuberculosis (TB). Vitamin D also controlled asthma and rheumatoid arthritis with intakes ranging from 60,000 to 600,000 International Units (IU)/day. In the 1980s, interest in treating psoriasis with vitamin D rekindled. Since 1985 four different oral forms of vitamin D (D_2_, D_3_, 1-hydroxyvitaminD_3_ (1(OH)D_3_) and 1,25-dihydroxyvitaminD_3_ (calcitriol)) and several topical formulations have been reported safe and effective treatments for psoriasis—as has UVB phototherapy and sunshine. In this review we show that many pre-treatment serum 25(OH)D concentrations fall within the current range of normal, while many post-treatment concentrations fall outside the upper limit of this normal (100 ng/mL). Yet, psoriasis patients showed significant clinical improvement without complications using these treatments. Current estimates of vitamin D sufficiency appear to underestimate serum 25(OH)D concentrations required for optimal health in psoriasis patients, while concentrations associated with adverse events appear to be much higher than current estimates of safe serum 25(OH)D concentrations. Based on these observations, the therapeutic index for vitamin D needs to be reexamined in the treatment of psoriasis and other diseases strongly linked to vitamin D deficiency, including COVID-19 infections, which may also improve safely with sufficient vitamin D intake or UVB exposure.

## 1. Introduction

Psoriasis is the most common autoimmune disease in the United States, estimated to affect over 8 million people [1]. The total cost of health care in the US for psoriasis was estimated to be USD 135 billion per year in 2013 [2] and is likely much higher today. A wide variety of treatment options are currently available, classified by the National Psoriasis Foundation (NPF) as biologic drugs, bio-similar medicines, oral treatments, traditional systemic medications, UVB phototherapy and sunshine, and topically applied medications (corticosteroids, vitamin D analogues, others), and are summarized on the NPF website [3,4,5,6,7,8,9]. In addition, the American Academy of Dermatology (AAD) and the National Psoriasis Foundation released two guidelines in 2019 outlining best practices for managing this inflammatory skin disease [10,11]. One guideline extensively reviews the use of the relatively newly developed biologic agents that target specific components of the inflammatory process causing psoriasis [10], and the other focuses on the management and treatment of psoriasis with awareness of and attention to comorbidities [11]. Neither of these two references mentions the use of oral vitamin D.

While topical vitamin D is included in the list of recommended treatments by the NPF, oral vitamin D is not discussed, other than a brief comment stating, “Research on the use of vitamin D dietary supplements for treating psoriasis is limited…We recommend speaking with your health care provider about taking a vitamin D supplement” (page 11, 2018 Topical Treatments handbook) [9]. The reason for the exclusion of oral vitamin D as a recommended treatment for psoriasis by both the NPF and AAD is unclear. Four different forms of oral vitamin D have been shown to be safe treatments for psoriasis dating back to the 1930s [12,13,14,15,16,17,18,19,20,21,22,23,24,25,26,27,28], when oral vitamin D_2_ was first found to be effective in treating a number of diseases in addition to psoriasis, including asthma [29], rheumatoid arthritis [30,31], rickets [32] and tuberculosis [33,34,35,36,37,38,39]. Sunshine, UVB phototherapy and cod liver oil, a concentrated food source of vitamin D, were also noted to be effective treatments for psoriasis [12,40,41,42,43,44], rickets [45,46] and TB [39,47,48,49,50,51,52,53,54] during that era. 

There is currently much debate as to what constitutes vitamin D deficiency, insufficiency, sufficiency and toxicity, and what diseases are responsive to vitamin D supplementation. A serum 25(OH)D concentration > 20 ng/mL was defined as sufficient for the vast majority of the population by the Institute of Medicine (IOM) in 2011 [55], while serum 25(OH)D concentrations < 30 ng/mL were defined as insufficient by the Endocrine Society in 2011 [56]. A serum 25(OH)D concentration > 50 ng/mL was cited as a cause for concern by the IOM in 2011 [55], while the Endocrine Society set a serum 25(OH)D concentration of 100 ng/mL as the upper limit of normal in 2011 [56]. The risk for toxicity has been variably thought to occur with serum 25(OH)D concentrations above 150 ng/mL in 1999 [57], 240 ng/mL in 2007 [58], 300 ng/mL in 2008 [59] and 400 ng/mL in 2011 [60].

In this report we review clinical research studies that reported serum 25(OH)D concentrations in psoriasis patients who responded safely to treatment with either oral 1(OH)D_3_ [14]_,_ oral or topical calcitriol [14,19,21,22], oral vitamin D_3_ [25,26], oral vitamin D_2_ [28], UVB phototherapy [61,62,63,64] or sunshine [64]. We will summarize the serum 25(OH)D concentrations obtained before and after treatment in each report as they relate to current definitions of vitamin D deficiency, sufficiency and toxicity, as well as the safety and efficacy of the treatments. We will show that psoriasis patients commonly have normal serum 25OHD concentrations prior to treatment with vitamin D yet show significant clinical improvement after treatment, while those treated with UVB phototherapy often have serum 25(OH)D concentrations greater that 100 ng/mL post treatment without complications. 

The data reviewed in this report was never mentioned in reports published in 2011 by the IOM [55] and the Endocrine Society [56] when they issued separate recommendations for what constitutes vitamin D deficiency, insufficiency, sufficiency and toxicity. The vitamin D clinical research studies will be listed in Section 2, and the UVB phototherapy and sunshine clinical research studies listed in Section 3, along with a brief description of the design of each study. A detailed analysis of each report is provided in the Supplementary Materials Section. A summary of the key findings from these reports is presented in Section 4. A discussion of the implications of our findings in this review for the treatment of psoriasis and other diseases strongly linked to vitamin D deficiency using vitamin D, including COVID-19 infections, is included in Section 5 and Section 6. Numerous recent reports have shown a strong link between adverse outcomes from COVID-19 infections and vitamin D deficiency [65,66,67,68,69,70,71,72,73,74,75,76,77,78,79,80], with at least one pilot study showing clinical efficacy in reducing intensive care unit admissions and mortality among patients hospitalized for COVID-19 after treatment with calcitriol [80]. 

## 2. Oral Vitamin D_2_, Oral 1 Alpha-HydroxyvitaminD_3_ (1(OH)D_3_), Oral Calcitriol, Topical Calcitriol and Oral Vitamin D_3_ Safely Treat Psoriasis—1930s to 2019 

### 2.1. Sunshine and Oral Vitamin D_2_ in the 1930s—Krafka

In 1936, a report documented the use of oral vitamin D_2_ to clear psoriasis plaques in three psoriasis patients, two of which were long-standing cases [12]. 

Other reports describing variable results from the use of vitamin D in treating psoriasis were also published in that era, but unfortunately the use of oral vitamin D to treat psoriasis and other diseases soon fell out of favor due to concerns of toxicity from hypercalcemia which was observed with the supraphysiogical doses of vitamin D then used [29,30,31,33,34,35,36,37,38,39]. 

Published reports on the use of vitamin D in the treatment of psoriasis did not resume again until the 1980′s, when a serendipitous observation was made in a psoriasis patient who was being treated with oral 1-hydroxyvitamin D_3_ (1(OH)D_3_) for osteoporosis, whose skin showed remarkable clinical improvement [13]. This observation led to the resurrection of research into the use of vitamin D in treating psoriasis, which continues through today [12,13,14,15,16,17,18,19,20,21,22,23,24,25,26,27,28,81,82,83,84,85,86,87,88,89,90,91,92,93,94,95,96,97,98,99,100,101,102,103,104,105,106,107,108,109]. 

### 2.2. Oral 1 Alpha-HydroxyvitaminD_3_, Oral Calcitriol and Topical Calcitriol in the 1980s—Morimoto 

The impetus for this study was the chance observation made the year before by two of the investigators when they were using 1(OH)D_3_ as a treatment for osteoporosis in a patient who happened to have psoriasis, and whose skin cleared completely [13]. “This observation prompted us to confirm the effect by an open-design study” [14].

In the open-design study, both oral 1(OH)D_3_, and oral and topically applied calcitriol were found to be safe and effective in clearing psoriasis skin lesions [14]. A total of 40 patients were studied: (a) 17 with oral 1(OH)D_3_, (b) 4 with oral calcitriol, and (c) 19 with topical calcitriol. 

Morimoto and colleagues published the results of 3 other clinical trials in the 1980s with similar results [15,16], and a review of their experience in 1989 [17]. In their review, they concluded: “These data suggest that exogenous active forms of vitamin D_3_ are effective for the treatment of psoriasis, and that the endogenous 1,25-dihydroxyvitamin D level also may be involved in the development of this disease”.

In the 1980s several important discoveries were made regarding vitamin D and the skin leading to the realization that the skin is both the site of production of vitamin D and a target organ for its actions:

(a) Calcitriol could be synthesized in the skin
(b) Vitamin D receptors are present in the skin
(c) Vitamin D inhibited the proliferation of cultured keratinocytes and induced them to terminally differentiate [18,19,20,83,84,90].

### 2.3. Oral and Topical Calcitriol in the 1980s and 1990s—Smith, Huckins, Perez and Holick 

Reports published beginning in 1987 by Holick et al. described the safe and effective use of oral calcitriol in treating psoriasis [18,19,20,21,22,23]. One report also examined the use of topical calcitriol and found it to be safe and effective as well [19]. 

### 2.4. Oral and Topical Calcitriol 12-Month Study, 1988—Smith 

In 1988 [19] calcitriol was tested in three different ways:

(a) On cultures of fibroblasts and keratinocytes from patients with psoriasis
(b) Orally in 14 patients with moderate to severe psoriasis
(c) Topically in 3 patients with psoriasis.

The authors concluded their report stating: “Topical or oral use of 1,25-(OH)_2_D_3_ heralds a new mode of treatment that appears to be both safe and effective for the treatment of psoriasis”. And as was shown by Morimoto, many patients with active psoriasis had baseline serum 25(OH)D_3_ concentrations > 20 ng/mL and improved significantly after treatment with oral or topical vitamin D_3_. 

### 2.5. Oral Calcitriol 6-Month Study in Psoriatic Arthritis, 1990—Huckins

In 1990 ten patients with active psoriatic arthritis were treated daily with oral calcitriol for 6 months in an open label trial. The goal of the study was to determine if the treatment would be beneficial for the arthritis, and if so, was there a correlation between the improvement in the skin lesions and the improvement in the arthritis. The dose of calcitriol was titrated from 0.5 µg/day to a maximum of 2 µg/day.

“It often took 2–3 months for improvement to occur, and improvement never occurred at a dosage < 1.5 ug/day”. Five patients chose to stay on the treatment at the end of the study.

### 2.6. Oral Calcitriol 3-Year Dose Titration Safety Study, 1996—Perez 

In 1996, a three-year follow-up study of 88 patients with plaque type psoriasis involving at least 15% of their body surface who were treated with oral calcitriol was published. The doses of calcitriol used ranged from 0.5 µg/day to 4.0 µg/day. The mean calcitriol dose was 2.1 µg/day at 24 months, and 2.4 µg/day at 36 months. A total of 88% of the patients noted some degree of improvement in their disease. Complete clearing occurred in 26.5%, moderate improvement occurred in 36.2%, and slight improvement occurred in 25.3%. A total of 12% of the patients had no change in their disease severity. 

The mean baseline serum 25(OH)D_3_ concentration was 31.8 ± 14 ng/mL, indicating that many of the 88 patients had pre-treatment serum 25(OH)D_3_ concentrations > 20 ng/mL (exact number not provided). The mean serum 25(OH)D_3_ concentration was not affected by calcitriol supplementation and did not change significantly over time. The fact that serum 25(OH)D_3_ concentrations did not change was as expected, as calcitriol is the active hormone form of vitamin D and is not metabolized into serum 25(OH)D_3_. At the end of the opening summary paragraph, the authors concluded “Oral calcitriol is effective and safe for the treatment of psoriasis”.

These 5 studies [12,14,19,21,22] detail the safe and effective use of oral vitamin D_2_ (1936), oral 1(OH)D_3_ (1986), oral calcitriol (1986,1988,1990,1996), and topical calcitriol (1986,1988) in treating psoriasis. There are very few reports published in the past 30 years describing the use of oral vitamin D_2_ or oral vitamin D_3_ (the precursors to serum 25(OH)D_3_ and calcitriol) in treating psoriasis. Three relatively recent reports [25,26,28] that do describe the use of oral vitamin D_3_ (2012, 2013) and oral vitamin D_2_ (2019) in successfully controlling plaque psoriasis will be reviewed next. These reports show similar clinical benefits and safety as the previous reports showed, even though much higher post-treatment serum 25(OH)D_3_ concentrations were observed as would be expected, as both vitamin D_2_ and vitamin D_3_ are metabolized into 25(OH)D_3_ prior to forming calcitriol. 

### 2.7. Serum 25(OH)D_3_ Concentrations in 2 Patients with Plaque Psoriasis after 5 Months’ Oral Vitamin D_3_ in 2012—McCullough 

In 2012, one of the authors (PM) presented a poster describing the results of using oral vitamin D_3_ to successfully control chronic plaque psoriasis in 2 patients at the 15th Workshop on Vitamin D in Houston, Texas [25]. The patients were provided with over the counter 5000 IU vitamin D_3_ gel caps and were instructed to take 40,000 IU/day for 2 weeks, and then reduce the dose to 10,000 IU/day. 

No adverse reactions were noted, and both patients reported marked clinical improvement in their skin and in their quality of life. Both patients later experienced recurrence of the plaques within a month after stopping oral vitamin D_3_ intake, and both were able to achieve clear skin again after resuming the oral vitamin D_3_. The recurrence of psoriasis with cessation of oral vitamin D intake and clearing again with resumption of oral vitamin D intake was also previously reported by Smith et al. in 1988 [19]. 

### 2.8. Serum 25(OH)D_3_ Concentrations after 6 Months of Taking 35,000 IU/Day of Oral Vitamin D_3_ in 25 Patients with Either Plaque Psoriasis or Vitiligo, 2013—Finamor 

In 2013, results from a 6-month follow-up study using 35,000 IU/day of oral vitamin D_3_ to treat 9 patients with psoriasis and 16 patients with vitiligo were published [26]. The PASI score significantly improved in all nine patients with psoriasis. Fourteen of 16 patients with vitiligo had 25–75% repigmentation. A significant negative correlation was observed between the PASI scores and serum 25(OH)D_3_ concentrations.

In the psoriasis group, mean serum 25(OH)D_3_ concentrations increased from 14.9 ± 7.4 ng/mL at baseline to 106.3 ± 31.9 ng/mL at 6 months. In the vitiligo group, mean serum 25(OH)D_3_ concentrations increased from 18.4 ± 8.9 ng/mL at baseline to 132.5 ± 37.0 ng/mL at 6 months. The authors noted that “Laboratory or clinical signs of toxicity (hypercalcemia, hypercalciuria or kidney dysfunction) were not observed in any of the 25 participants, including a patient with vitiligo who reached a serum concentration of 25OHD_3_ of 202 ng/mL”. 

The authors’ main conclusion was “In summary, the present study suggests that, at least for patients with autoimmune disorders like vitiligo and psoriasis, a daily dose of 35,000 IU of vitamin D_3_ is a safe and effective therapeutic approach for reducing disease activity”. The yearly cost of treatment with oral vitamin D_3_ at a dose of 35,000 IU/day is around USD 98 per year, based on currently available over the counter pricing at USD 14/bottle for a USP verified bottle of 400 gel caps with 5000 IU/cap. 

### 2.9. Serum 25(OH)D_2_ Concentrations in a Patient with Plaque Psoriasis after 42 Months of Taking 50,000 IU/Day of Oral Vitamin D_2_, 2019—McCullough 

In 2019, a paper describing results from supplementing long-term hospitalized patients with 5000 IU/day to 50,000 IU/day of vitamin D_3_ for over 7 years was published by one of the authors (PM), who made it a standard of care beginning in April 2009 to offer all long-term hospitalized patients under his care supplementation with oral vitamin D_3_ in doses of either 5000 IU/day or 10,000 IU/day [28]. This was done for several reasons:

(a) patients receive very little sunshine in the hospital
(b) there is very little vitamin D in the food they eat
(c) serum 25-hydroxyvitamin D (25(OH)D) production in the skin from UVB phototherapy was first estimated in the 1970s to range from 10,000 to 25,000 IU/day [39,53,56,110,111,112,113]
(d) vitamin D, sunshine and UVB phototherapy were shown to be effective treatments for several diseases in the 1930s and 1940s, and again beginning in the 1980s as discussed earlier [12,13,14,15,16,17,18,19,20,21,22,23,24,25,26,27,28,29,30,31,32,33,34,35,36,37,38,39,45,46,47,48,49,50,51,52,53,54]. In addition, several patients received daily doses of vitamin D_2_ or vitamin D_3_ ranging from 20,000 to 50,000 IU/day based on specific disease concerns.

There have been over 6000 admissions to SBH since 2011. A recent sampling of patients not on vitamin D_3_ (*n* = 777; combination of new admissions and long-term patients who declined supplementation) showed a mean serum 25(OH)D_3_ concentration of 27.1 ng/mL (range 4.9 to 74.8 ng/mL). Patients on vitamin D_3_ long enough to develop serum 25(OH)D_3_ concentrations > 74.4 ng/mL (*n* = 418) had a mean serum 25(OH)D_3_ concentration of 118.9 ng/mL (range 74.4 to 384.8 ng/mL). The highest serum 25(OH)D_3_ concentrations observed on 10,000 IU/day was 202 ng/mL. 

The mean and range of serum calcium concentrations were almost identical in the two groups, despite the wide disparity in serum 25(OH)D_3_ concentrations. The average serum calcium concentrations were 9.5 mg/dL (no D_3_) vs. 9.6 mg/dL (D_3_), with ranges of 8.4 mg/dL to 10.7 mg/dL (no D_3_) vs. 8.6 mg/dL to 10.7 mg/dL (D_3_), after excluding patients with other causes of hypercalcemia. 

There were no adverse events observed in any patients taking 5000 to 10,000 IU/day for several years, in spite of serum 25(OH)D_3_ concentrations reaching as high as 202 ng/mL. In addition, several patients, as well one of the authors, having taken daily oral doses of vitamin D ranging from 20,000 to 60,000 IU/day for 2 to 6 years, achieved serum 25(OH)D_3_ concentrations as high as 384 ng/mL without any complications [28,114].

In our 2019 report we included a case report of a patient admitted with poorly controlled plaque psoriasis whose skin improved dramatically within a few months of starting 50,000 IU/day of oral vitamin D_2_ and has remained clear for many months. His serum calcium and iPTH concentrations have been checked numerous times and have remained normal. No adverse events related to vitamin D supplementation have been observed. His quality of life has improved significantly. The yearly cost for the vitamin D_2_ used was USD 36.50, as we are able to obtain 50,000 IU capsules of vitamin D_2_, with 100 capsules/bottle for USD 10 a bottle. 

Table 1 shows the study year, type of vitamin D, route, dose used, number of patients treated, study duration and number of patients showing significant clinical improvement following treatment in the clinical trials and case reports reviewed.

As shown in the table, all four forms of oral vitamin D and topical calcitriol were effective in treating psoriasis. No serious safety concerns were reported in any of the clinical trials or case reports. See the Supplementary Materials for more information on safety monitoring in each clinical trial and case report. This will also be reviewed in more detail in Section 4. 

Table 2 summarizes the baseline mean serum 25(OH)D concentrations, range at baseline if indicated, number (*N*) greater than 20 ng/mL at baseline, number (*N*) greater than 50 ng/mL at baseline, and peak serum 25(OH)D concentrations post-treatment in the oral vitamin D clinical trials and case reports reviewed. 

As seen in the table, numerous pre-treatment serum 25(OH)D concentrations were above 20 ng/mL, ranging as high as high as 67 ng/mL (where data was provided), yet the patients psoriasis improved following treatment with the four forms of vitamin D used. No significant change over time was noted in serum 25(OH)D concentrations in patients treated with 1(OH)D and calcitriol, as neither compound is metabolized into 25(OH)D. 

In contrast, significant increases in serum 25(OH)D concentrations were noted in patients following daily treatment with 35,000 units of vitamin D_3_ for 6 months and 50,000 units of vitamin D_2_, for over 2 years, with peak serum 25(OH)D concentrations of 202 ng/mL and 308 ng/mL reported without complication. 

In the next section we will review four clinical phototherapy trials in patients with plaque psoriasis which show results very similar to and consistent with those just reviewed in the nine oral vitamin D plaque psoriasis clinical trials and case reports. 

## 3. Changes in Serum 25(OH)D_3_ Concentrations in Psoriasis Patients Treated with UVB Phototherapy and Sunshine—1996, 2009, and 2010 

The use of phototherapy to treat disease dates back to the 1890s when Finsen developed a method to cure TB with refracted light rays from an electric arc lamp [28,39,40,41,42,43,44,48,49,50,52,54]. Several recent reviews give an excellent overview of the evolution of the use of phototherapy for treating human disease, including psoriasis [40,41,42,43,44]. The first documented use of UVB phototherapy in treating psoriasis dates back to Gockerman in the 1920s [41,42,43]. UVB phototherapy is now a well-established, relatively safe and cost-effective option for treating psoriasis [40,41,42,43,44,61,62,63,64,115,116,117,118,119,120,121,122,123,124,125,126,127,128,129,130,131].

In this section, we will briefly review four UVB phototherapy psoriasis treatment studies that provided baseline and post-treatment serum 25(OH)D_3_ concentrations [61,62,63,64]. A more detailed review of each study is provided in the Supplementary Materials. 

Significant increases in 25(OH)D_3_ from baseline were noted in the UVB phototherapy studies, with several patients obtaining serum 25(OH)D_3_ concentrations > 100 ng/mL without any adverse effects while observing significant improvement in their skin. As noted in the oral vitamin D studies, baseline serum 25(OH)D_3_ concentrations > 20 ng/mL were also commonly observed in these reports and increased after treatment. One study also included a group of patients treated with sunshine, in which the observed changes in serum 25(OH)D_3_ concentrations were significantly lower than those after treatment with UVB therapy [64]. 

### 3.1. Serum 25(OH)D_3_ Concentrations in Psoriasis Patients after 8 Weeks of UVB Phototherapy, 1996—Prystowsky 

In 1996 changes in serum 25(OH)D_3_ concentrations were assessed in 15 patients with plaque-type psoriasis treated with UVB phototherapy [61]. Seven of these patients were treated with oral calcitriol (0.5 to 2 ug/day), and eight with placebo. Serum concentrations of 25(OH)D_3_ and calcitriol were measured before, during and after treatment in 13 patients. Serum chemistry and hematology laboratory evaluations were also done. 

All patients treated with phototherapy showed significant increases in their serum 25(OH)D_3_ concentrations. Significant improvement was noted in disease severity in all patients in both groups, with no significant difference between groups.

Post-treatment 10 of 13 (77%) had a serum 25(OH)D_3_ concentrations > 50 ng/mL, and 3 patients (23%) had serum 25(OH)D_3_ concentrations > 100 ng/mL, two in the placebo group and one in the calcitriol group. Their serum 25(OH)D_3_ concentrations ranged from 123 ng/mL to 159 ng/mL. 

The authors noted that “because phototherapy for psoriatic plaques produces changes in keratinocytes similar to those described for 1,25-(OH)2D3 (i.e., slowed proliferation and enhanced differentiation), this raises the possibility that one of the mechanisms of action of UVB may be through enhanced vitamin D metabolism”.

### 3.2. Serum 25(OH)D_3_ Concentrations in Psoriasis Patients after 1–4 Months of NB-UVB Phototherapy, 2010—Ryan 

In a 2010 report, serum 25(OH)D_3_, ionized calcium, intact parathyroid hormone (iPTH) and alkaline phosphatase concentrations were assessed in 30 patients with plaque psoriasis before and after treatment with narrowband (NB) UVB phototherapy [62]. Comparison was made to a matched untreated control group of 30 patients with plaque psoriasis. Patients in the treatment group received NB-UVB phototherapy 2 to 3 times a week. Treatment continued until essentially complete clearing of the psoriasis occurred, which took between 25 to 118 days (median 51 days). 

In the NB-UVB group baseline serum 25(OH)D_3_ concentrations ranged from 9 ng/mL to 46 ng/mL (median 23 ng/mL). Post-NB-UVB phototherapy, after complete skin clearing, the range of serum 25(OH)D_3_ concentrations increased to 32 to 112 ng/mL (median 59 ng/mL).

### 3.3. Serum 25(OH)D_3_ Concentrations in Psoriasis Patients after 8–12 Weeks of NB-UVB and BB-UVB Phototherapy, 2009—Osmancevic 

In a 2009 report, serum 25(OH)D_3_, calcitriol, iPTH, calcium and creatinine concentrations were measured in 68 patients with plaque psoriasis before and after treatment with either broadband UVB (BB-UVB, *n* = 26) or NB-UVB (*n* = 42) phototherapy [63]. All patients were treated with whole body exposure for 8 to 12 weeks, with the doses of UVB adjusted based on the skin phenotype and the erythemal response noted during treatment. 

The purpose of the study was to determine if there was a difference in vitamin D production with NB-UVB versus BB-UVB phototherapy. The use of oral or topical vitamin D, vitamin D analogues, or any biologics was prohibited. Patients were treated either in the spring (*n* = 39) or in the winter (*n* = 29). 

There was no significant difference in the total number of treatments needed, but the treatment time was four times longer in the NB-UVB group compared to the BB-UVB group. Psoriasis plaques improved in all patients in both groups. 

Serum 25(OH)D_3_ concentrations increased in both groups, with a more pronounced increase noted in the BB-UVB versus NB-UVB group. In the BB-UVB group, the baseline mean serum 25(OH)D_3_ concentration was 37.9 ± 16.9 ng/mL and increased to 69.4 ± 19.7 ng/mL after treatment. In the NB-UVB group, the baseline mean serum 25(OH)D_3_ concentration was 34.8 ± 11.9 ng/mL and increased to 55.3 ± 17.6 ng/mL after phototherapy. 

A line plot of individual serum 25(OH)D_3_ concentrations before and after treatment for the two groups showed that at least 3 patients in the BB-UVB group had serum 25(OH)D_3_ concentrations > 100 ng/mL post-treatment, but the actual values were not indicated. 

### 3.4. Serum 25(OH)D_3_ Concentrations in Psoriasis Patients after 15 Days of Sunshine or 8–12 Weeks of NB-UVB or BB-UVB Phototherapy, 2010—Osmancevic 

In a 2010 report, serum 25(OH)D_3,_ calcitriol, PTH, calcium and creatinine concentrations in psoriasis patients were measured before and after treatment with sunshine, NB-UVB and BB-UVB phototherapy [64]. This report was a discussion of data aggregated from 3 studies, including data in the previously discussed report [63]. The two additional studies included a group of 24 post-menopausal women with psoriasis who were treated with whole body BB-UVB phototherapy 2 to 3 times a week for 8 to 12 weeks, and a group 20 psoriasis patients who were treated with whole body heliotherapy (sunshine) daily for 2 weeks. The authors stated that they had 2 main aims:

(a) To increase the knowledge about the effects of phototherapy on vitamin D production during the treatment of psoriasis,
(b) To see if there were differences between the effect of BB-UVB, NB-UVB and heliotherapy on vitamin D synthesis in psoriasis patients.

A similar efficacy was reported with each treatment. An improvement in the PASI score of about 75% was observed in each group. However, the group treated with sunshine required only two weeks to achieve the same clinical improvement as seen after 2 to 3 months of UVB phototherapy. 

Serum 25(OH)D_3_ concentrations increased in each group. The range of serum 25(OH)D_3_ concentrations after treatment with sunshine was much lower than in the UVB groups. This may be due to the shorter duration of treatment, as well as the fact that the patients used sunscreen on areas of their body susceptible to sunburn.

Table 3 shows the study year, type of phototherapy used, the number of patients treated, study duration, and number of patients showing significant improvement following treatment in each clinical trial reviewed.

As shown in the table, all three forms of phototherapy were effective in treating plaque psoriasis. No serious safety concerns were reported in any of the clinical trials. See the Supplementary Materials for more information on safety monitoring in each clinical trial. This will also be reviewed in more detail in Section 4.

Table 4 summarizes the baseline and post-treatment mean serum 25(OH)D concentrations, and baseline and post-treatment range at of serum 25(OH)D concentrations in the phototherapy clinical studies reviewed. 

As seen in the table, numerous pre-treatment serum 25(OH)D concentrations were above 20 ng/mL, ranging to as high as 88 ng/mL, yet the patients’ psoriasis improved following treatment with the three forms of phototherapy used. This is consistent with the data reported in the oral vitamin D clinical trials and case reports reviewed in Section 2. The total number of baseline serum 25(OH)D concentrations greater than 20 ng/mL and 50 ng/mL in each clinical trial are reported in Table 5. 

Significant increases in serum 25(OH)D concentrations post-treatment were also reported in each clinical trial, with many greater than 50 ng/mL, and several greater than 100 ng/mL. Peak serum 25(OH)D concentrations of 90, 98, 112, 118, 123, and 159 ng/mL were reported following treatment with UVB phototherapy, with a peak of 60 ng/mL reported following treatment with sunshine. This data is also shown in Table 5. The mean and range of serum 25(OH)D concentrations appear to be lower pre and post-treatment in patients treated with sunshine compared to those treated with UVB phototherapy. Reasons for this are discussed in the Supplementary Materials.

Table 5 shows the distribution of serum 25(OH)D concentrations greater than 20 ng/mL, 50 ng/mL and 100 ng/mL at baseline and post-treatment in each phototherapy clinical trial. 

As seen in the table, 87% (134/154) of treated patients with active plaque psoriasis had pre-treatment serum 25(OH)D concentrations greater than 20 ng/mL, 10 of whom were also greater than 50 ng/mL. Following improvement of their psoriasis after treatment with UVB phototherapy or sunshine, 100% had serum 25(OH)D concentrations greater than 20 ng/mL, 61% (94/154) were greater than 50 ng/mL, with at least 6 greater than 100 ng/mL. 

## 4. Summary and Discussion of Key Findings in the Reviewed Reports

The following summarizes the main points highlighted in this paper thus far:

(1) Four different oral forms of vitamin D are safe and effective treatments for plaque psoriasis
(2) Normal serum 25(OH)D concentrations (>20 ng/mL) were common pretreatment but insufficient to improve psoriatic lesions
(3) High serum 25(OH)D concentrations (>100 ng/mL) were often reported with safe control of psoriasis
(4) Changes in serum 25(OH)D concentrations after treatment vary significantly with the treatment used
(5) A therapeutic dose response of psoriasis to vitamin D appears to be present
(6) Calcitriol formation is the common endpoint after treatment with vitamin D, sunshine and UVB phototherapy
(7) Psoriasis can recur with cessation of treatment with vitamin D, sunshine or UVB phototherapy
(8) Psoriasis can improve again with resumption of treatment with vitamin D, sunshine or UVB phototherapy
(9) Post treatment serum 25(OH)D concentrations are higher after UVB phototherapy compared to sunshine
(10) A paucity of adverse reactions was observed with vitamin D supplementation in the reviewed studies
(11) Clinical efficacy and safety of oral and topical vitamin D treatments are comparable to UVB phototherapy and sunshine treatments
(12) All authors reviewed stated unequivocal support for the safety and efficacy of vitamin D in treating psoriasis
(13) Estimates of vitamin D production in the 1970s are significantly lower than doses used clinically in treating diseases in the 1930s and 1940s— but significantly higher than the doses recommended for use today.

### 4.1. Four Different Oral Forms of Vitamin D Are Safe and Effective Treatments for Plaque Psoriasis

Since 1985 four different oral forms of vitamin D, specifically vitamin D_2_, vitamin D_3_, 1-hydroxyvitaminD_3_ (1(OH)D_3_), and 1,25-dihydroxyvitaminD_3_ (calcitriol) and several topical formulations of vitamin D including calcitriol [13,14,15,16,17,18,19,20,21,22,23,24,25,26,27,28,81,82,83,84,85,86,87,88,89,90,91,92,93,94,95,96,97,98,99,100,101,102,103,104,105,106,107,108,109] have been reported safe and effective treatments for psoriasis—as has UVB phototherapy and sunshine [40,41,42,43,44,61,62,63,64,115,116,117,118,119,120,121,122,123,124,125,126,127,128,129,130,131]. This is consistent with findings first reported by Krafka in 1936 [12]. 

### 4.2. Normal Serum 25(OH)D Concentrations (>20 ng/mL) Are Often Insufficient for Disease Control in Psoriasis Patients

The serum 25(OH)D concentrations reported in 10 of the 12 clinical trials reviewed (no data in 2 reports) consistently show a high percentage of pre-treatment serum 25(OH)D concentrations > 20 ng/mL, ranging up to 88 ng/mL, in patients with active plaque psoriasis [14,19,22,25,26,28,61,62,63,64]. These pre-treatment concentrations are within what is currently considered the normal range of serum 25(OH)D concentrations as defined by the IOM (20 to 50 ng/mL) [55] and Endocrine Society (30 to 100 ng/mL) [56] in 2011. However, the significant clinical improvement observed in many of these patients after treatment with the four different forms of oral vitamin D is evidence that the range of serum 25(OH)D concentrations currently classified as normal are not sufficient for disease control in patients suffering from active plaque psoriasis. 

### 4.3. High Serum 25(OH)D Concentrations (>100 ng/mL) Were often Reported with Safe Control of Psoriasis 

Post-treatment serum 25(OH)D concentrations > 100 ng/mL were observed in plaque psoriasis patients treated with UVB phototherapy [61,62,63], oral vitamin D_2_ at a dose of 50,000 IU/day for over 2 years [28], and oral vitamin D_3_ at a dose of 35,000 IU/day for 6 months [26], while none were observed in patients treated with sunshine [64], oral or topical calcitriol [14,19,22], or oral 1(OH)D_3_ [14]. Serum 25(OH)D concentrations > 100 ng/mL are currently considered to be above the normal range of serum 25(OH)D concentrations by the Endocrine Society [56], while serum 25(OH)D concentrations > 50 ng/mL are considered high by the IOM [55]. However, no significant clinical toxicity was observed in any plaque psoriasis patient who achieved these serum 25(OH)D concentrations, as indicated by the significant clinical improvement observed in their skin after each treatment without the observation of any adverse events. 

### 4.4. Changes in Serum 25(OH)D Concentrations after Treatment Vary Significantly with the Treatment Used

There were no changes in serum 25(OH)D concentrations in patients treated with either oral 1(OH)D_3_ [14], oral calcitriol [14,19,22], or topical calcitriol [14,19], as neither 1(OH)D_3_ or calcitriol is metabolized into 25(OH)D_3_. There were significant increases in serum 25(OH)D concentrations in patients treated with oral vitamin D_2,_ [28], oral vitamin D_3_, [26], UVB phototherapy [61,62,63,64] and sunshine [64], as described in Section 4.3. 

The highest serum 25(OH)D concentration observed after 6 months of 35,000 IU/day of vitamin D_3_ was 202 ng/mL [26]. The highest serum 25(OH)D concentration observed after more than 2 years on 50,000 IU/day of vitamin D_2_ was 308 ng/mL [28]. The highest post-treatment serum 25(OH)D concentration observed after 8 weeks of UVB phototherapy was 159 ng/mL reported in 1996 [61]. Current definitions of normal serum vitamin D concentrations need to be reconsidered in light of this data. 

### 4.5. A Therapeutic Dose Response to Vitamin D Appears to Be Present 

A dose response was noted by Huckins et al. in 1990 in the treatment of psoriatic arthritis with calcitriol in doses ranging from 0.5 µg to 2 µg/day, as improvement reportedly never occurred at a dosage < 1.5 ug/day [21]. Similarly, when calcitriol was titrated from 0.5 to 4 µg/day in a 3 year study of plaque psoriasis by Perez et al. in 1996, mean calcitriol doses of 2.1 µg/day at 24 months, and 2.4 µg/day at 36 months were obtained, suggesting a dose response was also observed, although this was not stated by the authors [22]. This needs further clarification for each of the four forms of oral vitamin D shown to be effective treatments for psoriasis in this review. 

### 4.6. Calcitirol Formation Is the Common Endpoint after Treatment with Vitamin D, UVB Phototherapy and Sunshine

**Figure 1:**
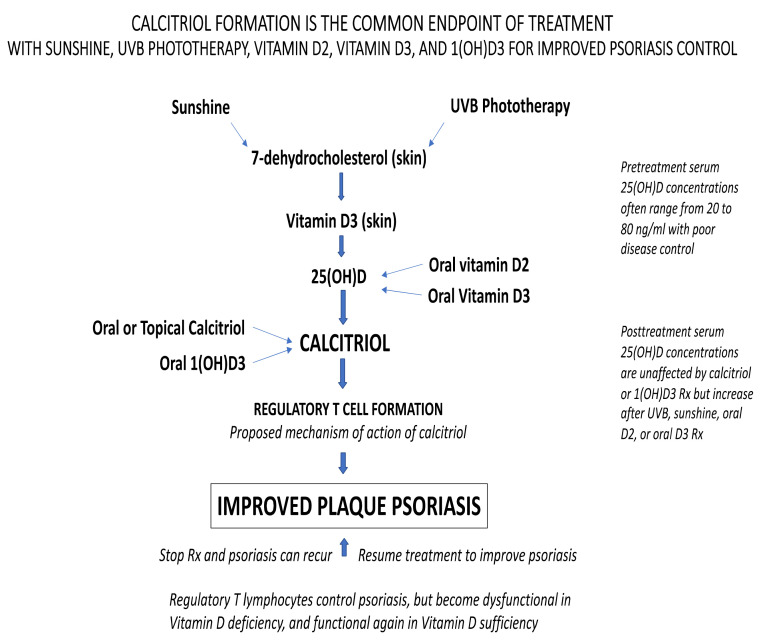
The metabolic pathway of calcitriol production from 7-dehydrocholesterol in the skin after interaction with UVB radiation present in sunshine or UVB phototherapy.

Vitamin D_3,_ vitamin D_2_, and 1(OH)D_3_ are all metabolized into calcitriol, the active hormone form of vitamin D. Sunshine and UVB phototherapy cause the formation of vitamin D_3_ in the skin from the precursor molecule 7-dehydrocholesterol, which is then metabolized to 25(OH)D and subsequently into calcitriol. The formation of calcitriol appears to be the final common pathway for these six treatments. The primary function of calcitriol is regulation of gene transcription. Calcitriol regulates several thousand genes located in many different cells and tissues throughout the body [132,133,134,135,136,137,138], including keratinocytes [19,83,84,90,103,104,105,106,109,131] and cells of the innate and adaptive immune system [139,140,141,142,143,144,145,146,147,148,149,150,151,152,153,154,155,156,157,158,159,160,161,162,163,164,165,166,167,168,169,170,171,172,173,174,175,176,177,178,179,180,181,182,183,184,185,186,187,188,189,190,191,192,193,194,195,196,197,198,199,200,201,202,203,204,205,206,207]. See Figure 1 for an illustration of this process.

### 4.7. Psoriasis Can Recur with Cessation of Vitamin D or UVB Phototherapy

Psoriasis was observed to recur with cessation of oral calcitriol by Smith et al. in 1988 [19] and with cessation oral vitamin D_3_ by McCullough et al. in 2012 [25]. Krafka noted in 1936 that psoriasis control improved with sunshine and worsened in the abcense of sunshine which was the motivation to use vitamin in the treatment of psoriasis [12]. The National Psoriasis Foundation and the American Academy of Dermatology have also reported that psoriasis will often recur after cessation of UVB phototherapy [8,119]. 

### 4.8. Psoriasis Can Improve Again with Resumption of Treatment with Vitamin D or UVB Phototherapy

Psoriasis was also noted to improve again after resuming oral calcitriol by Smith et al. in 1988 [19] and oral vitamin D_3_ by McCullough et al. in 2012 [25]. Similarly, maintenance phototherapy is recommended by the National Psoriasis Foundation and the American Academy of Dermatology due to psoriasis plaques commonly reoccurring after cessation of treatment with UVB phototherapy [8,119]. 

The exact gene products regulated by calcitriol that cause the improvement in plaque psoriasis are currently unknown but may be related to the positive effect that topical vitamin D [153,154,155,156,157,158], oral vitamin D [159,160,161,162,163,164,165,166,167,168,169,170,171,172,173,174,175,176,177,178,179,180,181,182,183,184,185,186,187,188,189,190,191,192,193,194,195,196,197,198,199,200,201,202,203,204,205,206,207], sunshine [208,209,210] and UVB phototherapy [211,212,213,214,215,216,217,218,219,220,221,222,223,224,225,226,227,228,229,230,231,232,233] have been shown to have on the formation and functional status of regulatory T lymphocytes. Tregs have been shown to play an important role in suppression of autoimmune diseases [234,235,236,237,238,239,240,241,242,243,244,245,246,247,248,249,250,251,252,253,254]. Several mechanisms of action by which Tregs control psoriasis were proposed in 2013 [246].

Thus, psoriasis appears to be controlled but not cured by vitamin D and UVB phototherapy and requires maintenance therapy to maintain disease control. This is consistent with the observed dependency of regulatory T lymphocytes on vitamin D, sunshine and UVB phototherapy to maintain their functional status. Psoriasis appears to behave like an autosomal recessive disease that becomes dominant in a state of vitamin D deficiency when Tregs are dysfunctional, and recessive in a state of vitamin D sufficiency when Treg functional status is restored. 

### 4.9. Post Treatment Serum 25(OH)D Concentrations Are Higher after UVB Phototherapy Compared to Sunshine

Treatment with UVB phototherapy consistently resulted in several patients achieving serum 25(OH)D concentrations > 100 ng/mL in the reports reviewed [61,62,63]. Treatment with sunshine resulted in smaller increases in serum 25(OH)D concentrations than observed with UVB or NB phototherapy, with none > 100 ng/mL, although several were > 50 ng/mL post treatment [64]. These results are consistent with reports of serum 25(OH)D concentrations observed in healthy individuals after prolonged sun exposure, where serum 25(OH)D concentrations > 100 ng/mL were also not observed, but serum concentrations > 50 ng/mL were commonly observed [255,256,257]. Three reports will be discussed. 

In 1971 serum 25(OH)D concentrations in eight lifeguards measured four weeks after working at an outdoor pool were included in a report by Haddad and Chyu describing the first successful assay for measuring serum 25(OH)D concentrations [255]. The mean serum 25(OH)D concentration after four weeks of sun exposure was 64.4 ± 8.7 ng/mL, and all eight were >50 ng/mL (range 53 to 79 ng/mL). 

In 2007, ninety-three individuals living in Hawaii with variable daily sun exposure ranging from total body exposure in surfers to only head, arms and hands in skateboarders, had serum 25(OH)D concentrations ranging from 11 to 71 ng/mL, of which seven (7.5%) were >50ng/mL [256]. 

In 2012, sixty traditionally living healthy dark-skinned individuals in East Africa were evaluated for serum 25(OH)D concentrations [257]. The mean serum 25(OH)D concentration was 46 ng/mL (range 23.2 to 68.4 ng/mL), with 13.3% between 20 to 32 ng/mL, 15% between 32.4 to 40 ng/mL, 28.3% between 40.4 to 48 ng/mL, 33.3% between 48.4 to 60 ng/mL, and 10% > 60.4 ng/mL, for a total of approximately 43% with serum concentrations > 50 ng/mL. 

### 4.10. A Paucity of Adverse Reactions Was Observed in the Reviewed Studies

Very few adverse events were reported in the patients treated with vitamin D, sunshine or UVB phototherapy reviewed in this report. No cases of hypercalcemia, nephrolithiasis, or renal dysfunction were reported in any of the vitamin D studies reviewed. Six total cases of hypercalciuria were reported in three reports, four with calcitriol [19,21] and two with UVB phototherapy [61]. No cases of hypercalciuria were reported after 6 months of treatment in 25 patients with 35,000 IU/day of vitamin D_3_ [26], and a very mild elevation was noted in an individual after 2 years of treatment with 50,000 IU/day of vitamin D_2_ [28].

In 1988 Smith reported two cases of hypercalciuria with oral calcitriol [19]. Both patients withdrew from the study. There were no reports of renal insufficiency, nephrolithiasis or any other adverse events. 

In 1996 Prystowsky reported hypercalcemia in 2 patients in the UVB phototherapy and calcitriol group, but neither had hypercalciuria [61]. The hypercalcemia resolved with reduction in their intake of calcitriol. Three patients in both the calcitriol and placebo groups developed hypercalciuria (values not provided). There were no reports of renal insufficiency, nephrolithiasis or any other adverse events related to the hypercalcemia or hypercalciuria reported. 

In 1986 Morimoto did not report any adverse events related to treatment with vitamin D [14]. The authors reported “None of the patients in the three groups suffered from any topical or systemic complications or symptoms during these observation periods. Blood and urine analysis showed values within normal limits at all times. Hepatic and renal function, evaluated by measuring the serum levels of glutamic oxaloacetic transferase, glutamic pyruvic transferase, urea nitrogen and creatinine, were within normal ranges and did not change significantly during the observation periods”. Morimoto did report a significant difference between baseline and 3-month calcium levels was noted in the 1(OH)D_3_ and oral calcitriol groups, and for calcitriol in the 1-OHD_3_ group, but all values were within the normal range. 

In 1990 Huckins reported two patients were unable to receive therapeutic doses due to hypercalciuria but did not report any other adverse events related to treatment with oral calcitriol [21].

In 1996 Perez did not report any adverse events related to treatment with oral calcitriol [22]. Perez did report that the mean 24-h urine calcium concentrations (mg/24 h), calcium/creatinine ratios, and the serum calcium levels were significantly increased compared to baseline at 6, 12, 24 and 36 months, but remained within normal limits.

In 2009 and 2010 Osmancevic did not report any adverse events related to treatment with UVB phototherapy or sunshine [63,64]. In 2009 Osmancevic reported serum concentrations of calcium, creatinine, and 1,25-dihydroxyvitamin D3 were unchanged after UVB phototherapy, while iPTH concentrations decreased in the BB group.

In 2010 Ryan did not report any adverse events related to treatment with UVB phototherapy [62]. 

In 2012 and 2019 McCullough did not report any adverse events related to treatment with oral vitamin D_3_ [25,28].

In 2013 Finamor did not report any adverse events related to treatment with oral vitamin D_3_ [26]. The authors reported “Laboratory or clinical signs of toxicity (hypercalcemia, hypercalciuria or kidney dysfunction) were not observed in any of the 25 participants, including a patient with vitiligo who reached a serum concentration of 25OHD_3_ of 202 ng/mL.

The evidence in these studies show that the clinical efficacy and safety of oral and topical vitamin D are comparable to the clinical efficacy and safety of UVB phototherapy and sunshine in the treatment of plaque psoriasis. Several reports have addressed the potential for photo-carcinogenesis as an adverse effect of UVB phototherapy and have not found this to be a risk [115,119]. Several risks associated with UVB phototherapy identified by the American Academy of Dermatology include photoaging, a possible risk of genital tumors in men treated without genital shielding, and UVB-related cataract formation, which can be mitigated by the use of eye goggles, in addition to acute reactions such as erythema, itching, burning and stinging [119]. 

### 4.11. Sunshine and Topical Vitamin D Appear to Work More Quickly Than UVB Phototherapy and Oral Vitamin D

In comparing topical vs oral vitamin D, both Morimoto et al. in 1986 [14] and Smith et al. in 1988 [19] found topical calcitriol resulted in significant clearing of psoriasis plaques within a few weeks, versus a few months with oral 1(OH)D or oral calcitriol. Sunshine was also shown to clear psoriasis plaques within a few weeks compared to a few months for UVB/NB phototherapy by Osmancevic et al. in 2010 [64]. 

### 4.12. Authors Reported Views Support the Safety and Efficacy of Vitamin D for the Treatment of Psoriasis

Krafka 1936 [12]: “If the treatment were at all hazardous or difficult, we would not presume to lay it before the profession. But the treatment is so simple that it should be put to a trial test in the interest of every patient suffering from this obnoxious condition. Certainly, it is worth a fair trial. We leave our results to be tested on a more elaborate scale by the larger clinics”.

Morimoto 1986 [14]: “These data suggest that exogenous active forms of vitamin D_3_ are effective for the treatment of psoriasis, and that the endogenous 1,25-dihydroxyvitamin D level also may be involved in the development of this disease”.

Smith 1988 [19]: “Topical or oral use of 1,25-(OH)_2_D_3_ heralds a new mode of treatment that appears to be both safe and effective for the treatment of psoriasis”.

Perez 1996 [22]: “Oral calcitriol is effective and safe for the treatment of psoriasis”.

Prystowsky 1996 [61]: “Because phototherapy for psoriatic plaques produces changes in keratinocytes similar to those described for 1,25-(OH)2D3 (i.e., slowed proliferation and enhanced differentiation), this raises the possibility that one of the mechanisms of action of UVB may be through enhanced vitamin D metabolism”.

Finamor 2013 [26]: “In summary, the present study suggests that, at least for patients with autoimmune disorders like vitiligo and psoriasis, a daily dose of 35,000 IU of vitamin D_3_ is a safe and effective therapeutic approach for reducing disease activity”.

McCullough previously reported on the long-term safety of daily supplementation of oral vitamin D_3_ in doses ranging from 5000 IU to 60,000 IU/day and found no adverse events after treatment for up to 7 years [28,114]. Several thousand long-term hospitalized patients taking 5000 to 10,000 IU/day were included the review [28]. We recently measured 24 urine calcium and creatinine levels in 16 individuals after long-term supplementation with varying doses of vitamin D. This included measurements in 4 individuals after taking 5000 IU/day for 13 to 94 months, in 9 individuals after taking 10,000 IU/day for 7 to 105 months, in one individual after taking vitamin D_2_ 50,000 IU/day for 51 months reported earlier, in one individual after taking vitamin D_3_ 60,000 IU/day for 67 months, and in one individual after sunbathing periodically for 33 months. Normal 24 urinary calcium excretion was observed in all 16 individuals (unpublished data). 

### 4.13. Estimates of Vitamin D Production in the 1970s Are Much Lower Than Doses Used in the 1930s and 1940s

The first estimates of the physiologic amounts of vitamin D produced in the skin after exposure to UVB radiation were not available until 1977 and were found to be greater than 10,000 IU/day [110]. This data was later confirmed by other investigators [56,111,112,113]. These researchers also noted that the upper limits of daily vitamin D production in the skin from UVB exposure appear to be in the range of 20,000 to 25,000 IU/day [56,111,112,113]. This range is much less than the 60,000 to 600,000 IU/day used successfully clinically in the 1930s and 1940s and is much higher than the upper limit of 4000 IU/day currently recommended by the IOM [55] but is within the upper limit of 10,000 IU/day recommended by the Endocrine Society [56]. 

These estimates of physiologic vitamin D production in the skin may explain why hypercalcemia was often observed as a side effect of treatment with vitamin D in the 1930s and 1940s but was not observed in the oral vitamin D_3_ and oral vitamin D_2_ studies reviewed in this report which used doses ranging from 35,000 to 50,000 IU/day for 6 months to over 2 years. The serum 25(OH)D concentrations associated with the clinical benefits reported in the 1930s and 1940s when supraphysiologic doses of vitamin D were used to cure both tuberculosis [33,34,35,36,37,38,39] and rickets [32,46], and to control asthma [29], psoriasis [12] and rheumatoid arthritis [30,31] are unknown as tests for measuring serum 25(OH)D concentrations were not available until 1971 [255]. The upper limit of daily vitamin D intake that that is clinically effective but does not result in adverse events in the treatment of psoriasis and other vitamin D deficiency linked diseases needs to be further investigated. 

Figure 1 shows the metabolic pathway of calcitriol production from 7-dehydrocholesterol in the skin after exposure to either sunshine or UVB phototherapy. It also illustrates why supplementation with 1(OH)D and calcitriol have no effect on serum 25(OH)D concentrations, as opposed to the dose dependent increase observed after supplementation with oral vitamin D_2_ and oral vitamin D_3_.

UVB radiation in sunshine causes the transformation of 7-dehydrocholesterol in the skin into vitamin D_3_ [18,53,56,111,112,113,258]. Vitamin D_3_ then undergoes a hydroxylation reaction on the 25th carbon to form 25(OH)D, which has a circulating half-life of a few weeks, and is what is measured to determine vitamin D status [132,133,258]. A second hydroxylation reaction on the 1st carbon is required to form calcitriol, which can occur in the skin, kidneys and multiple other organs [18,83,132,133]. 

Both 25OHD and calcitriol circulate in serum in free and carrier protein (albumin and vitamin D binding protein (DBP)) bound forms [259]. The sum of the free and albumin bound forms are referred to bioavailable vitamin D, as both 25OHD and calcitriol dissociate more easily from albumin than from DBP. Vitamin D needs to be in the free form to pass through the lipophilic cell membrane, and it is thought that the free serum 25OHD concentration may reflect its biological actions better than the total serum 25OHD concentration [260]. A recent study in pregnant women found opposite correlations between serum 25OHD and serum calcitriol concentrations with gestational age, bone and lipid biomarkers. Free and bioavailable 25OHD showed a better overall correlation than total 25OHD with these biomarkers, while total serum calcitriol showed a better correlation with the same biomarkers than free or bioavailable calcitriol [259]. This may explain the why “normal” total serum 25OHD concentrations correlate poorly with psoriasis control. However, at this time total serum 25OHD concentrations are usually reported, and more research will be needed to assess whether free or bioavailable 25OHD correlate better with psoriasis control than total serum 25OHD.

A primary action of calcitriol is regulation of gene transcription, which has been shown to be regulated by vitamin D in multiple different cell types and tissues throughout the body [132,133,134,135,136,137,138]. Close to three thousand binding sites for the VDR have been identified in a human cell line [134]. Many cells of the immune system have been shown to require vitamin D for normal cellular metabolism including regulatory T lymphocytes [143], which we hypothesize to be the target cells regulated by vitamin D that control psoriasis and other autoimmune diseases as previously discussed [153,154,155,156,157,158,159,160,161,162,163,164,165,166,167,168,169,170,171,172,173,174,175,176,177,178,179,180,181,182,183,184,185,186,187,188,189,190,191,192,193,194,195,196,197,198,199,200,201,202,203,204,205,206,207,208,209,210,211,212,213,214,215,216,217,218,219,220,221,222,223,224,225,226,227,228,229,230,231,232,233,234,235,236,237,238,239,240,241,242,243,244,245,246,247,248,249,250,251,252,253,254].

Figure 1 also illustrates why treatment with oral or topical calcitriol and 1(OH)D does not affect serum 25(OH)D concentrations, as neither are metabolized into 25(OH)D. In contrast, both oral vitamin D_2_ and oral vitamin D_3_ are metabolized into 25(OH)D and cause an increase in circulating serum 25(OH)D concentrations in a dose dependent manner [28,114,261,262,263,264,265].

## 5. Implications for the Treatment of Other Vitamin D Deficiency Related Diseases with Vitamin D or Phototherapy

These observations raise important questions about the adequacy of serum 25(OH)D concentrations falling between 20 to 100 ng/mL not only for patients suffering from plaque psoriasis, but for those suffering from other diseases that are also strongly linked to vitamin D deficiency. Such diseases are numerous and include asthma, atherosclerosis, autoimmune diseases, cancers, falls, fractures, infections, mortality, myopathies, muscle weakness, neurological disorders, osteomalacia, osteoporosis, and psychiatric disorders [12,29,30,31,32,33,34,35,36,37,38,39,46,53,65,66,67,68,69,70,71,72,73,74,75,76,77,78,79,80,258,261,262,263,264,266,267,268,269,270,271,272,273,274,275,276,277,278,279,280,281,282,283,284,285,286,287,288,289,290,291,292,293,294,295,296,297,298,299,300,301,302,303,304,305,306,307,308,309,310,311,312,313,314,315,316,317,318,319]. Of particular interest currently are adverse outcomes from viral infections such as influenza [65,264,281,289,294,303] and COVID-19 [65,66,67,68,69,70,71,72,73,74,75,76,77,78,79,80], both of which have shown a strong association with vitamin D deficiency. 

Many clinical trials using oral vitamin D supplementation performed and reported in the past 30 years have used daily dosing ranging from 800 IU/day to 4000 IU/day, with few exceeding 4000 IU/day [258,261,262,263,264,266,267,268,269,270,271,272,273,274,275,276,277,278,279,280,281,282,283,284,285,286,287,288,289,290,291,292,293,294,295,296,297,298,299,300,301,302,303,304,305,306,307,308,309,310,311,312,313,314,315,316,317,318,319]. This is well below the range of 10,000 to 25,000 IU/day reported to be produced by sun exposure to the skin, and far below the clinically effective doses of vitamin D used in the 1930s and 1940s. While several clinical trials using daily dosing ranging from 800 IU/day to 4000 IU/day were effective, others showed mixed or negative results. Three such negative trials will be briefly reviewed. Several case reports and clinical trials showing clinical benefits with supplementation with vitamin D in doses > 4000 IU/day will also be reviewed.

### 5.1. Inadequacy of 4000 IU/Day of Vitamin D_3_ in the Treatment of Asthma and of 2000 IU/Day in the Prevention of Cancer

Two recent clinical trials investigating the effects of daily intake of 4000 IU/day of vitamin D_3_ versus placebo on asthma control in adults and children showed no significant clinical benefits [304,318]. The first clinical trial was a 28-week study published in 2014 involving 201 treated adults [304], and the second was a 48-week study published in 2020 involving 96 treated children [318]. Similarly, a 5-year clinical trial comparing the effect of daily intake of 2000 IU/day of vitamin D_3_ versus placebo published in 2019 in preventing invasive cancer or cardiovascular events in 12,927 treated adults found no significant clinical benefit [317]. Mean baseline serum 25(OH)D concentrations in these three clinical trials were 18.8 ng/mL, 22.5 ng/mL and 30.8 ng/mL respectively. On treatment mean serum 25(OH)D concentrations averaged 42 ng/mL in the first study at 12, 20 and 28 weeks (range 6.3 to 97.3 ng/mL at week 12), and in the second study were 57.2, 53.8 and 49.4 ng/mL at weeks 16, 32 and 48 (ranges not indicated). A subgroup of 1644 participants in the third clinical trial had a baseline mean serum 25(OH)D concentration of 29.8 ng/mL which increased to 41.8 ng/mL at one year (range not indicated). No changes were seen in mean serum 25(OH)D concentrations in the placebo groups over the time course of the studies. 

The on-treatment data reported in these clinical trials are similar to the baseline serum 25(OH)D concentrations in the psoriasis reports reviewed in this report, which were insufficient for disease control in psoriasis. This suggests the possibility that daily intakes higher than 2000 to 4000 IU/day of vitamin D_3_ may be needed for treating asthma and for preventing cancer and cardiovascular disease, which is consistent with the observations first reported in the 1930s and 1940s with asthma, psoriasis, rheumatoid arthritis, and tuberculosis, as well as with the psoriasis studies reviewed in this report. It is also consistent with several recent case reports and clinical trials of diseases showing clinical improvement with vitamin D intake > 4000 IU/day.

### 5.2. Case Reports and Clinical Trials of Diseases Showing Clinical Improvement with Vitamin D Intake > 4000 IU/Day

Several case reports and clinical trials published in the past few decades [80,114,272,296,297,303,308,309,310], in addition to the 3 discussed in this report [25,26,28], have shown significant clinical benefits without toxicity with vitamin D supplementation when using doses of vitamin D above 4000 IU/day, and ranging up to 50,000 IU/day for extended periods of time, thus providing further support for this recommendation. This includes significant clinical improvement in:

(1) A 1997 case report of chronic Parkinson’s disease symptoms over the course of a year using 4000 IU/day of 25(OH)D [272], roughly equivalent to 20,000 IU/day of vitamin D_3_ [110];
(2) Control of chronic pain in children suffering from sickle cell disease using 50,000 IU twice weekly of vitamin D_3_ for 8 weeks, followed by once weekly for 32 months in a 2011 case report [296], and subsequently in a weight-based dosing study using 40,000 IU to 100,000 IU/ week for 6 weeks in 20 children in a 6 month placebo controlled trial published in 2012 [297];
(3) Chronic fatigue in a 2014 prospective study of 171 adult patients with low serum 25(OH)D concentrations (<30 ng/mL) using 50,000 IU of vitamin D_2_ three times a week for 5 weeks [303], which averages out to 21,429 IU/day;
(4) Prevention of statin intolerance secondary to myalgia, myositis, myopathy or necrosis in 171 previously statin intolerant patients with low serum 25(OH)D concentrations (<32 ng/mL) using either 50,000 or 100,000 IU/week of vitamin D_2_ for 24 months published in 2015 [308];
(5) 282 patients treated with these same doses in a prospective one-year clinical safety trial from this same group using vitamin D_3_ instead of vitamin D_2_, and again found to be safe in 2016 [309];
(6) Prevention of progression of a case of advanced pancreatic cancer using 50,000 IU/day of vitamin D_3_ for 9 months reported in 2016 [310];
(7) Asthma control in a case of long-standing asthma using 20,000 to 25,000 IU/day of vitamin D_3_ for several years reported in 2019 [28,114];
(8) A non-melanoma skin cancer in an individual taking 60,000 IU/day of vitamin D_3_ for several years reported in 2019 [28,114];
(9) The need for ICU treatment in patients requiring hospitalization due to proven COVID-19 infections in 50 patients treated with 25(OH)D (532 µg (21,280 IU) on day 1; 266 µg (10,640 IU) on days 3 and 7, followed by 266 µg weekly until discharge) which resulted in one ICU admission (2%), versus a group of 26 untreated patients of which thirteen (50%) required admission for care in an ICU [80].

COVID-19 deaths have now passed 2.4 million worldwide [319]. In the United States, there have been over 28 million cases and over 510,000 deaths [319] with no signs of slowing down soon. A recent report analyzing over 190,000 patients infected with COVID-19 in the United States showed a strong correlation with vitamin D deficiency and risk of infection, with a 53% lower SARS-CoV-2 positivity rate among patients with a serum 25(OH)D concentration > 55 ng/mL versus those < 20 ng/mL, and a 43% lower risk for contracting COVID-19 with an increase in serum 25(OH)D concentrations from 20 ng/mL to 55 ng/mL [75], providing further evidence of the urgency to conduct such clinical trials. 

### 5.3. Need to Better Define the Therapeutic Index of Vitamin D

The serum 25(OH)D concentrations in psoriasis patients before and after treatment reviewed in this report suggest revision of current definitions of vitamin D deficiency, insufficiency, sufficiency and toxicity, as well as diseases currently recognized as being responsive or unresponsive to vitamin D supplementation. There is a dearth of clinical trial data examining the clinical utility and toxicity of oral vitamin D supplementation between the conventional dose ranges of 800 to 4000 IU/day and 60,000 to 600,000 IU/day. The supraphysiologic (by current standards) doses of vitamin D used in the 1930s and 1940s were proven clinically effective in treating asthma, rheumatoid arthritis and tuberculosis in addition to psoriasis, but were associated with reversible hypercalcemia and calcium crystal formation after prolonged daily intake [31,35,39,320,321]. Clinical trials in patients suffering from psoriasis and other diseases shown to be strongly linked to vitamin D deficiency using daily oral supplementation between these extremes, particularly dosing encompassing the range of 10,000 to 25,000 IU shown to be produced by adequate daily UVB exposure to the skin, need to be done to clarify the therapeutic index of vitamin D.

## 6. Conclusions and Future Directions

Psoriasis responds safely to treatment with 4 different forms of oral vitamin D: vitamin D_2_, vitamin D_3_, 1-alpha-hydroxyvitaminD_3_, and 1,25-dihydroxyvitamin D_3_ (calcitriol). Pre-treatment serum 25(OH)D_3_ concentrations above 20 ng/mL, ranging up to 67 ng/mL, were common in patients with plaque psoriasis in the oral vitamin D dosing studies reviewed. However, patients showed significant dermatological improvement in their skin without toxicity after daily treatment with four different forms of oral vitamin D. This suggests that serum 25(OH)D_3_ concentrations > 20 ng/mL are not adequate for many patients with plaque psoriasis, even though they are considered adequate for the majority of the population by the IOM [55] and the Endocrine Society [56], calling into question the definition of an adequate serum 25OHD concentration. Pre-treatment serum 25(OH)D_3_ concentrations > 20 ng/mL, ranging up to 88 ng/mL, were also commonly observed in patients with plaque psoriasis in the UVB phototherapy studies reviewed, yet these patients still showed significant improvement in their skin after treatment with UVB phototherapy. This was associated with significantly increased serum 25(OH)D_3_ concentrations post-treatment, with several patients > 100 ng/mL, and ranging up to 159 ng/mL without any adverse events.

Post-treatment serum 25(OH)D_3_ concentrations > 100 ng/mL were also observed in patients with plaque psoriasis safely treated with 35,000 IU/day of vitamin D_3_ for 6 months, and in a patient treated with 50,000 IU/day of vitamin D_2_ for over 3 years, without any adverse events. This was associated with peak serum 25(OH)D_3_ concentrations of 202 ng/mL and 308 ng/mL respectively. The fact that serum 25(OH)D_3_ concentrations > 100 ng/mL have been obtained safely after disease control using UVB phototherapy, 35,000 IU/day of vitamin D_3_, and 50,000 IU/day of vitamin D_2_ calls into question the safe upper limit of serum 25(OH)D_3_ concentrations. Currently, a serum 25(OH)D_3_ concentration > 50 ng/mL is considered potentially dangerous for the majority of the population and is not recommended by the IOM [55] and a serum 25OHD concentration > 100 ng/mL is considered high by the Endocrine Society [56]. In contrast, several reviews [57,58,59] and case reports [60] on vitamin D toxicity have suggested that serum 25OHD concentrations > 100 ng/mL and ranging up to 400 ng/mL may be safe. This needs further clarification. A recent review on vitamin D safety suggests that “Vitamin D is not as toxic as was once thought” [307] 

The exact range of total serum 25OHD concentrations needed to be achieved to improve psoriasis is currently unknown and is speculative at this time. Dose response studies need to be performed with all four forms of oral vitamin D to clarify the time response to treatment with varying doses and the therapeutic index. However, as mentioned elsewhere in this review, current “normal” levels for vitamin sufficiency are not likely to be effective in treating psoriasis. We suggest that total serum 25OHD concentrations closer to 100 ng/mL might be needed when oral vitamin D_2_ or vitamin D_3_ are used. However, because there will be no change in total serum 25OHD concentrations when either 1(OH)D or calcitriol are used, as total serum 25OHD concentrations are not affected when these agents are used (Figure S1, Table S2, Supplementary Materials), measurement of free and bioavailable serum 25OHD may prove to be better markers of disease control in psoriasis, as previously discussed [259,260]. In addition there may be differences in responses between males and females, as a correlation between vitamin D deficiency and insulin resistance was recently reported in females, but not in males [322].

Vitamin D toxicity was absent in the reviewed clinical reports. When it occurs, it is manifested by complications related to hypercalcemia, renal insufficiency, hypercalciuria, calcium crystal formation, and undetectable serum parathyroid hormone concentrations, which have been shown to be reversible by simply stopping the vitamin D and providing supportive care with no long-term sequelae. Several such complications induced by excessive vitamin D intake (hypercalcemia, renal insufficiency, hypercalciuria, and undetectable serum PTH) were shown to resolve when serum 25(OH)D_3_ concentrations dropped below 400 ng/mL in 2 case reports in 2011 after accidental ingestion of massive amounts of vitamin D over a period of 1 to 2 months [60]. Due to labeling and manufacturing errors of over the counter supplements, one patient took 1,864,000 units (46,000 µg) of vitamin D_3_ daily for 2 months and achieved a peak serum 25OHD concentration of 1220 ng/mL. The second took 970,000 units of vitamin D_3_ a day for one month and achieved a peak serum 25OHD concentration of 645 ng/mL. Both recovered uneventfully after cessation of vitamin D intake. Our clinical experience is consistent with this, as we have not observed hypercalcemia, renal insufficiency, hypercalciuria, undetectable serum parathyroid hormone concentrations or any other toxicity in patients with serum 25(OH)D_3_ concentrations ranging from 202 ng/mL to 384 ng/mL [28]. 

Vitamin D has a much safer toxicity profile than methotrexate, cyclosporine and biologics such as Humira and Enbrel, which are more commonly used for the treatment of psoriasis. Biologics are among the most frequently reported drugs for adverse events to the FDA, and many have FDA mandated black box warnings for risk of cancers such as lymphoma, serious infections such as tuberculosis and invasive fungal infections, and death [323,324,325,326,327,328,329,330,331,332,333,334,335]. In contrast, vitamin D was shown in the 1940s to safely cure tuberculosis infections as a single agent using daily oral intake of 100,000 to 150,000 units for 2 to 3 months [33,34,35,36,37,38,39], most likely by turning on genes that make antimicrobial peptides active against TB [39,282,289]. More recently, vitamin D has been shown to have anticancer properties [28,284,287,295,298,301,310], and appears to reduce the risk for developing cancer, and not increase it as biologics do. More clinical research utilizing a range of vitamin D intakes is needed to confirm these findings and to see if a dose response exists in cancer prevention, in light of the recent clinical trial reviewed earlier that showed no clinical benefit in cancer prevention after daily intake of 2000 units of vitamin D_3_ over five years [318]. Vitamin D also appears to have a safer toxicity profile than sunshine, UVB phototherapy, and even acetaminophen, one of the most commonly used over the counter medications, and a leading cause of liver failure [336,337].

The fact that both oral and topical vitamin D were able to produce the same clinical outcomes as seen with sunshine and UVB phototherapy is compelling evidence that the effects of sunshine and UVB phototherapy in treating psoriasis is mediated by vitamin D production in the skin. The mechanism of action explaining how vitamin D works to clear psoriasis skin lesions is currently unknown but appears likely to be related to the documented effects vitamin D has on stimulating the production and maintenance of regulatory T lymphocytes (Tregs), which have been characterized as master regulators of the immune system due to their ability to control autoimmune diseases [239]. This also needs further clarification. However, consistent with this notion, Tregs have been shown to be dysfunctional in a state of vitamin D deficiency, and can have their functional status restored by sunshine, phototherapy and oral or topical vitamin D [153,154,155,156,157,158,159,160,161,162,163,164,165,166,167,168,169,170,171,172,173,174,175,176,177,178,179,180,181,182,183,184,185,186,187,188,189,190,191,192,193,194,195,196,197,198,199,200,201,202,203,204,205,206,207,208,209,210,211,212,213,214,215,216,217,218,219,220,221,222,223,224,225,226,227,228,229,230,231,232,233]. Vitamin D causes the formation of Tregs to occur indirectly through direct effects on antigen presenting cells, which then cause naïve T cells to transform into Tregs [143].

Placebo controlled, blinded clinical trials using oral vitamin D_3_ for the treatment of patients suffering from psoriasis are warranted based on the results of the studies reviewed in this paper. There is strong evidence that physiologic doses in the range of 10,000 to 25,000 IU/day, and ranging up to 50,000 IU/day, should be able to be administered safely in a clinical trial setting. Serum calcium and PTH concentrations, renal function and urine calcium concentrations can be easily monitored and readily corrected without long-term risk if they become abnormal by simply stopping vitamin D supplementation. The vitamin D could then be resumed again at a lower dose to monitor clinical efficacy and safety. There is potentially much to gain if these dose-response clinical trials are successful. Oral vitamin D is the most affordable treatment option for psoriasis by a wide margin, especially when compared to biologics. The potential cost savings to the health care system by the increased use of oral vitamin D in treating psoriasis is enormous, in the range of billions of dollars/year, with improved patient safety and satisfaction. It is not clear why the four forms of oral vitamin D discussed in this review are not currently being used to treat psoriasis, after being endorsed as safe and effective treatments by the authors of the clinical trials reviewed.

The failure of clinical trials that used sub-physiologic doses of vitamin D and achieved inadequate serum 25(OH)D concentrations has created doubt about the importance of vitamin D in the prevention and treatment of human disease. The fear of causing toxicity by using excessive amounts of vitamin D has led to the unintended consequence of causing needless suffering by perpetuating uncontrolled disease states that might otherwise be controlled by sufficient vitamin D intake or exposure to UVB radiation. Clinical trials examining the dose response of oral vitamin D_3_ using 10,000 to 25,000 IU/day or higher may prove beneficial in controlling plaque psoriasis and other vitamin D related diseases without causing harm. It may have major promise in treating COVID-19 infections, where therapy with “higher than usual” doses probably need to be given only short term given the nature of the clinical course of COVID-19 infections. This was shown to be a successful treatment strategy for chronic tuberculosis infections in the 1940s. Current definitions of normal and excessive serum 25OHD concentrations need to be re-evaluated based on the clinical data reviewed in this manuscript. The therapeutic index of vitamin D in the treatment of human disease needs to be better defined. 

## Figures and Tables

**Table 1:** Study year, type of vitamin D, route, dose, duration of treatment, number of patients treated, and number showing significant improvement in the oral vitamin D clinical trials and case reports reviewed.

Study	Type of				100%	Signif	Study
Year	Vit D	Route	Dose/Day	*N*	Improv	Improv	Duration
1936	D2	oral	NA	3	2	3	6 mos
1986	1(OH)D3	oral	1 µg	17	5	13	6 mos
	calcitriol	oral	0.5 µg	4	0	1	6 mos
	calcitriol	topical	0.5 µg/gm	19	3	16	6 mos
1988	calcitriol	oral	0.5 to 2 µg	14	3	10	12 mos
	calcitriol	topical	3 µg/gm	3	3	3	6 weeks
1990	calcitriol	oral	0.5 to 2 µg	9	0	7	6 mos+
1996	calcitriol	oral	0.5 to 4 µg	85	20	47	36 mos
2012	D3	oral	40,000 IU	2	1	2	5 mos
2013	D3	oral	35,000 IU	9	1	9	6 mos
2019	D2	oral	50,000 IU	1	1	1	2+ years
			Total	166	39	112	

Note: NA = not available, *N* = number of patients treated, Signif Improv = significant improvement reported.

**Table 2:** Baseline mean serum 25(OH)D concentrations, range at baseline, number greater than 20 ng/mL at baseline, number greater than 50 ng/mL at baseline, and peak serum 25(OH)D concentrations post-treatment in the vitamin D clinical trials and case reports reviewed.

			Baseline	Baseline	Baseline	Baseline	Post-Tx
Study	Type of		25(OH)D	25(OH)D	25(OH)D	25(OH)D	25(OH)D
Year	Vit D	*N*	Mean	Range	*N* > 20	*N* > 50	Peak ng/mL
1936	D2	3	NA	NA	NA	NA	NA
1986	1(OH)D3	17	23 ± 12	NR	sig #	NR	NR
	calcitriol	4	17 ± 5	NR	sig #	NR	NR
	calcitriol	19	20 ± 10	NR	sig #	NR	NR
1988	calcitriol	14	32.8	8 to 67	9	3	NR
	calcitriol	3	48.5	30 to 67	2	1	NR
1990	calcitriol	9	NR	NR	NR	NR	NR
1996	calcitriol	85	32 ± 18	NR	sig #	sig #	NR
2012	D3	2	26	23 to 29	2	0	
2013	D3	9	15 ± 7	NR	NR	NR	202
2019	D2	1	70	NA	1	1	308
		166					

Note: NA = not available; NR = not reported; 25(OH)D = ng/mL, *N* > is number of measurements above 20 ng/mL or 50 ng/mL, sig # = significant number based on mean ± standard deviation (actual number not reported).

**Table 3:** Study year, type of phototherapy of used in each study, number of patients treated, study duration and number of patients showing significant improvement following treatment.

Study			100%	Signif	Study
Year	Group	*N*	Improv	Improv	Duration
1996	UVB + Placebo	7	NR	7	8 weeks
	UVB + Calcitirol	6	NR	6	8 weeks
2010	UVB	29	29	29	4 mos
	No UVB	29	0	0	4 mos
2009	BB-UVB	26	NR	26	8 to 12 weeks
	NB-UVB	42	NR	42	8 to 12 weeks
2010	BB-UVB	24	NR	24	8 to 12 weeks
	Sunshine	20	NR	20	2 weeks
	Total	183	29	154	

Note: *N* = number of patients treated, NR = not reported, Signif Improv = significant improvement reported.

**Table 4:** Study year, type of phototherapy of used, number of patients treated, baseline and post treatment mean serum 25(OH)D concentrations, and baseline and posttreatment range at of serum 25(OH)D concentrations in the phototherapy clinical trials reviewed.

			Baseline	Post-Tx	Baseline	Post-Tx
Study			25(OH)D	25(OH)D	25(OH)D	25(OH)D
Year	Group	*N*	Mean	Mean	Range **	Range **
1996	UVB + Placebo	7	37.9	96.1	20 to 80	45 to 159
	UVB + Calcitirol	6	27.3	67.1	15 to 40	45 to 123
2010	UVB	29	23 *	51 *	9 to 46	32 to 112
	No UVB	29	12 *	13 *	7 to 42	7 to 33
2009	BB-UVB	26	38 ± 17	69 ± 20	17 to 82	45 to 118
	NB-UVB	42	35 ± 12	55 ± 18	15 to 73	28 to 98
2010	BB-UVB	24	37 ± 17	60 ± 19	18 to 88	25 to 90
	Sunshine	20	23 ± 6	42 ± 6	18 to 42	30 to 60
	Total	183				

Note: *N* = number of patients, * = median, ** = range estimated from figure in manuscript.

**Table 5:** Distribution of serum 25(OH)D concentrations greater than 20 ng/mL, 50 ng/mL and 100 ng/mL at baseline and post-treatment in each phototherapy clinical trial.

			Baseline	Baseline	Post-Tx	Post-Tx	Post-Tx
Study			25(OH)D	25(OH)D	25(OH)D	25(OH)D	25(OH)D
Year	Group	*N*	*N* > 20 ng/mL	*N* > 50 ng/mL	*N* > 20 ng/mL	*N* > 50 ng/mL	*N* > 100 ng/mL
1996	UVB + Placebo	7	7	2	7	6	2
	UVB + Calcitirol	6	4	0	6	4	1
2010	UVB	29	19	0	29	15	1+
	No UVB	29	NR	0	7	0	0
2009	BB-UVB	26	65	5	68	48	3
	NB-UVB	42					
2010	BB-UVB	24	22	3	24	17	0
	Sunshine	20	17	0	20	4	0
	Total	183	134	10	161	94	6

Note: *N* = number of patients.

## Data Availability

Not applicable.

## Supplementary Materials

The following are available online at https://www.mdpi.com/article/10.3390/nu13051511/s1, Table S1: Baseline and 3-month mean serum 25O(H)D3 (ng/ml), calcium (mg/dl) and calcitriol (pg/ml) concentrations in the 3 treatment groups. TableS2: Range and distribution of pre-treatment serum 25(OH)D3 concentrations in the combined group of 15 psoriasis patients treated with oral calcitriol (n = 13) and topical calcitriol (n = 2). Table S3. Mean serum 25(OH)D3, calcium, PTH, calcitriol, creatinine, and 24-hour urine calcium concentra-tions at 0, 6, 12, 24 and 36 months in 88 plaque psoriasis patients treated with oral calcitriol. Table S4. Distribution and mean serum concentrations of 25(OH)D3, PTH, calcium, urea and cre-atinine, and 24-hour urinary calcium in 9 patients with psoriasis and 16 patients with vitiligo pre-and 6 months’ post-treatment with oral vitamin D3 at 35,000 IU/day. Figure S1. Improvement in psoriasis plaques after resuming 40,000 IU/day of vitamin D3 in pa-tient 2, whose disease recurred after stopping vitamin D3. Nearly complete clearing of the plaques occurred by 88 days. The yearly cost of treatment with oral vitamin D3 at a dose of 40,000 IU/day is around $104 per year, based on currently available over the counter pricing at $14/bottle for a USP verified bottle of 400 gel caps with 5000 IU/cap. Table S5. Changes in serum 25(OH)D2, iPTH and calcium concentrations over time in a patient with psoriasis completely cleared on 50,000 IU/day of vitamin D2 for > 42 months. Table S6. Mean, range and distribution of serum 25(OH)D3 concentrations, and mean serum cal-cium and calcitriol concentrations before and after UVB phototherapy in 13 patients with plaque psoriasis also treated with placebo (n = 7) or calcitriol (n = 6). Table S7. Range, median and distribution of serum 25(OH)D3 concentrations pre-and post NB-UVB treatment in 29 psoriasis patients and 29 untreated controls. Table S8. Mean, range and distribution of serum 25(OH)D3 concentrations before and after BB-UVB (n = 26) and NB-UVB (n = 42) phototherapy in 68 patients with psoriasis. Table S9. Mean, range and distribution of serum 25(OH)D3 concentrations pre-and post 8-12 weeks of BB-UVB phototherapy (n = 24 postmenopausal women) or 15 days of whole-body sun-shine (n = 20) exposure in 44 patients with plaque psoriasis.

## References

[1] National Psoriasis Foundation Psoriasis Statistics. https://www.psoriasis.org/content/statistics.

[2] Brezinski E.A., Dhillon J.S., Armstrong A.W. (2015). The economic burden of psoriasis in the United States. J. Am. Acad. Dermatol..

[3] (2019). Psoriasis Treatments. National Psoriasis Foundation. https://www.psoriasis.org/about-psoriasis/treatments.

[4] National Psoriasis Foundation (2019). Moderate to Severe Psoriasis and Psoriatic Arthritis: Biologic Drugs. https://www.psoriasis.org/about-psoriasis/treatments/biologics.

[5] National Psoriasis Foundation (2019). Moderate to Severe Psoriasis and Psoriatic Arthritis: Bio-Similar Medicines. https://www.psoriasis.org/about-psoriasis/treatments/biosimilars.

[6] National Psoriasis Foundation (2019). Oral Treatments. https://www.psoriasis.org/about-psoriasis/treatments/oral-treatments.

[7] National Psoriasis Foundation (2019). Traditional Systemic Medications. https://www.psoriasis.org/about-psoriasis/treatments/systemics.

[8] National Psoriasis Foundation (2018). Phototherapy. https://www.psoriasis.org/about-psoriasis/treatments/phototherapy.

[9] National Psoriasis Foundation (2019). Topical Treatments. https://www.psoriasis.org/about-psoriasis/treatments/topicals.

[10] Menter A., Strober B.E., Kaplan D.H., Kivelevitch D., Prater E.F., Stoff B., Armstrong A.W., Connor C., Codoro K.M., Davis D.M.R. (2019). Joint AAD-NPF guidelines of care for the management and treatment of psoriasis with biologics. J. Am. Acad. Dermatol..

[11] Elmets C.A., Leonardi C.L., Davis D.M.R., Gelfand J.M., Lichten J., Mehta N.N. (2019). Joint AAD-NPF guidelines of care for the management and treatment of psoriasis with awareness and attention to comorbidities. J. Am. Acad. Dermatol..

[12] Krafka J. (1936). A Simple Treatment for Psoriasis. J. Clin. Lab. Med..

[13] Morimoto S., Kumahara Y. (1985). A Patient with psoriasis cured by 1 alpha-hydroxyvitamin D3. Med. J. Osaka Univ..

[14] Morimoto S., Yoshikawa K., Kozuka T., Kitano Y., Imanaka S., Fukuo K., Koh E., Kumahara Y. (1986). An open study of vitamin D3 treatment in psoriasis vulgaris. Br. J. Dermatol..

[15] Takamoto S., Onishi T., Moromoto S., Imanaka S., Yukawa S., Kozuka T., Kitano Y., Seino Y., Kumahara Y. (1986). Effect of 1 alpha-hydroxycholecalciferol on psoriasis vulgaris: A pilot study. Calcif. Tissue Int..

[16] Morimoto S., Yoshikawa K., Kozuka T., Kitano Y., Imanaka S., Fukuo K., Koh E., Onishi T., Kumahara Y. (1987). Treatment of psoriasis vulgaris with oral 1 alpha,25-dihydroxyvitamin D3—Report of two cases. J. Dermatol..

[17] Morimoto S., Yoshikawa K. (1989). Psoriasis and vitamin D3. A review of our experience. Arch. Dermatol..

[18] Holick M.F., Smith E., Pincus S. (1987). Skin as the Site of Vitamin D Synthesis and Target Tissue for 1,25-Dihydroxyvitamin D3 Use of Calcitriol (1,25-Dihydroxyvitamin D3) for Treatment of Psoriasis. Arch. Dermatol..

[19] Smith E.L., Pincus S.H., Donovan L., Holick M.F. (1988). A novel approach for the evaluation and treatment of psoriasis. Oral or topical use of 1,25-dihydroxyvitamin D3 can be a safe and effective therapy for psoriasis. J. Am. Acad. Dermatol..

[20] Holick M.F. (1989). Will 1,25-Dihydroxyvitamin D3, MC 903, and Their Analogues Herald a New Pharmacologic Era for the Treatment of Psoriasis?. Arch. Dermatol..

[21] Huckins D., Felson D., Holick M.F. (1990). Treatment of Psoriatic Arthritis with Oral 1,25-dihydroxyvitamin D3: A Pilot Study. Arthritis Rheum..

[22] Perez A., Raab R., Chen T.C., Turner A., Holick M.F. (1996). Safety and efficacy of oral calcitriol (1,25-dihydroxyvitamin D3) for the treatment of psoriasis. Br. J. Dermatol..

[23] Holick M.F. (1994). McCollum Award Lecture, 1994: Vitamin D-new horizons for the 21st century. Am. J. Clin. Nutr..

[24] Ezquerra G.M., Regaña M.S., Millet P.U. (2007). Combination of acetretin and oral calcitriol for treatment of plaque-type psoriasis. Acta Derm. Venereol..

[25] McCullough P.J., Arnold-Long M. Marked improvement in psoriasis skin lesions within 5 months in two patients after treatment with oral vitamin D3 in doses ranging from 10,000 international units (IU’s) to 40,000 IUs daily. Poster Presentation. Proceedings of the 15th Vitamin D Workshop.

[26] Finamor D.C., Sinigaglia-Coimbra R., Neves L.C.M., Guiterrez M., Silva J.J., Torres L.D., Surano F., Neto D.J., Novo N.F., Juliano Y. (2013). A pilot study assessing the effect of prolonged administration of high daily doses of vitamin D on the clinical course of vitiligo and psoriasis. Derm. Endocrinol..

[27] Kamangari F., Koo J., Heller M., Lee E., Bhutani T. (2013). Oral vitamin D, still a viable treatment option for psoriasis. J. Dermatol. Treat..

[28] McCullough P.J., Lehrer D.S., Amend J. (2019). Daily oral dosing of vitamin D3 using 5000 to 50,000 international units a day in long-term hospitalized patients: Insights from a seven year experience. J. Steroid Biochem. Mol. Biol..

[29] Rappaport B.Z., Reed C.I., Hathaway M.L., Struck H.C. (1935). The Treatment of Hay Fever and Asthma with Viosterol of High Potency. J. Allergy..

[30] Dreyer I., Reed C. (1935). The Treatment of Arthritis with Massive Doses of Vitamin D. Arch. Phys. Ther..

[31] Howard J.E., Meyer R.J. (1948). Intoxication with vitamin D. J. Clin. Endocrinol..

[32] Parks E.A. (1940). The therapy of rickets. JAMA.

[33] Dowling G.B., Prosser Thomas E.W. (1945). Lupus vulgaris treated with calciferol. Proc. R. Soc. Med..

[34] Dowling G.B., Prosser Thomas E.W., Wallace H.J. (1946). Lupus Vulgaris treated with Calciferol. Proc. R. Soc. Med..

[35] Dowling G.B., Prosser E.W. (1946). Treatment of lupus vulgaris with Calciferol. Lancet.

[36] Raab W. (1946). Vitamin D—Its bactericidal action. Chest.

[37] Michelson H.E., Steves R.J. (1947). Treatment of cutaneous tuberculosis with large doses of vitamin D. Arch. Derm. Syphilol..

[38] Tomlinson K.M. (1948). Calcium content of skin in lupus vulgaris treated with Calciferol. Lancet.

[39] McCullough P.J., Lehrer D.S. (2018). Vitamin D, cod liver oil, sunshine and phototherapy: Safe, effective and forgotten tools for treating and curing tuberculosis infections—A comprehensive review. J. Steroid Biochem. Mol. Biol..

[40] Grzybowski A., Pietrzak K. (2012). From patient to discoverer—Niels Ryberg Finsen (1860–1904)—The founder of phototherapy in dermatology. Clin. Dermatol..

[41] Honigsmann H. (2013). History of phototherapy in dermatology. Photochem. Photobiol. Sci..

[42] Honigsmann H. (2013). Phototherapy. J. Invest Dermatol..

[43] Grzybowski A., Sak J., Pawlikowski J. (2016). A brief report on the history of phototherapy. Clin Dermatol..

[44] Matos T.R., Sheth V. (2016). The symbiosis of phototherapy and photoimmunology. Clin. Dermatol..

[45] Hess A.F., Unger L.J. (1921). The cure of infantile rickets by sunshine. JAMA.

[46] Rajakumar K. (2003). Vitamin D, cod-liver oil, sunlight, and rickets: A historical perspective. Pediatrics.

[47] Williams C.J.B. (1849). On the use and administration of cod-liver oil in pulmonary consumption. Lond. J Med. Jan..

[48] Morner K.A.H. (1903). Nobel Prize for Physiology or Medicine Award Ceremony Speech. http://www.nobelprize.org/nobel_prizes/medicine/laureates/1903/press.html.

[49] Biography of Dr Neils Ryberg-Finsen. https://www.nobelprize.org/nobel_prizes/medicine/laureates/1903/finsen-bio.html.

[50] Aitken R. (1937). Lupus vulgaris with special reference to its treatment with the finsenhomholt lamp. Br. Med. J..

[51] Masten A.R. (1937). Good Climate—An Asset in the Treatment of Tuberculosis. Chest.

[52] Van Der Lugt L., Rottier P.B. (1958). Finsen therapy and vitamin D. Acta Derm. Venerol..

[53] Tavera-Mendoza L.E., White J.H. (2007). Cell Defenses and the Sunshine Vitamin. Sci. Am..

[54] Gotzsche P.C. (2011). Niels Finsen’s treatment for lupus vulgaris. J. R. Soc. Med..

[55] Ross A.C., Taylor C.L., Yaktine A.L., Del Valle H.B. (2011). Dietary Reference Intakes for Calcium and Vitamin D.

[56] Holick M., Binkley N.C., Bischoff-Ferrari H.A., Gordon C.M., Hanley D.A., Heaney R.P., Murad M.H., Weaver C.M. (2011). Evaluation, Treatment, and Prevention of Vitamin D Deficiency: An Endocrine Society Clinical Practice Guideline. J. Clin. Endocrinol. Metab..

[57] Vieth R. (1999). Vitamin D supplementation, 25-hydroxyvitamin D concentrations, and safety. Am. J. Clin. Nutr..

[58] Hathcock J.N., Shao A., Vieth R., Heaney R.P. (2007). Risk assessment for vitamin D. Am. J. Clin. Nutr..

[59] Jones G. (2008). Pharmacokinetics of vitamin D toxicity. Am. J. Clin. Nutr..

[60] Araki T., Holick M.F., Alfonso B.D., Charlap E., Romero C.M., Rizk D., Newman L.G. (2011). Vitamin D Intoxication with Severe Hypercalcemia due to Manufacturing and Labeling Errors of Two Dietary Supplements Made in the United States. J. Clin. Endocrinol. Metab..

[61] Prystowsky J.H., Muzio P.J., Sevran S., Clemens T.L. (1996). Effect of UVB phototherapy and oral calcitriol (1,25-dihydroxyvitamin D3) on vitamin D photosynthesis in patients with psoriasis. J. Am. Acad. Dermatol..

[62] Ryan C., Moran B., McKenna M.J., Murray B.F., Brady J., Collins P., Rogers S., Kirby B. (2010). The Effect of Narrowband UV-B Treatment for Psoriasis on Vitamin D Status during Wintertime in Ireland. Arch. Dermatol..

[63] Osmancevic A., Landin-Wilhelmsen K., Larko O., Wennberg A.M., Krogstad A.L. (2009). Vitamin D production in psoriasis patients increases less with narrowband than with broadband ultraviolet B phototherapy. Photodermatol. Photoimmunol. Photomed..

[64] Osmancevic A., Landin-Wilhelmsen K., Larko O., Krogstad A.L. (2010). Vitamin D status in psoriasis patients during different treatments with phototherapy. J. Photochem. Photobiol. B Biol..

[65] Grant W.B., Lahore H., McDonnell S.L., Baggerly C.A., French C.B., Aliano J.L., Bhattoa H.P. (2020). Evidence that Vitamin D Supplementation Could Reduce Risk of Influenza and COVID-19 Infections and Deaths. Nutrients.

[66] Carpagnano G.E., Di Lecce V., Quaranta V.N., Zito A., Buonamico E., Di Gioia G. (2021). Vitamin D deficiency as a predictor of poor prognosis in patients with acute respiratory failure due to COVID-19. J. Endocrinol. Investig..

[67] Vyas N., Kurian S.J., Bagchi D., Manu M.K., Saravu K., Unnikrishnan M.K., Mukhopadhyay C., Rao M., Miraj S.S. (2020). Vitamin D in Prevention and Treatment of COVID-19: Current Perspective and Future Prospects. J. Am. Coll. Nutr..

[68] Benskin L.L. (2020). A Basic Review of the Preliminary Evidence that COVID-19 Risk and Severity is Increased in Vitamin D Deficiency. Front. Public Health.

[69] Crane-Godreau M.A., Clem K.J., Payne P., Fiering S. (2020). Vitamin D Deficiency and Air Pollution Exacerbate COVID-19 Through Suppression of Antiviral Peptide LL37. Front. Public Health.

[70] Manson J.E., Bassuk S.S. (2020). Commentary. Eliminating vitamin D deficiency during the COVID-19 pandemic: A call to action. Metab. Clin. Exp..

[71] Giménez V.M.M., Inserra F., Ferder L., García J., Manucha W. (2021). Vitamin D deficiency in African Americans is associated with a high risk of severe disease and mortality by SARS-CoV-2. J. Hum. Hypertens..

[72] Baktash V., Hosack T., Patel N., Shah S., Pirab K., Van Den Abbeele K., Mandal A.K.J., Missouris C.G. (2020). Vitamin D status and outcomes for hospitalized older patients with COVID-19. Postgrad. Med. J..

[73] Davies G., Garami A.R., Byers J. (2020). Evidence Supports a Causal Model for Vitamin D in COVID-19 Outcomes. MedRxiv.

[74] Slominski R.M., Stefan J., Athar M., Holick M.F., Jetten A.M., Raman C., Slominski A.T. (2020). COVID-19 and Vitamin D: A lesson from the skin. Exp. Dermatol..

[75] Kaufman H.W., Niles J.K., Kroll M.H., Bi C., Holick M.F. (2020). SARS-CoV-2 positivity rates associated with circulating 25-hydroxyvitamin D levels. PLoS ONE.

[76] Slominski A.T., Slominski R.M., Goepfert P.A., Kim T., Holick M.F., Jetten A.M., Raman C. (2020). Reply to Jakovac and to Rocha et al.: Can vitamin D prevent or manage COVID-19 illness?. Am. J. Physiol. Endocrinol. Metab..

[77] Maghbooli Z., Sahraian M.A., Ebrahimi M., Pazoki M., Kafan S., Tabriz H.M., Hadadi A., Montazeri M., Nasiri M., Shirvani A. (2020). Vitamin D sufficiency, a serum 25- hydroxyvitamin D at least 30 ng/mL reduced risk for adverse clinical outcomes in patients with COVID-19 infection. PLoS ONE.

[78] Hadizadeh F. (2021). Supplementation with vitamin D in the COVID-19 pandemic?. Nutr. Rev..

[79] Ferder L., Martín Giménez V.M., Inserra F., Tajer C., Antonietti L., Mariani J., Manucha W. (2020). Vitamin D Supplementation As A Rational Pharmacological Approach In The Covid-19 Pandemic. Am. J. Physiol. Lung Cell. Mol. Physiol..

[80] Castillo M.E., Costa L.M.E., Barrios J.M.V., Díaz J.F.A., Miranda J.L., Bouillon R., Gomez J.M.Q. (2020). Effect of calcifediol treatment and best available therapy versus best available therapy on intensive care unit admission and mortality among patients hospitalized for COVID-19: A pilot randomized clinical study. J. Steroid Biochem. Mol. Biol..

[81] Morimoto S., Onishi T., Imanaka S., Yukawa H., Kozuka T., Kitano Y., Yoshikawa K., Kumahara Y. (1986). Topical administration of 1,25-dihydroxyvitamin D3 for psoriasis: Report of five cases. Calcif. Tissue Int..

[82] Lugo-Somolinos A., Sanchez J.L., Haddock L. (1990). Efficacy of 1, alpha 25-dihidroxyvitamin D (Calcitriol) in the treatment of psoriasis vulgaris: An open study. Bol. Asoc. Med. Puerto Rico.

[83] Bikle D.D., Nemanic M.K., Gee E., Elias P. (1986). 1,25-Dihydroxyvitamin D3 production by human keratinocytes. Kinetics and regulation. J. Clin. Investig..

[84] Milde P., Hauser U., Simon T., Mall G., Ernst V., Haussler M.R., Frosch P., Rauterberg E.W. (1991). Expression of 1,25-dihydroxyvitamin D3 receptors in normal and psoriatic skin. J. Invest. Dermatol..

[85] Araujo O.E., Flowers F.P., Brown K.D. (1991). Vitamin D Therapy in Psoriasis. DICP Ann. Pharmacother..

[86] Berth-Jones J., Hutchinson P.E. (1992). Vitamin D analogues and psoriasis. Br. J. Dermatol..

[87] Boisseau-Gersaud A.M., Legrain V., Hehunstre J.P., Maleville J., Taieb A. (1993). Treatment of psoriasis by oral calcitriol. A study of 5 cases and review of the literature. Ann. Dermatol. Venereol..

[88] El-Azhary R.A., Peters M.S., Pittelkow M.R., Kao P.C., Muller S.A. (1993). Efficacy of Vitamin D_3_ Derivatives in the Treatment of Psoriasis Vulgaris: A Preliminary Report. Mayo Clin. Proc..

[89] Samini M., Seirafi H., Eftekhari H.R. (1998). Efficacy and safety of topical cholecalciferol, calcitriol, and calcipitol in the treatment of plaque psoriasis: A comparative study. Acta Med. Iran..

[90] Takahashi H., Ibe M., Kinouchi M., Ishida-Yamamoto A., Hashimoto Y., Lizuka H. (2003). Similarly potent action of 1,25-dihydroxyvitamin D3 and its analogues, tacalcitol, calcipotriol, and maxacalcitol on normal human keratinocyte proliferation and differentiation. J. Dermatol. Sci..

[91] Lebwohl M., Menter A., Weiss J., Clark S.D., Flores J., Powers J., Balin A.K., Kempers S., Glinert R.J., Fleming T. (2007). Calcitriol 3 microg/g ointment in the management of mild to moderate plaque type psoriasis: Results from 2 placebo-controlled, multicenter, randomized double-blind, clinical studies. J. Drugs Dermatol..

[92] Gold L.F. (2009). Calcitriol ointment: Optimizing psoriasis therapy. J. Drugs Dermatol..

[93] Abramovitz W. (2009). Calcitriol 3 microg/g ointment: An effective and safe addition to the armamentarium in topical psoriasis therapy. J. Drugs Dermatol..

[94] Kircik L. (2009). Efficacy and safety of topical calcitriol 3 microg/g ointment, a new topical therapy for chronic plaque psoriasis. J. Drugs Dermatol..

[95] Lehmann B. (2009). Role of the vitamin D3 pathway in healthy and diseased skin—Facts, contradictions and hypotheses. Exp. Dermatol..

[96] Gaal J., Lakos G., Zodoray P., Kiss J., Horvath I., Horkay E., Nagy G., Szegedi A. (2009). Immunological and clinical effects of alphacalcidol in patients with psoriatic arthropathy: Results of an open, follow-up pilot study. Acta Derm. Venereol..

[97] O’Neill J.L., Feldman S.R. (2010). Vitamin D analogue-based therapies for psoriasis. Drugs Today.

[98] Laws P.M., Young H.S. (2010). Topical treatment of psoriasis. Expert Opin. Pharmacother..

[99] Lehmann B., Meurer M. (2010). Vitamin D metabolism. Dermatol. Ther..

[100] Oquendo M., Abramovits W., Morrell P. (2012). Topical vitamin D analogs available to treat psoriasis. Skinmed.

[101] Lebwohl M., Preston N., Gottschalk R.W. (2012). Impact of Baseline Disease Severity Over 26 and 52 Weeks of Treatment with Calcitriol Ointment 3mcg/g in Patients with Mild-to-moderate Plaque Psoriasis. J. Clin. Aesthetic Dermatol..

[102] Sawyer L., Samarasekera E.J., Wonderling D., Smith C.H. (2013). Topical therapies for the treatment of localized plaque psoriasis in primary care: A cost-effectiveness analysis. Br. J. Dermatol..

[103] Soleymani T., Hung T., Soung J. (2015). The role of vitamin D in psoriasis: A review. Int. J. Dermatol..

[104] Wadhwa B., Relhan V., Kochar A.M., Garg V.K. (2015). Vitamin D and skin diseases: A review. Indian J. Dermatol. Venereol. Leprol..

[105] Reichrath J., Zouboulis C.C., Vogt T., Holick M.F. (2016). Targeting the vitamin D endocrine system (VDES) for the management of inflammatory and malignant skin diseases: An historical view and outlook. Rev. Endocr. Metab. Disord..

[106] Barrea L., Savanelli M.C., Somma C., Napolitano M., Megna A., Colao A., Savastano S. (2017). Vitamin D and its role in psoriasis: An overview of the dermatologist and nutritionist. Rev. Endocr. Metab. Disord..

[107] Hambly R., Kirby B. (2017). The relevance of serum vitamin D in psoriasis: A review. Arch. Dermatol. Res..

[108] Paul C., Leonardi C., Menter A. (2017). Calcipotriol Plus Betamethasone Dipropionate Aerosol Foam in Patients with Moderate-to-Severe Psoriasis: Sub-Group Analysis of the PSO-ABLE Study. Am. J. Clin. Dermatol..

[109] Umar M., Sastry K.S., Al Ali F., Al-Khulaifi M., Wang E., Chouchane A.I. (2018). Vitamin D and the Pathophysiology of Inflammatory Skin Diseases. Skin Pharmacol. Physiol..

[110] Stamp T.C.B., Haddad J.G., Twigg C.A. (1977). Comparison of oral 25-hydroxycholecalciferol, vitamin D, and ultraviolet light as determinants of circulating 25-hydroxyvitamin D. Lancet.

[111] Holick M. (1995). Environmental factors that influence the cutaneous production of vitamin D. Am. J. Clin. Nutr..

[112] Holick M.F., Chen T.C., Lu Z., Sauter E. (2007). Vitamin D and Skin Physiology: A D-Lightful Story. J. Bone Miner. Res..

[113] Holick M.F. (2011). Vitamin D: A D-Lightful Solution for Health. J. Investig. Med..

[114] McCullough P.J., Amend J. (2017). Results of daily oral dosing with up to 60,000 international units of vitamin D3 for 2 to 6 years in 3 adult males. J. Steroid Biochem. Mol. Biol..

[115] Hearn R.M.R., Kerr A.C., Rahim K.F., Ferguson J., Dawe R.S. (2008). Incidence of skin cancers in 3867 patients treated with narrow-band ultraviolet B phototherapy. Br. J. Dermatol..

[116] Koek M., Buskens E., van Weelden H., Steegmans P.H.A., Bruijnzeel-Koomen C.A.F.M., Sigurdsson V. (2009). Home versus outpatient ultraviolet B phototherapy for mild to severe psoriasis: Pragmatic multicenter randomized controlled non-inferiority trial (PLUTO study). BMJ.

[117] Koek M., Sigurdsson V., van Weelden H., Steegmans P.H.A., Bruijnzeel-Koomen C.A.F.M., Buskens E. (2010). Cost effectiveness of home ultraviolet B phototherapy for psoriasis: Economic evaluation of a randomized controlled trial (PLUTO study). BMJ.

[118] Vahavihu K., Ala-Houhala M., Peric M., Karisola P., Kautiainen H., Hasan T., Snellman E., Alenius H., Schauber J., Renunala T. (2010). Narrowband ultraviolet B treatment improves vitamin D balance and alters antimicrobial peptide expression in skin lesions of psoriasis and atopic dermatitis. Br. J. Dermatol..

[119] Menter A., Korman N.J., Elmets C.A., Feldman S.R., Gelfand J.M., Gordon K.B., Gottlieb A., Koo J.Y.M., Lebwohl M., Lim H.W. (2010). Guidelines of care for the management of psoriasis and psoriatic arthritis: Section 5. Guidelines of care for the treatment of psoriasis with phototherapy and photochemotherapy. J. Am. Acad. Dermatol..

[120] Takahashi H., Tsuji H., Ishida-Yamamoto A., Lizuka H. (2013). Comparison of clinical effects of psoriasis treatment regimens among calcipotriol alone, narrowband ultraviolet B phototherapy alone, combination of calcipotriol and narrowband ultraviolet B phototherapy once a week, and combination of calcipotriol and narrowband ultraviolet B phototherapy more than twice a week. J. Dermatol..

[121] Wong T., Hsu L., Liao W. (2013). Phototherapy in Psoriasis: A Review of Mechanisms of Action. J. Cutan. Med. Surg..

[122] Shors A., Williams L., Fishman P. (2014). Cost of prevalent psoriasis. J. Am. Acad. Dermatol..

[123] Lim H.W., Silpa-archa N., Amadi U., Menter A., Van Vorhees A.S., Lebwohl M. (2015). Phototherapy in dermatology: A call for action. J. Am. Acad. Dermatol..

[124] Foerster J., Boswell K., West J., Cameron H., Fleming C., Ibbotson S., Dawe R. (2017). Narrowband UVB treatment is highly effective and causes a strong reduction in the use of steroid and other creams in psoriasis patients in clinical practice. PLoS ONE.

[125] Reichrath J., Saternusa R., Vogta T. (2017). Challenge and perspective: The relevance of ultraviolet (UV) radiation and the vitamin D endocrine system (VDES) for psoriasis and other inflammatory skin diseases. Photochem. Photobiol. Sci..

[126] Boswell K., Cameron H., West J., Fleming C., Ibbotson S., Dawe R., Foerster J. (2018). Narrowband ultraviolet B treatment for psoriasis is highly economical and causes significant savings in cost for topical treatments. Br. J. Dermatol..

[127] Hyde K., Cardwell L.A., Stotts R., Feldman S.R. (2018). Psoriasis Treatment Cost Comparison: Biologics Versus Home Phototherapy. Am. J. Pharm. Benefits.

[128] Tanew A., Lim H.W. (2018). Phototherapy for psoriasis—Outdated or underused?. Br. J. Dermatol..

[129] Kimball S.M., Lee J., Vieth R. (2018). Sunbeds with UVB radiation can produce physiological levels of serum 25-Hydroxyvitamin D in healthy volunteers. Derm. Endocrinol..

[130] Hart P.H., Norval M., Byrne S.N., Rhodes L.E. (2019). Exposure to Ultraviolet Radiation in the Modulation of Human Diseases. Annu. Rev. Pathol. Mech. Dis..

[131] Ogawa E., Sato Y., Minagawa A., Okuyama R. (2018). Pathogenesis of psoriasis and development of treatment. J. Dermatol..

[132] Norman A. (2006). Minireview: Vitamin D receptor: New assignments for an already busy receptor. Endocrinology.

[133] Norman A., Bouillon R. (2010). Vitamin D nutritional policy needs a vision for the future. Exp. Biol. Med..

[134] Ramagopalan S.V., Heger A., Berlanga A.J., Maugeri N.J., Lincoln M.R., Burrell A., Handunnetthi L., Handel A.E., Disanto G., Orton S.M. (2010). A ChIP-seq defined genome-wide map of vitamin D receptor binding: Associations with disease and evolution. Genome Res..

[135] Pike J.W. (2011). Genome-wide principles of gene regulation by the vitamin D receptor and it’s activating ligand. Mol. Cell. Endocrinol..

[136] Haussler M.R., Jurutka P.W., Mizwicki M., Norman A.W. (2011). Vitamin D receptor (VDR)-mediated actions of 1alpha, 25(OH)2vitamin D3: Genomic and non-genomic mechanisms. Best Pract. Res. Clin. Endocrinol. Metab..

[137] Pike J.W., Christakos S. (2017). Biology and Mechanisms of Action of the Vitamin D Hormone. Endocrinol. Metab. Clin..

[138] Shirvani A., Kalajian T.A., Song A., Holick M.F. (2019). Disassociation of Vitamin D’s Calcemic Activity and Non-calcemic Genomic Activity and Individual Responsiveness: A Randomized Controlled Double-Blind Clinical Trial. Sci. Rep..

[139] Penna G., Amuchastegui S., Giarrantana N., Daniel K.C., Vulcano M., Sozzani S., Adorini L. (2007). 1,25-Dihydroxyvitamin D3 Selectively Modulates Tolerogenic Properties in Myeloid but Not Plasmacytoid Dendritic Cells. J. Immunol..

[140] Hewison M. (2008). Vitamin D and innate immunity. Curr. Opin. Investig. Drugs.

[141] Sezeles L., Keresztes G., Torocsik D., Balajthy Z., Krenacs L., Poliska S., Steinmeyer A., Zuegel U., Pruenster M., Rot A. (2009). 1,25-Dihydroxyvitamin D3 Is an Autonomous Regulator of the Transcriptional Changes Leading to a Tolerogenic Dendritic Cell Phenotype. J. Immunol..

[142] Von Essen M.R., Kongsbak M., Schjerling P., Olgaard K., Ødum N., Geisler C. (2010). Vitamin D controls T cell antigen receptor signaling and activation of human T cells. Nat. Immunol..

[143] Coussens A. (2011). Immunomodulatory Actions of Vitamin D Metabolites and their Potential Relevance to Human Lung Disease. Curr. Respir. Med. Rev..

[144] Khoo A., Joosten I., Michels M., Woestenenk R., Preijers F., He X., Netea M.H., van der Ven A.J.A.M., Koenen H.J.P.M. (2011). 1,25-Dihydroxyvitamin D3 inhibits proliferation but not the suppressive function of regulatory T cells in the absence of antigen-presenting cells. Immunology.

[145] Hewison M. (2011). Vitamin D and innate and adaptive immunity. Vitam. Horm..

[146] Peelen E., Knippenberg S., Muris A.H., Thewissen M., Smolders J., Tervaert J.W., Hupperts R., Damoiseaux J. (2011). Effects of vitamin D on the peripheral adaptive immune system: A review. Autoimmun. Rev..

[147] Hewison M. (2012). An update of vitamin D and human immunity. Clin. Endocrinol..

[148] Nikolic T., Roep B.O. (2013). Regulatory multitasking of tolerogenic dendritic cells—lessons taken from vitamin D3-treated tolerogenic dendritic cells. Front. Immunol..

[149] Brosbøl-Ravnborg A., Bundgaard B., Hoollsberg P. (2013). Synergy between Vitamin D3 and Toll-Like Receptor Agonists Regulates Human Dendritic Cell Response during Maturation. Clin. Dev. Immunol..

[150] Chun R.F., Liu P.T., Modlin R.L., Adams J.S., Hewison M. (2014). Impact of vitamin D on immune function:lessons learned from genome-wide analysis. Front. Physiol. Integr. Physiol..

[151] Bscheider M., Butcher E.C. (2016). Vitamin D immunoregulation through dendritic cells. Immunology.

[152] Vanherwegen A., Gysemans C., Mathieu C. (2017). Regulation of Immune Function by Vitamin D and Its Use in Diseases of Immunity. Endocrinol. Metab. Clin..

[153] Gorman S., Kuritsky L.A., Judge M.A., Dixon K.M., McGlade J.P., Mason R.S., Finlay J.J., Hart P.H. (2007). Topically Applied 1,25-Dihydroxyvitamin D3 Enhances the Suppressive Activity of CD4 + CD25+ Cells in the Draining Lymph Nodes. J. Immunol..

[154] Ghoreishi M., Bach P., Obst J., Komba M., Fleet J.C., Dutz J.P. (2009). Expansion of Antigen-Specific Regulatory T Cells with the Topical Vitamin D Analog Calcipotriol. J. Immunol..

[155] Gorman S., Judge M., Hart P. (2010). Immune-modifying properties of topical vitamin D: Focus on dendritic cells and T cells. J. Steroid Biochem. Mol. Biol..

[156] Bakdash G., Schneider L.P., van Capel T., Kapsenberg M.L., Teunissen M., de Jong E. (2013). Intradermal application of vitamin D3 increases migration of CD14+ dermal dendritic cells and promotes the development of Foxp3+ regulatory T cells. Hum. Vaccines Immunother..

[157] Lovatoa P., Norsgaarda H., Tokurac Y., Røpke M.A. (2016). Calcipotriol and betamethasone dipropionate exert additive inhibitory effects on the cytokine expression of inflammatory dendritic cell-Th17 cell axis in psoriasis. J. Dermatol. Sci..

[158] Hau C.S., Shimizu T., Tada Y., Kamata M., Takeoka S., Shibata S., Mitsut A., Asano Y., Sugaya M., Kadono T. (2018). The vitamin D_3_ analog, maxacalcitol, reduces psoriasiform skin inflammation by inducing regulatory T cells and downregulating IL-23 and IL-17 production. J. Dermatol. Sci..

[159] Adorini L., Giarratana N., Penna G. (2004). Pharmacological induction of tolerogenic dendritic cells and regulatory T cells. Semin. Immunol..

[160] Xystrakis E., Kusumakar S., Boswell S., Peek E., Urry Z., Richards D.F., Adikibi T., Pridgeon C., Dallman M., Loke T. (2006). Reversing the defective induction of IL-10 secreting regulatory T cells in glucocorticoid-resistant asthma patients. J. Clin. Investig..

[161] Ardalan M., Maljaei H., Shoja M.M., Piri A.R., Khosroshahi H.T., Noshad H., Argani H. (2007). Calcitriol Started in the Donor, Expands the Population of CD4+ CD25+ T Cells in Renal Transplant Recipients. Transplant. Proc..

[162] Jeffery L.E., Burke F., Mura M. (2009). 1,25-Dihydroxyvitamin D3 and IL-2 Combine to Inhibit T Cell Production of Inflammatory Cytokines and Promote Development of Regulatory T Cells Expressing CTLA-4 and FoxP3. J. Immunol..

[163] Gorman S., Judge M.A., Hart P.H. (2010). Gene Regulation by 1,25-Dihydroxyvitamin D3 in CD4+ CD25+ Cells Is Enabled by IL-2. J. Investig. Dermatol..

[164] Gorman S., Judge M.A., Burchell J.T., Turner D.J., Hart P.H. (2010). 1,25-dihydroxyvitamin D3 enhances the ability of transferred CD4+ CD25+ cells to modulate T helper type 2-driven asthmatic responses. Immunology.

[165] Prietl B., Pilz S., Wolf M., Tomaschitz A., Obermayer-Pietsch B., Graninger W., Pieber T.R. (2010). Vitamin D Supplementation and Regulatory T Cells in Apparently Healthy Subject: Vitamin D Treatment for Autoimmune Diseases. IMAJ.

[166] Smolders J., Peelen E., Thewissen M., Tervaert J.W.C., Menheere P., Hupperts R., Dmoiseaux J. (2010). Safety and T Cell Modulating Effects of High Dose Vitamin D3 Supplementation in Multiple Sclerosis. PLoS ONE.

[167] Zold E., Szodoray P., Kappelmayer J., Gaal J., Csathy L., Barath S., Gyimesi E., Hajas A., Zeher M., Szegedi G. (2010). Impaired regulatory T-cell homeostasis due to vitamin D deficiency in undifferentiated connective tissue disease. Scand. J. Rheumatol..

[168] Zold E., Szodoray P., Nakken B., Barath S., Kappelmayer J., Csathy L., Hajas A., Sipka S., Gyimesi E., Gaal J. (2011). Alfacalcidol treatment restores derailed immune-regulation in patients with undifferentiated connective tissue disease. Autoimmun. Rev..

[169] Bobryshev Y. (2010). Vitamin D3 Suppresses Immune Reactions in Atherosclerosis, Affecting Regulatory T Cells and Dendritic Cell Function. Arterioscler. Thromb. Vasc. Biol..

[170] Van der Aar D.A.M., Sibiryak D.S., Bakdash G., van Capel T.M.M., van der Kleij H.P.M., Opstelten J.E., Teunissen M.B.M., Kapsenberg M.L., de Jong E.C. (2011). Vitamin D3 targets epidermal and dermal dendritic cells for induction of distinct regulatory T cells. J. Allergy Clin. Immunol..

[171] Zold E., Barta Z., Bodolay E. (2011). Vitamin D Deficiency and Connective Tissue Disease. Vitam. Horm..

[172] Takeda M., Yamashita T., Sasaki N., Nakajima K., Kita T., Shinohara M. (2010). Oral Administration of an Active Form of Vitamin D3 (Calcitriol) Decreases Atherosclerosis in Mice by Inducing Regulatory T Cells and Immature Dendritic Cells with Tolerogenic Functions. Arterioscler. Thromb. Vasc. Biol..

[173] Chambers E.S., Hawrylowicz C.M. (2011). The impact of vitamin D on regulatory T cells. Curr. Allergy Asthma Rep..

[174] Bock G., Prietl B., Mader J.K., Holler E., Wolf M., Pilz S., Graninger W.B., Obermayer-pietsch B.M., Pieber T.R. (2011). The effect of vitamin D supplementation on peripheral regulatory T cells and B cell function in healthy humans: A randomized controlled trial. Diabetes Metab. Res. Rev..

[175] Kilick J., Hay J., Morandi E., Vermeren S., Kari S., Angles T., Williams A., Damoiseaux J., Astier A.L. (2020). Vitamin D/CD46 Crosstalk in Human T Cells in Multiple Sclerosis. Front. Immunol..

[176] Cantorna M. (2012). Vitamin D, Multiple Sclerosis and Inflammatory Bowel Disease. Arch. Biochem. Biophys..

[177] Urry Z., Chambers E.S., Xystrakis E., Dimeloe S., Richards D.F., Gabrysova L., Christensen J., Gupta A., Saglani S., Bush A. (2012). The role of 1alpha,25-dihydroxyvitamin D3 and cytokines in the promotion of distinct Foxp3+ and IL-10+ CD4+ T cells. Eur. J. Immunol..

[178] Ooi J.H., Chen J., Cantorna M.T. (2012). Vitamin D regulation of immune function in the gut: Why do T cells have vitamin D receptors?. Mol. Asp. Med..

[179] Jeffery L.E., Wood A.M., Qureshi O.S. (2012). Availability of 25-hydroxyvitamin D3 to antigen presenting cells controls the balance between regulatory and inflammatory T cell responses. J. Immunol..

[180] Farias A.S., Spagnol G.S., Bordeaux-Rego P., Oliveira C.O.F., Fontana A.G.M., de Paula R.F.O., Santos M.P.A., Pradella F., Moraes A.S., Oliveira E.C. (2013). Vitamin D3 Induces IDO+ Tolerogenic DCs and Enhances Treg, Reducing the Severity of EAE. CNS Neurosci. Ther..

[181] Baráth S., Nagy G., Zöld E., Csípo I., Gyimesi E., Zeher M., Bodolay E. (2013). Conductor of regulatory cells: Does vitamin D restore the shifted balance of the distinct regulatory cell types in undifferentiated connective tissue disease?. Immunol. Lett..

[182] Kongsbak M., Levring T.B., Geisler C., von Essen M.R. (2013). The vitamin D receptor and T cell function. Front. Immunol..

[183] Prietl B., Treiber G., Pieber T.R., Amrein K. (2013). Vitamin D and Immune Function. Nutrients.

[184] Gupta A., Dimeloe S., Richards D.F., Chambers E.S., Black C., Urry Z., Ryanna K., Xystrakis E., Bush A., Saglani S. (2014). Defective IL-10 expression and in vitro steroid-induced IL-17A in paediatric severe therapy-resistant asthma. Thorax.

[185] Chambers E.S., Suwannasaen D., Mann E.H., Urry Z., Richards D.F., Lertmemongkolchai G., Hawrylowicz C.M. (2014). 1a,25-dihydroxyvitamin D3 in combination with transforming growth factor-b increases the frequency of Foxp3+ regulatory T cells through preferential expansion and usage of interleukin-2. Immunology.

[186] Cantorna M., Waddell A. (2014). The vitamin D receptor turns off chronically activated T cells. Ann. N. Y. Acad. Sci..

[187] Prietl B., Treiber G., Mader J.K., Hoeller E., Wolf M., Pilz S., Graninger W.B., Obermayer-Pietsch B.M., Pieber T.R. (2014). High-dose cholecalciferol supplementation significantly increases peripheral CD4^+^ Tregs in healthy adults without negatively affecting the frequency of other immune cells. Eur. J. Nutr..

[188] Keating P., Munim A., Hartmann J.X. (2014). Effect of vitamin D on T-helper type 9 polarized human memory cells in chronic persistent asthma. Ann. Allergy Asthma Immunol..

[189] Van Belle T.L., Vanherwegen A.S., Feyaerts D., De Clercq P., Verstuyf A., Korf H., Gysemans C., Mathieu C. (2014). 1,25-Dihydroxyvitamin D3 and Its Analog TX527 Promote a Stable Regulatory T Cell Phenotype in T Cells from Type 1 Diabetes Patients. PLoS ONE.

[190] Cantorna M., Snyder L., Lin Y., Yang L. (2015). Vitamin D and 1,25(OH)2D Regulation of T cells. Nutrients.

[191] Goldsmith J. (2015). Vitamin D as an Immunomodulator: Risks with Deficiencies and Benefits of Supplementation. Healthcare.

[192] Hayes C.E., Hubler S.L., Moore J.R. (2015). Vitamin D actions on CD4CT cells in autoimmune disease. Front. Immunol. TCell Biol..

[193] Bizzaro G., Shoenfeld Y. (2015). Vitamin D: A panacea for autoimmune diseases?. Can. J. Physiol. Pharmacol..

[194] Ghoryania M., Sahebarib M., Mahmoudia M., Abdollahib N., Reihania H., Rabe S.Z.T., Tabasi N., Rastin M. (2016). Immunomodulatory vitamin D effects on regulatory T-cells and cytokines in an in vitro study on patients with systemic lupus erythematosus. Food Agric. Immunol..

[195] Konijeti G.G., Arora P., Boylan M.R., Song Y., Huang S., Harrell F., Newton-Cheh C., O’Neill D., Korzenik J., Wang T.J. (2016). Vitamin D Supplementation Modulates T Cell–Mediated Immunity in Humans: Results from a Randomized Control Trial. J. Clin. Endocrinol. Metab..

[196] Gorman S., Geldenhuys S., Judge M., Weeden C.E., Waithman J., Hart P.H. (2016). Dietary Vitamin D Increases Percentages and Function of Regulatory T Cells in the Skin-Draining Lymph Nodes and Suppresses Dermal Inflammation. J. Immunol. Res..

[197] Muris A.H., Smolders J., Rolf L., Thewissen M., Hupperts R., Damoiseaux J. (2016). Immune regulatory effects of high dose vitamin D3 supplementation in a randomized controlled trial in relapsing remitting multiple sclerosis patients receiving IFNbeta; the SOLARIUM study. J. Neuroimmunol..

[198] Fawaz L., Mrad M.F., Kazan J.M., Sayagh S., Akika R., Khoury S.J. (2016). Comparative effect of 25(OH)D3 and 1,25(OH)2D3 on Th17 cell differentiation. Clin. Immunol..

[199] Şıklar Z., Karataş D., Doğu F., Hacıhamdioğlu B., İkincioğulları A., Berberoğlu M. (2016). Regulatory T Cells and Vitamin D Status in Children with Chronic Autoimmune Thyroiditis. J. Clin. Res. Pediatr. Endocrinol..

[200] Dimitrov V., White J. (2017). Vitamin D signaling in intestinal innate immunity and homeostasis. Mol. Cell. Endocrinol..

[201] Zhoua Q., Qinb S., Zhanga J., Zhona L., Pena Z., Xinga T. (2017). 1,25(OH)2D3 induces regulatory T cell differentiation by influencing the VDR/PLC-gamma1/TGF-beta1/pathway. Mol. Immunol..

[202] Altieri B., Muscogiuri G., Barrea L., Mathieu C., Vallone C.V., Mascitelli L., Bizzaro G., Altieri V.M., Tirabassi G., Balercia G. (2017). Does vitamin D play a role in autoimmune endocrine disorders? A proof of concept. Rev. Endocr. Metab. Disord..

[203] Bizzaro G., Antico A., Fortunato A., Bizzaro N. (2017). Vitamin D and Autoimmune Diseases: Is vitamin D Receptor (vDR) polymorphism the Culprit?. IMAJ.

[204] Sassi F., Tamone C., D’Amelio P. (2018). Vitamin D: Nutrient, Hormone, and Immunomodulator. Nutrients.

[205] Gorman S., Geldenhuys S., Weeden C.E., Grimbaldeston M.A., Hart P.H. (2018). Investigating the roles of regulatory T cells, mast cells and interleukin-9 in the control of skin inflammation by vitamin D. Arch. Dermatol. Res..

[206] Damoiseaux J., Smolders J. (2018). The Engagement Between Vitamin D and the Immune System: Is Consolidation by a Marriage to Be Expected?. EBioMedicine.

[207] Vanherwegen A., Eelenb G., Ferreira G.B., Ghesquiere B., Cook D.P., Nikolic T., Roep B., Carmeliet P., Telang S., Mathieu C. (2019). Vitamin D controls the capacity of human dendritic cells to induce functional regulatory T cells by regulation of glucose metabolism. J. Steroid Biochem. Mol. Biol..

[208] Heier I., Soyland E., Krogstad A., Rodriguez-Gallego C., Nenseter M.S., Jahnsen F.L. (2011). Sun exposure rapidly reduces plasmacytoid dendritic cells and inflammatory dermal dendritic cells in psoriatic skin. Br. J. Dermatol..

[209] Soyland E., Heier I., Rodriguez-Gallego C., Molines T.E., Johansen F.-E., Holven K.B., Halvorsen B., Aukrust P., Jahnsen F.L., de la Rosa Carrillo D. (2011). Sun exposure induces rapid immunological changes in skin and peripheral blood in patients with psoriasis. Br. J. Dermatol..

[210] Khoo A.L., Koenen H.J., Chai L.Y., Sweep F.C., Netea M.G., van der Ven A.J., Joosten I. (2012). Seasonal Variation in Vitamin D3 Levels Is Paralleled by Changes in the Peripheral Blood Human T Cell Compartment. PLoS ONE.

[211] Schwarz A., Maeda A., Wild M.K., Kernebeck K., Gross N., Aragane Y., Beissert S., Vestweber D., Schwarz T. (2004). Ultraviolet Radiation-Induced Regulatory T Cells Not Only Inhibit the Induction but Can Suppress the Effector Phase of Contact Hypersensitivity. J. Immunol..

[212] Schwarz T. (2005). Regulatory T cells induced by ultraviolet radiation. Int. Arch. Allergy Immunol..

[213] Schwarz T. (2008). 25 years of UV-induced Immunosuppression Mediated by T Cells—From Disregarded T Suppressor Cells to Highly Respected Regulatory T Cells. Photochem. Photobiol..

[214] Shintani Y., Yasuda Y., Kobayashi K., Maeda A., Morita A. (2008). Narrowband ultraviolet B radiation suppresses contact hypersensitivity. Photoderm. Photoimmunol. Photomed..

[215] Loser K., Beissert S. (2009). Regulation of cutaneous immunity by the environment: An important role for UV irradiation and vitamin D. Int. Immunopharmacol..

[216] McGlade J.P., Strickland D.H., Lambert M.J., Gorman S., Thomas J.A., Judge M.A., Burchell J.T., Zosky G.R., Hart P.H. (2010). UV inhibits allergic airways disease in mice by reducing effector CD4 T cells. Clin. Exp. Allergy.

[217] Gorman S., McGlade J.P., Lambert M.J.M., Strickland D.H., Thomas J.A., Hart P.H. (2010). UV exposure and protection against allergic airways disease. Photochem. Photobiol. Sci..

[218] Racz E., Prens E.P., Kurek D., Kant M., de Ridder D., Mourits S., Barveldt E.M., Ozgur Z., van IJcken R.F.J., Laman J.D. (2011). Effective treatment of psoriasis with narrowband UVB phototherapy is linked to suppression of the IFN and Th17 pathways. J. Ibvest. Dermatol..

[219] Milliken S.V.I., Wassall H., Lewis B.J., Logie J., Barker R.N., Macdonald H., Vickers M.A., Ormerod A.D. (2012). Effects of ultraviolet light on human serum 25-hydroxyvitamin D and systemic immune function. J. Allergy Clin. Immunol..

[220] Furuhashi T., Saito C., Torii K., Nishida E., Yamazaki S., Morita A. (2013). Photo(chemo)therapy Reduces Circulating TH17 Cells and Restores Circulating Regulatory T Cells in Psoriasis. PLoS ONE.

[221] Yamazaki S., Nishioka A., Kasuya S., Ohkura N., Hemmi H., Kaisho T., Taguchi O., Sakaguchi S., Morita A. (2014). Homeostasis of Thymus-Derived Foxp3+ Regulatory T Cells Is Controlled by Ultraviolet B Exposure in the Skin. J. Immunol..

[222] Breuer J., Schwab N., Schneider-Hohendorf T., Marziniak M., Mohan H., Bhatia U., Gross C.C., Clausen B.E., Weishaupt C., Luger T.A. (2014). Ultraviolet B light attenuates the systemic immune response in central nervous system autoimmunity. Ann. Neurol..

[223] Wang X., Wang G., Gong Y., Liu Y., Gu J., Chen W., Shi Y. (2015). Disruption of circulating CD4+ T-lymphocyte subpopulations in psoriasis patients is ameliorated by narrow-band UVB therapy. Cell Biochem. Biophys..

[224] Batycka-Baran A., Besgen P., Wolf R., Szepietowski J.C., Prinz J.C. (2016). The effect of phototherapy on systemic inflammatory process in patients with plaque psoriasis. J. Photochem. Photobiol. B Biol..

[225] Bialecka M., Ostasz R., Kurzawski M., Klimowicz A., Fabianczyk H., Bojko P., Dziedziejko V., Safranow K., Machoy-Mokrzynska A., Drozdzik M. (2016). IL17A and IL17F gene polymorphism association with psoriasis risk and response to treatment in a polish population. Dermatology.

[226] Gui J., Gober M., Yang X., Katlinski K.V., Marshall C.M., Sharma M., Werth V.P., Baker D.P., Rui H., Seykora J.T. (2016). Therapeutic elimination of the type 1 interferon receptor for treating psoriatic skin inflammation. J. Investig. Dermatol..

[227] Zhang D., Chen Y., Chen L., Yang R., Wang L., Liu W., Zhai Z., Shen Z. (2016). Ultraviolet irradiation promotes FOXP3 transcription via p53 in psoriasis. Exp. Dermatol..

[228] Bora S., Cantorna M.T. (2017). The role of UVR and vitamin D on T cells and inflammatory bowel disease. Photochem. Photobiol. Sci..

[229] Racz E., Prens E.P. (2017). Phototherapy of Psoriasis, a Chronic Inflammatory Skin Disease. Adv. Exp. Med. Biol..

[230] Yamazaki S., Odanaka M., Nishioka A., Kasuya S., Shime H., Hemmi H., Imai M., Riethmacher D., Kaisho T., Ohkura N. (2018). Ultraviolet B Induced Maturation of CD11b-Type Langerin- Dendritic Cells Controls the Expansion of Foxp3 + Regulatory T Cells in the Skin. J. Immunol..

[231] Kotb I.S., Lewis B.J., Barker R.N., Ormerod A.D. (2018). Differential effects of phototherapy, adalimumab and betamethasone–calcipotriol on effector and regulatory T cells in psoriasis. Br. J. Dermatol..

[232] Zhang P., Wu M.X. (2018). A clinical review of phototherapy for psoriasis. Lasers Med. Sci..

[233] Tan S.Y., Buzney E., Mostaghimi A. (2018). Trends in phototherapy utilization among Medicare beneficiaries in the United States, 2000 to 2015. J. Am. Acad. Dermatol..

[234] Sakaguchi S., Sakaguchi N., Asano M., Itoh M., Toda M. (1995). Immunologic Self-Tolerance Maintained by Activated T Cells Expressing IL-2 Receptor Alpha-Chains (CD25). J. Immunol..

[235] Groux H., O’Garra A., Bigler M., Rouleau M., Antonenko S., de Vries J.E., Roncarolo M. (1997). ACD4+ T-cell subset inhibits antigen-specific T-cell responses and prevents colitis. Nature.

[236] Groux H., O’Garra A., Bigler M., Rouleau M., Antonenko S., de Vries J.E., Roncarolo M.G. (1997). Interleukin 10 is a growth factor for a population of regulatory T cells. Nature.

[237] Dejaco C., Duftner C., Grubeck- Loebenstein B., Schirmer M. (2005). Imbalance of regulatory T cells in human autoimmune diseases. Immunology.

[238] Sakaguchi S., Ono M., Setoguchi R., Yagi H., Hori S., Fehervari Z., Shimizu J., Takahashi T., Nomura T. (2006). Foxp3+ CD25+ CD4+ natural regulatory T cells in dominant self-tolerance and autoimmune disease. Immunol. Rev..

[239] Tang Q., Bluestone J.A. (2008). The Foxp3+ regulatory T cell: A jack of all trades, master of regulation. Nat. Immunol..

[240] Anderton S., Liblau R. (2008). Regulatory T cells in the control of inflammatory demyelinating diseases of the central nervous system. Curr. Opin. Neurol..

[241] Robinson D.S. (2009). Regulatory T Cells and asthma. Clin. Exp. Allergy..

[242] Corthay A. (2009). How do Regulatory T Cells Work?. Scand. J. Immunol..

[243] Buckner J.H. (2010). Mechanisms of impaired regulation by CD4^+^CD25^+^FOXP3^+^ regulatory T cells in human autoimmune diseases. Nat Rev Immunol..

[244] Shevach E. (2011). The Resurrection of T Cell-Mediated Suppression. J. Immunol..

[245] Loser K., Beissert S. (2012). Regulatory T Cells: Banned Cells for Decades. J. Investig. Dermatol..

[246] Matozzi C., Salvi M., D’Epiro S., Giancristoforo S., Macaluso L., Luci C., Lal K., Calvieri S., Richetta A.G. (2013). Importance of Regulatory T Cells in the Pathogenesis of Psoriasis: Review of the Literature. Dermatology.

[247] Fessler J., Ficjan A., Duftner C., Dejaco C. (2013). The impact of aging on regulatoryT-cells. Front. Immunol..

[248] Fessler J., Felber A., Duftner C., Dejaco C. (2013). Therapeutic potential of regulatory T cells in autoimmune disorders. BioDrugs.

[249] Kleinewietfeld M., Hafler D.A. (2013). The plasticity of human Treg and Th17 cells and its role in autoimmunity. Semin. Immunol..

[250] Ohkura N., Kitagawa Y., Sakaguchi S. (2013). Development and maintenance of regulatory T cells. Immunity.

[251] Galgani M., De Rosa V., La Cava A., Matarese G. (2016). Role of Metabolism in the Immunobiology of Regulatory T Cells. J. Immunol..

[252] Fessler J., Raicht A., Husic R., Ficjan A., Schwarz C., Duftner C., Schwinger W., Graninger W.B., Stradner M.H., Dejaco C. (2017). Novel senescent regulatory T-cell subset with impaired suppressive Function in rheumatoid arthritis. Front. Immunol..

[253] Kumar P., Saini S., Khan S., Lele S.S., Prabhakar B.S. (2019). Restoring Self-tolerance in Autoimmune Diseases by Enhancing Regulatory Tcells. Cell. Immunol..

[254] Okeke E.B., Uzonna J.E. (2019). The Pivotal Role of Regulatory T Cells in the Regulation of Innate Immune Cells. Front. Immunol..

[255] Haddad J.G., Chyu K.J. (1971). Competitive protein-binding radioassay for 25-hydroxycholecalciferol. J. Clin. Endocrinol..

[256] Hollis B.W., Wagner C.L., Drezner M.K., Binkley N.K. (2007). Circulating Vitamin D3 and 25-hydroxyvitamin D in Humans: An Important Tool to Define Adequate Nutritional Vitamin D Status. J. Steroid Biochem. Mol. Biol..

[257] Luxwolda M.F., Kuipers R.S., Kema I.P., Janneke Dijck-Brouwer D.A., Muskiet F.A.J. (2012). Traditionally living populations in East Africa have a mean serum 25-hydroxyvitamin D concentration of 115 nmol/L. Br. J. Nutr..

[258] Holick M. (2007). Vitamin D Deficiency. N. Engl. J. Med..

[259] Tsuprykov O., Elitok S., Buse C., Chu C., Krämer B.K., Hocher B. (2021). Opposite correlation of 25-hydroxyvitamin D and 1,25-dihydroxyvitamin D metabolites with gestational age, bone- and lipid-biomarkers in pregnant women. Sci. Rep..

[260] Shufei Zeng S., Chu C., Doebis C., von Baehr V., Hocher B. (2021). Reference values for free 25-hydroxy-vitamin D based on established total 25-hydroxy-vitamin D reference values. J. Steroid Biochem. Mol. Biol..

[261] Garrett-Mayer E., Wagner C.L., Hollis B.W., Kindy M.S., Gattoni-Celli S. (2012). Vitamin D3 supplementation (4000 IU/d for 1 y) eliminates differences in circulating 25-hydorxyvitamin D between African American and white men. Am. J. Clin. Nutr..

[262] Marshall D.T., Savage S.J., Garrett-Mayer E.G., Keane T.E., Hollis B.W., Horst R.L., Ambrose L.H., Kindy M.S., Gattoni-Celli S. (2012). Vitamin D3 supplementation at 4000 international units per day for one year results in a decrease of positive cores at repeat biopsy in subjects with low-risk prostate Cancer under active surveillance. J. Clin. Endocrinol. Metab..

[263] Dudenkov D.V., Yawn B.P., Oberelman S.S., Fischer P.R., Singh R.J., Cha S.S., Maxson J.A., Quigg S.M., Thacher T.D. (2015). Changing incidence of serum 25-Hydroxyvitamin d values above 50 ng/mL: A 10-Year population-based study. Mayo Clin. Proc..

[264] Kimball S.M., Mirhosseini N., Holick M.F. (2017). Evaluation of vitamin D3 intakes up to 15,000 international units/day and serum 25-hydroxyvitamin D concentrations up to 300 nmol/L on calcium metabolism in a community setting. Derm. Endocrinol..

[265] Heaney R.P., Davies K.M., Chen T.C., Holick M.F., Barger-Lux M.J. (2016). Human serum 25-hydroxycholecalciferol response to extended oral dosing with cholecalciferol. Am. J. Clin. Nutr..

[266] Christiansen C., Rodbro P., Sjö O. (1974). “Anticonvulsant action” of vitamin D in epileptic patients. A controlled pilot study. Br. Med. J..

[267] Garland C., Garland F. (1980). Do sunlight and vitamin D reduce the likelihood of colon cancer?. Int. J. Epidemiol..

[268] Shabahang M., Buras R.R., Davoodi F., Schumaker L.M., Nauta R.J., Evans S.R. (1993). 1,25-Dihydroxyvitamin D3 receptor as a marker of human colon carcinoma cell line differentiation and growth inhibition. Cancer Res..

[269] Shabahang M., Buras R.R., Davoodi F., Schumaker L.M., Nauta R.J., Uskokovic M.R., Brenner R.V., Evans S.R. (1994). Growth inhibition of HT-29 human colon cancer cells by analogues of 1,25-dihydroxyvitamin D3. Cancer Res..

[270] Shabahang M., Buffan A.E., Nolla J.M., Schumaker L.M., Brenner R.V., Buras R.R., Nauta R.J., Evans S.R. (1996). The effect of 1, 25-dihydroxyvitamin D3 on the growth of soft-tissue sarcoma cells as mediated by the vitamin D receptor. Ann. Surg. Oncol..

[271] Evans S.R., Houghton A.M., Schumaker L., Brenner R.V., Buras R.R., Davoodi F., Nauta R.J., Shabahang M. (1996). Vitamin D receptor and growth inhibition by 1,25-dihydroxyvitaminD3 in human malignant melanoma cell lines. J. Surg. Res..

[272] Derex L., Trouillas P. (1997). Reversible parkinsonism, hypophosphoremia and hypocalcemia under Vitamin D therapy. Case Rep. Mov. Disord..

[273] Koutkia P., Lu Z., Chen T.C., Holick M.F. (2001). Treatment of vitamin D deficiency due to Crohn’s disease with tanning bed ultraviolet B radiation. Gastroenterology.

[274] Bischoff-Ferrari H., Dawson-Hughes B., Willett W., Stachelin H., Bazemore M., Zee R., Wong J. (2004). Effect of vitamin D on falls. A meta-analysis. JAMA.

[275] Wang T.T., Nestel F.P., Bourdeau V., Nagai Y., Wang Q., Liao J., Tavera-Mendoza L., Lin R., Hanrahan J.W., Mader S. (2004). Cutting Edge: 1,25-Dihydroxyvitamin D3 Is a direct inducer of Antimicrobial Peptide gene expression. J. Immunol..

[276] Gombert A., Borregaard N., Koeffler H.P. (2005). Human cathelicidin antimicrobial peptide (CAMP) gene is a direct target of the vitamin D receptor and is strongly up-regulated in myeloid cells by 1, 25-dihydroxyvitamin D3. FASEB J..

[277] Bischoff-Ferrari H., Willett W., Wong J., Giovannucci E., Dietrich T., Dawson-Hughes B. (2005). Fracture prevention with Vitamin D supplementation. A meta-analysis of randomized controlled trials. JAMA.

[278] Giovannucci E. (2005). The epidemiology of vitamin D and cancer incidence and mortality: A review (United States). Cancer Causes Control.

[279] Bischoff-Ferrari H., Orav E., Dawson-Hughes B. (2006). Effect of cholecalciferol plus calcium on falling in ambulatory older men and women. A 3-year randomized controlled trial. Arch. Intern. Med..

[280] Cannell J.J., Vieth R., Umhau J.C., Holick M.F., Grant W.B., Madronich S., Garland C.F., Giovannucci E. (2006). Epidemic influenza and vitamin D. Epidemiol. Infect..

[281] Garland C.F., Garland F.C., Gorham E.D., Lipkin M., Newmark H., Mohr S.B., Holick M.F. (2006). The role of vitamin d in Cancer prevention. Am. J. Public Health.

[282] Liu P.T., Stenger S., Li H., Wenzel L., Tan B.H., Krutzik S.R., Ochoa M.T., Schauber J., Wu K., Meinken C. (2006). Toll-Like Receptor Triggering of a Vitamin D-Mediated Human Antimicrobial Response. Science.

[283] Wicherts I., van Schoor N., Boeke A., Visser M., Deeg D., Smit J., Knol D., Lips P. (2007). Vitamin D status predicts physical performance and its decline in older persons. J. Clin. Endocrinol. Metab..

[284] Lappe J.M., Travers-Gustafson D., Davies K.M., Recker R.R., Heaney R.P. (2007). Vitamin D and calcium supplementation reduces cancer risk: Results of a randomized trial. Am. J. Clin. Nutr..

[285] Newmark H.L., Newmark J. (2007). Vitamin d and Parkinson’s disease—A hypothesis. Mov. Disord..

[286] Lee P., Chen R. (2008). Vitamin D as an analgesic for patients with type 2 diabetes and neuropathic pain. Arch. Int. Med..

[287] Fleet J.C. (2008). Molecular actions of vitamin D contributing to cancer prevention. Mol. Aspects Med..

[288] Cannell J.J., Zasloff M., Garland C.F., Scragg R., Giovannucci E. (2008). On the epidemiology of influenza. Virol. J..

[289] Gombart A. (2009). The vitamin D-antimicrobial peptide pathway and its role in protection against infection. Future Microbiol..

[290] Al-Said Y., Al-Rached H., Al-Qahtani H., Jan M. (2009). Severe proximal myopathy with remarkable recovery after Vitamin D treatment. Can. J. Neurol. Sci..

[291] Smolders J., Menheere P., Thewissen M., Peelen E., Cohen J.W., Tervaert R., Hupperts R. (2009). Vitamin D status is positively correlated with regulatory T cell function in patients with multiple sclerosis. PLoS ONE.

[292] Fernandes de Abreu D.A., Eyles D., Feron F. (2009). Vitamin D, a neuro-immunomodulator: Implications for neurodegenerative and autoimmune diseases. Psychoneuroendocrinology.

[293] Urashima M., Segawa T., Okazaki M., Kurihara M., Wada Y., Ida H. (2010). Randomized trial of vitamin D supplementation to prevent seasonal influenza A in schoolchildren. Am. J. Clin. Nutr..

[294] Uhmann A., Niemann H., Lammering B., Henkel C., Heb I., Rosenberger A., Dukkin C., Schraepler A., Schulz-Schaeffer W., Hahn H. (2011). Antitumoral effects of calcitriol in basal cell carcinomas involve inhibition of hedgehog signaling and induction of vitamin d receptor signaling and differentiation. Mol. Cancer Ther..

[295] Garland C.F., French C.B., Baggerly L.L., Heaney R.P. (2011). Vitamin D supplement doses and serum 25-hydroxyvitaminD in the range associated with cancer prevention. Anticancer. Res..

[296] Osunkwo I. (2011). Complete resolution of sickle cell chronic pain with high dose vitamin D therapy: A case report and review of the literature. J Pediatr. Hematol. Oncol..

[297] Osunkwo I., Ziegler T.R., Alvarez J., McCracken C., Cherry K., Osunkwo C.E., Ofori-Acquah S.F., Ghosh S., Ogunbobode A., Rhodes J. (2012). High Dose Vitamin D Therapy for Chronic Pain in Children and Adolescents with Sickle Cell Disease: Results of a Randomized Double Blind Pilot Study. Br. J. Haematol..

[298] Fleet J.C., Desmet M., Johnson R. (2012). Vitamin D and cancer: A review of molecular mechanisms. Biochem. J..

[299] Holló A., Clemens Z., Kamondi A., Lakatos P., Szűcs A. (2012). Correction of vitamin D deficiency improves seizure control in epilepsy: A pilot study. Epilepsy Behav..

[300] Hossein-nezhad A., Holick M.F. (2013). Vitamin D for health; a global perspective. Mayo Clin. Proc..

[301] Pludowski P., Holick M.F., Pilz S., Wagner C.L., Hollis B.W., Grant W.B., Shoenfeld Y., Lerchbaum E., Llewellyn D.J., Kienreich K. (2013). Vitamin D effects on musculoskeletal health, immunity, autoimmunity, cardiovascular disease, cancer, fertility, pregnancy, dementia and mortality-a review of recent evidence. Autoimmun. Rev..

[302] Khare D., Godbole N.M., Pawar S.D., Mohan V., Pandey G., Gupta S., Kumar D., Dhole T.N., Godbole M.M. (2013). Calcitriol [1, 25[OH]2 D3] pre- and post-treatment suppresses inflammatory response to influenza A (H1N1) infection in human lung A549 epithelial cells. Eur. J. Nutr..

[303] Roy S., Sherman A., Monari-Sparks M.J., Schweiker O., Hunter K. (2014). Correction of Low Vitamin D Improves Fatigue: Effect of Correction of Low Vitamin D in Fatigue Study (EViDiF Study). N. Am. J. Med. Sci..

[304] Castro M., King T.S., Kunselman S.J., Cabana M.D., Denlinger L., Holguin F., Kazani S.D., Moore W.C., Moy J., Sorkness C.A. (2014). Effect of Vitamin D3 on Asthma Treatment Failures in Adults with Symptomatic Asthma and Lower Vitamin D Levels. The VIDA Randomized Clinical Trial. JAMA.

[305] Nguyen S., Baggerly L., French C., Heaney R.P., Gorham E.D., Garland C.F. (2014). 25Hydroxyvitamin D in the range of 20 to 100 ng/mL and Incidence of Kidney Stones. Am. J. Public Health.

[306] Baggerly C.A., Cuomo R.E., French C.B., Garland C.F., Gorham E.D., Grant W.B., Heaney R.P., Holick M.F., Hollis B.W., McDonnell S.L. (2015). Sunlight and Vitamin D: Necessary for Public Health. J. Am. Coll. Nutr..

[307] Holick M.F. (2015). Vitamin D is not as toxic as was once thought: A historical and an up-to date perspective. Mayo Clin. Proc..

[308] Khayznikov M., Hemachrandra K., Pandit R., Kumar A., Wang P., Glueck C.J. (2015). Statin Intolerance Because of Myalgia, Myositis, Myopathy, or Myonecrosis Can in Most Cases be Safely Resolved by Vitamin D Supplementation. N. Am. J. Med. Sci..

[309] Jetty V., Glueck C.J., Wang P., Shah P., Prince M., Lee K., Goldenberg M., Kumar A. (2016). Safety of 50,000–100,000 Units of Vitamin D3/Week in Vitamin D-Deficient, Hypercholesterolemic Patients with Reversible Statin Intolerance. N. Am. J. Med. Sci..

[310] Cannon T.L., Ford J., Hester D., Trump D.L. (2016). The incidental use of high-dose vitamin D3 in pancreatic Cancer. Case Rep. Pancreat. Cancer.

[311] McDonnell S.L., Baggerly K.A., Baggerly C.A., Aliano J.L., French C.B., Baggerly L.L., Ebeling M.D., Rittenberg C.S., Goodier C.G., Mateus Nino J.F. (2017). Maternal 25(OH)D concentrations ≥ 40 ng/mL associated with 60% lower preterm birth risk among general obstetrical patients at an urban medical center. PLoS ONE.

[312] Martineau A.R., Jolliffe D.A., Hooper R.L., Greenberg L., Aloia J.F., Bergman P., Dubnov-Raz G., Esposito S., Ganmaa D., Ginde A.A. (2017). Vitamin D supplementation to prevent acute respiratory tract infections: Systematic review and meta-analysis of individual participant data. BMJ.

[313] Madden J.M., Murphy L., Zgaga L., Bennett K. (2018). De novo vitamin D supplement use post-diagnosis is associated with breast cancer survival. Breast Cancer Res. Treat..

[314] McDonnell S.L., Baggerly C.A., French C.B., Baggerly L.L., Garland C.F., Gorham E.D., Hollis B.W., Trump D.L., Lappe J.M. (2018). Breast cancer risk markedly lower with serum 25-hydroxyvitamin D concentrations>60 vs. <20ng/mL (150 vs. 50 nmol/L): Pooled analysis of two randomized trials and a prospective cohort. PLoS ONE.

[315] Moretti R., Morelli M.E., Caruso P. (2018). Vitamin D in Neurological Diseases: A Rationale for a Pathogenic Impact. Int. J. Mol. Sci..

[316] Dudenkov D.V., Mara K.C., Petterson T.M., Maxson J.A., Thacher T.D. (2018). Serum 25-Hydroxyvitamin d values and risk of all cause and cause-specific mortality: A population-based cohort study. Mayo Clin. Proc..

[317] Manson J.E., Cook N.R., Lee I.M., Christen W., Bassuk S.S., Mora S., Gibson H., Gordon D., Copeland T., D’Agostino D. (2019). Vitamin D Supplements and Prevention of Cancer and Cardiovascular Disease. N. Engl. J. Med..

[318] Forno E., Bacharier L.B., Phipatanakul W., Guilbert T.W., Cabana M.D., Ross K., Covar R., Gern J.E., Rosser F.J., Blatter J. (2020). Effect of Vitamin D3 Supplementation on Severe Asthma Exacerbations in Children with Asthma and Low Vitamin D Levels. The VDKA Randomized Clinical Trial. JAMA.

[319] https://www.worldometers.info/coronavirus/.

[320] Leake C. (1936). Vitamin D Toxicity. Calif. West. Med..

[321] Bevans M., Taylor H. (1947). Lesions Following the use of Ertron in Rheumatoid Arthritis. Am. J. Pathol..

[322] Chen X., Chu C., Doebis C., von Baehr V., Hocher B. (2021). Sex Dependent Association of Vitamin D with Insulin Resistance. J. Clin. Endocrinol. Metab..

[323] Kalb R.E., Fiorentino D.F., Lebwohl M.G., Toole J., Poulin Y., Cohen A.D., Goyal K., Fakharzadeh S., Calabro S., Chevrier M. (2015). Risk of Serious Infection with Biologic and Systemic Treatment of Psoriasis. Results from the Psoriasis Longitudinal Assessment and Registry (PSOLAR). JAMA Dermatol..

[324] Naderi H.R., Sheybani F., Pajand S.R. (2016). How Should We Manage Latent Tuberculosis Infection in Patients Receiving Anti-TNF-alpha Drugs: Literature Review. Iran Red Crescent Med. J..

[325] Dobry A.S., Quesenberry C.P., Ray G.T., Geier J.L., Asgari M.M. (2017). Serious infections among a large cohort of subjects with systemically treated psoriasis. J. Am. Acad. Dermatol..

[326] Fiorentino D., Ho V., Lebwohl M.G., Leite L., Hopkins L., Galindo C., Goyal K., Langhoff W., Fakharzadeh S., Srivastava B. (2017). Risk of malignancy with systemic psoriasis treatment in the Psoriasis Longitudinal Assessment Registry. J. Am. Acad. Dermatol..

[327] Kirkham B. Tumor Necrosis Factor-Alpha Inhibitors: An Overview of Adverse Effects. Up To Date. Literature Review Current through June 2019. This Topic Last Updated May 2018. https://www.uptodate.com/contents/tumor-necrosis-factor-alpha-inhibitors-an-overviewof-adverse-effects?search=tumor%20necrosis%20factor&source=search_result&selectedTitle=1~150&usage_type=default&display_rank=1.

[328] ISMP Quarter Watch Signals for Fingolimod and Infliximab. 5 April 2012. Data from 2011 Quarter 2. https://www.ismp.org/system/files/resources/2019-02/2011Q2.pdf.

[329] ISMP Quarter Watch Leading Drug Safety Issues of 2012. Three Anti-TNF Agents Dominate Manufacturer Reports of New, Serious Injuries. 17 October 2013. Data from 2012 Quarter 4 and Annual Report. https://www.ismp.org/system/files/resources/2019-02/2012Q4.pdf.

[330] ISMP Quarter Watch Annual Report Issue. Two Tumor Necrosis Factor Blockers Lead Overall Report Totals in 2014. 23 September 2015. Data from 2014 Quarters 3–4. https://www.ismp.org/system/files/resources/2019-02/2014Q4.pdf.

[331] ISMP Quarter Watch Cancer Risks of Biological Products for Psoriasis. 6 April 2016. Data from 2015 Quarter 3. https://pdfs.semanticscholar.org/d656/5182acc5e3eec881e32127291647783c4d6f.pdf.

[332] ISMP Quarter Watch Annual Report Issue. Direct Reports to the FDA: Adalimumab (HUMIRA), A Biological Product That Suppresses Tumor Necrosis Factor (TNF), Was the Leading Suspect Drug in Reports Submitted Directly to the FDA, rather than through Drug Manufacturers. 29 June 2016. Data from 2015 Q4. https://www.ismp.org/system/files/resources/2019-02/2015Q4.pdf.

[333] Walker-Journey J. Humira Generates Most Adverse Event Reports in FDA Database. http://www.rightinginjustice.com/news/2017/04/04/humira-generates-most-adverse-event-reports-in-fda-database/.

[334] ISMP Quarter Watch Complaints That a New Psoriasis Treatment Could Aggravate the Condition. 12 December 2018. Includes New Data from 2018 Q1–Q2. https://www.ismp.org/system/files/resources/2019-02/201812_0.pdf.

[335] Fauber J., Crowe K. Biologic Medications for Arthritis and Psoriasis Have Flooded the Market and Been Linked to 34,000 Deaths. *Milwaukee J. Sentinel*
**2019**. https://www.jsonline.com/story/news/investigations/2019/05/30/arthritis-psoriasis-drugs-darker-aspect-34-000-reports-deaths/1206103001/.

[336] U.S. Food and Drug Administration FDA Drug Safety Communication: Prescription Acetaminophen Products to Be Limited to 325 mg Per Dosage Unit; Boxed Warning Will Highlight Potential for Severe Liver Failure. https://www.fda.gov/drugs/drug-safety-and-availability/fda-drug-safety-communication-prescription-acetaminophen-products-be-limited-325-mg-dosage-unit.

[337] U.S. Food and Drug Administration Fda News Release. For Immediate Release: 13 January 2011. FDA Limits Acetaminophen in Prescription Combination Products; Requires Liver Toxicity Warnings. https://wayback.archive-it.org/7993/20170111224116/http:/www.fda.gov/NewsEvents/Newsroom/PressAnnouncements/ucm239894.htm.

