# Supplementary material for: Oral and Topical Vitamin D, Sunshine, and UVB Phototherapy Safely Control Psoriasis in Patients with Normal Pretreatment Serum 25-Hydroxyvitamin D Concentrations: A Literature Review and Discussion of Health Implications
Source: Nutrients. 2021 Apr 29;13(5):1511. doi: 10.3390/nu13051511 (PMC8146035; doi:10.3390/nu13051511)
Supplement: Supplementary file 1 [file nutrients-13-01511-s001.zip › nutrients-1144776-supplementary.pdf]

Review

Oral and Topical Vitamin D, Sunshine, and UVB Phototherapy Safely Control Psoriasis in Patients with Normal Pretreatment Serum 25-hydroxyvitamin D Concentrations: A Literature Review and Discussion of Health Implications

Patrick J McCullough[1,3*], William P McCullough[2], Douglas Lehrer[3] and Jeffrey B Travers[4] (ORCID 0000-0001-7232-1039), Steven J Repas

Supplemental Data Section

2. Oral vitamin D_2, oral 1 alpha-hydroxyvitaminD_3 ($1(OH)D_3$), oral calcitriol, topical calcitriol and oral vitamin D_3 safely treat psoriasis – 1930s to 2019

2.1. Sunshine and oral vitamin D_2 in the 1930s – Krafka [12]

In 1936, a report documented the use of oral vitamin D_2 to clear psoriasis plaques in three psoriasis patients, two of which were long-standing cases [12]. The author stated that the idea of using oral vitamin D for treating psoriasis came from the realization that "a commonly observed fact in the South concerning this disease is that it generally clears up to some extent in the summer sun." Knowing that vitamin D was made in the skin from the action of sunshine led to the hypothesis that psoriasis might respond to treatment with oral vitamin D_2 (Viosterol). Vitamin D became available commercially shortly after its discovery in the 1920s, allowing physicians to test this hypothesis. In this 1936 report, the first patient had a 10-year history of psoriasis. A description of the treatment used was given, but it is difficult to determine the exact dosages employed. However, the response noted was relatively quick and complete as the author noted that "Within sixty days from the beginning of the test, the skin of this patient was entirely clear."

The two other patients also responded very well. "The two cases were admirable for the test. The first was of only three weeks' duration; the second had run a course of thirty years." It appears that the patients were given a dose of 20,000 IU a day for 10 days, for a course or two, with a 10-day break in between, and then on a maintenance dose of 4000 IU a day. Both patients showed the same remarkable benefits as observed in the first patient.

The 1936 report closed with a discussion of the 3 cases: "Our experience with these three cases of psoriasis leads us to this preliminary report. We realize the shortcomings of such a limited experience in the light of what is now called "experimental research" that demands at least two hundred cases. If the treatment were at all hazardous or difficult, we would not presume to lay it before the profession. But the treatment is so simple that it should be put to a trial test in the interest of every patient suffering from this obnoxious condition. Certainly, it is worth a fair trial. We leave our results to be tested on a more elaborate scale by the larger clinics."

Other reports describing variable results from the use of vitamin D in treating psoriasis were also published in that era, but unfortunately the use of oral vitamin D to treat psoriasis and other diseases soon fell out of favor due to concerns of toxicity from hypercalcemia which was observed with the supraphysiogical doses of vitamin D then used [29-31, 33-39].

Published reports on the use of vitamin D in the treatment of psoriasis did not resume again until the 1980's, when a serendipitous observation was made in a psoriasis patient who was being treated with oral 1-hydroxyvitamin D_3 ($1(OH)D_3$) for osteoporosis, whose

skin showed remarkable clinical improvement [13]. This observation led to the resurrection of research into the use of vitamin D in treating psoriasis, which continues through today [12-28, 81-109].

2.2. Oral 1 alpha-hydroxyvitaminD$_3$, oral calcitriol and topical calcitriol in the 1980s – Morimoto [14]

The impetus for this study was the chance observation made the year before by two of the investigators when they were using 1(OH)D$_3$ as a treatment for osteoporosis in a patient who happened to have psoriasis, and whose skin cleared completely [13]. "This observation prompted us to confirm the effect by an open-design study" [14].

In the open-design study, both oral 1(OH)D$_3$, and oral and topically applied calcitriol were found to be safe and effective in clearing psoriasis skin lesions [14]. A total of 40 patients were studied: a) 17 with oral 1(OH)D$_3$, b) 4 with oral calcitriol, and c) 19 with topical calcitriol. This report is reviewed in the next 2 sections.

2.3. Oral 1 alpha-hydroxyvitaminD$_3$, 6-month study, 1986 – Morimoto [14]

A total of 17 patients with psoriasis vulgaris were treated orally with 1 µg/d of 1-OHD$_3$ for up to six months. Significant clinical improvement was observed in 13 (76%) by the end of the study period, with complete clearing of the skin lesions noted in 5 (29%). Patients were examined once every two weeks during the course of the study. Serum 25(OH)D, calcium, hepatic function, renal function, inorganic phosphate, parathyroid hormone, and plasma calcitonin concentrations were obtained at baseline and during follow-up visits. Data was presented as baseline versus 3-month levels. It took about 3 months to begin seeing significant clinical improvement in the 13 patients who responded well to treatment. In the remaining 4 patients, one showed slight improvement, 2 showed no change, and one deteriorated.

The baseline mean serum 25(OH)D concentration was 23 ± 12 ng/ml, and 27 ± 18 ng/ml at 3 months, which was not significantly different. It should be noted that 1(OH)D$_3$ is metabolized directly into calcitriol (1,25-dihydroxyvitamin D$_3$) and has no effect on serum 25(OH)D$_3$ concentrations. Mean serum calcium concentrations were 9.2 mg/dl at baseline and 9.6 mg/dl at 3 months. Mean serum calcitriol concentrations increased from 41 pg/ml at baseline to 62 pg/ml at three months. Although a statistically significant increase in serum calcium, phosphate, and calcitriol concentrations were observed after 3 months of treatment, "the increases were slight and remained within the normal range." It should be noted that no cases of hypercalcemia were observed, and no adverse side effects were noted in any patients. Baseline and on treatment lab values are shown in Table 1.

2.4. Oral calcitriol 0.5 µg/day and topical calcitriol, 1986 – Morimoto [14]

One of 4 patients (25%) treated with oral calcitriol showed significant clinical improvement in psoriasis. In the topical calcitriol group 16 of 19 (84%) showed significant clinical improvement within 3 to 4 weeks, with complete clearing noted in 3 (16%). The results with topical calcitriol are very similar to those seen in the patients treated with oral 1(OH)D$_3$ but occurred in a much shorter time period.

Mean serum 25OHD concentrations were 17 ± 5 ng/ml and 15 ± 8 ng/ml at baseline and 3 months in the oral calcitriol group, versus 20 ± 10 ng/ml and 20 ± 9 ng/ml in the topical calcitriol group. Calcium levels (mean) were 9.4 mg/dl at baseline and 9.8 mg/dl at 3 months in the oral group (p=<0.05), versus 9.3 mg/dl and 9.2 mg/dl in the topical group. Calcitriol levels (mean) were 40 pg/ml (sd=11) and 47 pg/ml (sd=12) at baseline and 3 months in the oral group, versus 34 pg/ml (sd=11) and 35 pg/ml (sd=12) in the topical group.

Baseline and 3-month mean serum 25(OH)D$_3$, calcium and calcitriol concentrations for the 3 treatment groups are shown in Table 1.

Table 1. Baseline and 3-month mean serum 25O(H)D_3 (ng/ml), calcium (mg/dl) and calcitriol (pg/ml) concentrations in the 3 treatment groups.

	Treatment Group		
**Blood Test**	**Oral $1(OH)D_3$ Mean ± sd**	**Oral Calcitriol Mean ± sd**	**Topical Calcitriol Mean ± sd**
Baseline 25(OH)D_3	23 ± 12	17 ± 5	20 ± 10
3 Month 25(OH)D_3	27 ± 18	15 ± 8	20 ± 9
Baseline Calcium	9.2 ± 0.6*	9.4 ± 0.2*	9.3 ± 0.2
3 Month Calcium	9.6 ± 0.6*	9.8 ± 0.2*	9.2 ± 0.4
Baseline Calcitriol	41 ± 19*	40 ± 11	34 ± 11
3 Month Calcitriol	62 ± 32*	47 ± 12	35 ± 12
N	17	4	19

Note: No significant differences were observed in baseline versus 3-month 25(OH)D_3 levels in any group.*A significant difference between baseline and 3-month calcium levels was noted in the 1(OH)D_3 and oral calcitriol groups, and for calcitriol in the 1(OH)D_3 group, but all values were within the normal range. N = number of patients in each group.

The baseline and 3 month serum 25(OH)D_3 concentration ranges and the number of serum 25(OH)D_3 concentrations > 20, 50 or 100 ng/ml or < 20 ng/ml at each time point were not reported. However, the average serum 25(OH)D_3 concentration at baseline in the 40 patients in the 3 groups was 21 ± 11 ng/ml, indicating that a significant percentage had baseline serum 25(OH)D_3 concentrations > 20 ng/ml with active psoriasis, and improved significantly after treatment with oral or topical vitamin D.

There were no adverse reactions noted in any of the 3 groups of patients. "None of the patients in the three groups suffered from any topical or systemic complications or symptoms during these observation periods. Blood and urine analysis showed values within normal limits at all times. Hepatic and renal function, evaluated by measuring the serum levels of glutamic oxaloacetic transferase, glutamic pyruvic transferase, urea nitrogen and creatinine, were within normal ranges and did not change significantly during the observation periods."

Morimoto and colleagues published the results of 3 other clinical trials in the 1980s with similar results [15-16], and a review of their experience in 1989 [17]. In their review, they concluded: "These data suggest that exogenous active forms of vitamin D_3 are effective for the treatment of psoriasis, and that the endogenous 1,25-dihydroxyvitamin D level also may be involved in the development of this disease."

In the 1980s several important discoveries were made regarding vitamin D and the skin leading to the realization that the skin is both the site of production of vitamin D and a target organ for its actions:

a) Calcitriol could be synthesized in the skin

b) Vitamin D receptors are present in the skin

c) vitamin D inhibited the proliferation of cultured keratinocytes and induced them to terminally differentiate [18-20, 83-84, 90].

2.5. Oral and topical calcitriol in the 1980s and 1990s – Smith, Huckins, Perez and Holick [19, 21-22]

Reports published beginning in 1987 by Holick et al described the safe and effective use of oral calcitriol in treating psoriasis [18-23]. One report also examined the use of topical calcitriol and found it to be safe and effective as well [19]. Three of these reports will be reviewed in the next section.

2.6. Oral and topical calcitriol 12-month study, 1988 – Smith [19]

In 1988 [19] calcitriol was tested in three different ways:

a) On cultures of fibroblasts and keratinocytes from patients with psoriasis

b) Orally in 14 patients with moderate to severe psoriasis

c) Topically in 3 patients with psoriasis.

Baseline serum concentrations of 25(OH)D_3, calcitriol, calcium, phosphorous, total protein, albumin, blood urea nitrogen, creatinine, and a 24-hour urine collection for calcium and creatinine measurements were obtained. The daily oral doses of calcitriol used ranged from 0.5 µg to 2.0 µg. Post-treatment labs were not provided. In the oral calcitriol dosing study, all patients had moderate to extensive psoriasis, with large plaques on extensor surfaces. A total of 10 of the 14 patients (71%) showed at least a moderate response, while 77% had > 50% clearing of their skin. Complete clearing occurred in 3 patients (21%) "that was sustained with maintenance therapy."

"Thirteen of the 14 patients improved after < 2 months of oral calcitriol therapy and continued to improve for 6 to 8 months after initial improvement was observed." Four patients withdrew from the study, two for personal reasons, and two due to persistent hypercalciuria. Six of the patients chose to continue on with oral therapy and had been receiving treatment for one year without any complications. Four patients received little to no benefit." Two of the 10 patients showing the most improvement (>75% improved) had baseline serum 25(OH)D_3 concentrations of 40 ng/ml and 67 ng/ml. One (40 ng/ml) cleared completely within 2 to 3 months of therapy. The patient then stopped taking the calcitriol for 1 to 2 months, and the psoriasis reappeared. Treatment with calcitriol was resumed, and "the lesions cleared but at a slower rate than with the first course of treatment."

The 3 patients treated topically with calcitriol "showed a rapid response with complete clearing after 6 weeks of therapy," consistent with the results of the Morimoto study. No adverse reactions were noted in any of the patients treated with either oral or topical calcitriol. No side effects of hypercalcemia, calcium deposits, renal insufficiency or nephrolithiasis were observed in any of the patients in the study. In their evaluation of cultures of fibroblasts and keratinocytes from patients with psoriasis, the investigators were able to demonstrate the presence of vitamin D receptors in the cells, and a normal response to the anti-proliferative action of the hormone in fibroblasts from 3 of 5 patients and a partial resistance in the other two.

The range and distribution of pre-treatment serum 25(OH)D_3 concentrations in the combined oral and topical calcitriol treatment groups are shown in Table 2.

Table 2. Range and distribution of pre-treatment serum 25(OH)D_3 concentrations in the combined group of 15 psoriasis patients treated with oral calcitriol (n=13) and topical calcitriol (n=2).

**Pretreatment serum 25(OH)D_3 values**	
25(OH)D_3 Range ng/ml	8 to 67
# > 20 ng/ml	11
# > 50 ng/ml	4
# > 100ng/ml	0
# < 20 ng/ml	4
N	15

no data was available for 1 patient in each group. Post-treatment serum 25(OH)D_3 concentrations not provided. N = 15 patients: oral calcitriol (n=13) and topical calcitriol (n=2). # = total number of measurements above or below the indicated level pretreatment. N = total number of patient measurements.

In the combined oral and topical groups a total of 11 of 15 patients (73%) had pre-treatment serum 25(OH)D_3 concentration > 20 ng/ml, and 4 of 15 patients (27%) were > 50 ng/ml, with a peak serum 25(OH)D_3 concentration of 67 ng/ml. Four patients had baseline serum 25(OH)D_3 concentration < 20 ng/ml. In the oral dosing study, pre-treatment serum 25(OH)D_3 concentrations ranged from 8 ng/ml to 67 ng/ml. A total of 9 of 13 (69%) were > 20 ng/ml (one patient had no value). Post treatment serum 25(OH)D_3 concentrations were not reported but should have been unaffected by the calcitriol treatment. In the topical dosing study pre-treatment serum 25(OH)D_3 concentration were available for 2 of the 3 patients. Both were > 20 ng/ml (30 ng/ml and 67 ng/ml). Post treatment serum 25(OH)D_3 concentration were not reported.

The authors concluded their report stating: "Topical or oral use of 1,25-$(OH)_2D_3$ heralds a new mode of treatment that appears to be both safe and effective for the treatment of psoriasis." And as was shown by Morimoto, many patients with active psoriasis had baseline serum 25$(OH)D_3$ concentrations > 20 ng/ml and improved significantly after treatment with oral or topical vitamin D_3.

2.7. Oral calcitriol 6-month study in psoriatic arthritis, 1990 – Huckins [21]

In 1990 ten patients with active psoriatic arthritis were treated daily with oral calcitriol for 6 months in an open label trial. The goal of the study was to determine if the treatment would be beneficial for the arthritis, and if so, was there a correlation between the improvement in the skin lesions and the improvement in the arthritis. The dose of calcitriol was titrated from 0.5 μg/day to a maximum of 2 μg/day.

Statistically significant improvement was noted in both tender joint count and physician global assessment. Four patients had > 50% improvement in tender joint count, and 3 had > 25% improvement. Two patients were unable to receive therapeutic doses due to hypercalciuria. One patient was lost to follow-up.

In 6 patients who completed the 6-month study, the tender joint count decreased from a mean of 18 to 5, and the mean physician global assessment decreased from 8.4 to 1.8 (estimated from figure one in reference [58]). "It often took 2-3 months for improvement to occur, and improvement never occurred at a dosage < 1.5 ug/day." Five patients chose to stay on the treatment at the end of the study.

A number of labs were drawn at baseline and periodically throughout the study, but the values were not reported. However, the time to improvement was similar to the previous oral dosing studies, and a dose response was noted.

2.8. Oral calcitriol 3-year dose titration safety study, 1996 – Perez [22]

In 1996, a three-year follow-up study of 88 patients with plaque type psoriasis involving at least 15% of their body surface who were treated with oral calcitriol was published. The doses of calcitriol used ranged from 0.5 μg/day to 4.0 μg/day. The mean calcitriol dose was 2.1 μg/day at 24 months, and 2.4 μg/day at 36 months. A total of 88% of the patients noted some degree of improvement in their disease. Complete clearing occurred in 26.5%, moderate improvement occurred in 36.2%, and slight improvement occurred in 25.3%. A total of 12% of the patients had no change in their disease severity. The mean PASI score decreased from 18.4 at baseline to 9.7 at 6 months, 7.8 at 24 months and 7.0 at 36 months.

Multiple safety parameters were monitored over the 3-year period in an effort to perform a thorough evaluation of the potential toxicity of oral calcitriol. This included monitoring multiple blood tests, performing bone mineral density tests twice a year, checking for kidney stones with renal ultrasounds, measuring creatinine clearance, and 24-hour urinary excretion of calcium and creatinine.

Serum 25$(OH)D_3$ and calcium concentrations were provided at baseline (n=88), 6 months (n=88), 12 months (n=51), 24 months (n=26), and at 36 months (n=20, calcium only). There was no change in bone mineral density. Creatinine clearance decreased 13.4% from baseline in the first 6 months, then remained unchanged for the duration of the study. No cases of hypercalcemia, calcium deposition, nephrolithiasis or renal insufficiency or other adverse events were observed over the 3-year course of the study.

The mean serum 25$(OH)D_3$, calcium, PTH, calcitriol, creatinine, and 24-hour urine calcium concentrations at 0, 6, 12, 24 and 36 months in psoriasis patients treated with oral calcitriol are shown in table 3.

Table 3. Mean serum 25$(OH)D_3$, calcium, PTH, calcitriol, creatinine, and 24-hour urine calcium concentrations at 0, 6, 12, 24 and 36 months in 88 plaque psoriasis patients treated with oral calcitriol.

Blood Test	Baseline	6 month	12 month	24 month	36 month
25$(OH)D_3$ ng/ml	31.8 ± 18.4	33.8 ± 19.8	32.0 ± 20.8	37.2 ± 16.8	NR

Calcium mg/dl*	9.6 ± 0.5	9.9 ± 0.5	9.9 ± 0.5	9.8 ± 0.5	9.7 ± 0.4
PTH pg/ml	23.0 ± 14.8	NR	NR	12.4 ± 9.7	NR
Calcitriol pg/ml	40.4 ± 16.4	49.1 ±14.9	50.2 ± 23	44.1 ± 12.2	NR
Creatinine mg/dl	1.0 ± 0.2	1.1 ± 0.3	1.1 ± 0.3	1.2 ± 0.3	1.3 ± 0.3
24hr urine calcium*	163 ± 95	268 ± 117	291 ± 148	282 ± 135	274 ± 3
N	88	88	51	26	20

*The mean 24-hour urine calcium concentrations (mg/24hr) and calcium/creatinine ratios, and the serum calcium levels were significantly increased compared to baseline at 6, 12, 24 and 36 months. NR = not reported. The baseline and on treatment serum 25(OH)D_3 concentration ranges and the number of serum 25(OH)D_3 concentrations > 20, 50 or 100 ng/ml or < 20 ng/ml at each time point were not reported. N = total number of patient measurements at each time point.

The mean baseline serum 25(OH)D_3 concentration was 31.8 ± 14 ng/ml, indicating that many of the 88 patients had pre-treatment serum 25(OH)D_3 concentrations > 20 ng/ml (exact number not provided). The mean serum 25(OH)D_3 concentration was not affected by calcitriol supplementation and did not change significantly over time, as shown in the table. The mean serum calcium at baseline was 9.6 mg/dl and 9.7 mg/dl at 36 months.

As reported in the previous studies, many patients with active psoriasis had baseline pre-treatment serum 25(OH)D_3 concentrations > 20 ng/ml, but still improved significantly after treatment with oral vitamin D, in this case calcitriol. The fact that serum 25(OH)D_3 concentrations did not change was as expected, as calcitriol is the active hormone form of vitamin D and is not metabolized into serum 25(OH)D_3. At the end of the opening summary paragraph, the authors concluded "Oral calcitriol is effective and safe for the treatment of psoriasis."

These 5 studies [12,14,19, 21-22] detail the safe and effective use of oral vitamin D_2 (1936), oral 1(OH)D_3 (1986), oral calcitriol (1986,1988,1990,1996), and topical calcitriol (1986,1988) in treating psoriasis. There are very few reports published in the past 30 years describing the use of oral vitamin D_2 or oral vitamin D_3 (the precursors to serum 25(OH)D_3 and calcitriol) in treating psoriasis. Three relatively recent reports [25-26, 28] that do describe the use of oral vitamin D_3 (2012, 2013) and oral vitamin D_2 (2019) in successfully controlling plaque psoriasis will be reviewed next. These reports show similar clinical benefits and safety as the previous reports showed, even though much higher post-treatment serum 25(OH)D_3 concentrations were observed as would be expected, as both vitamin D_2 and vitamin D_3 are metabolized into 25(OH)D_3 prior to forming calcitriol.

2.9. Serum 25(OH)D_3 concentrations in 2 patients with plaque psoriasis after 5 months' oral vitamin D_3 in 2012 – McCullough [25]

In 2012, one of the authors (PM) presented a poster describing the results of using oral vitamin D_3 to successfully control chronic plaque psoriasis in 2 patients at the 15th Workshop on Vitamin D in Houston, Texas [25]. The patients included a 52-year-old white female and a 49-year-old white male. The study was conducted between December 2010 and May 2011. The patients were provided with over the counter 5000 IU vitamin D_3 gel caps and were instructed to take 40,000 IU/day for 2 weeks, and then reduce the dose to 10,000 IU/day.

Serum 25(OH)D_3, calcitriol, calcium and iPTH concentrations, and PASI scores were obtained at baseline and 5 months. Baseline PASI scores were 5.7 and 14.4. Baseline 25OHD_3 levels were 23 ng/ml and 29 ng/ml. After 5 months PASI scores improved to 0 and 2.4. Serum 25(OH)D_3 concentrations increased to 51 ng/ml and 73 ng/ml. Serum calcium concentrations remained normal at 9.4 mg/dl and 9.7 mg/dl, intact PTH concentrations were 27 pg/ml and 23 pg/ml, and calcitriol concentrations were 24 pg/ml and 56 pg/ml. No adverse reactions were noted, and both patients reported marked clinical improvement in their skin and in their quality of life. Both patients later experienced recurrence of the plaques within a month after stopping oral vitamin D_3 intake, and both were

able to achieve clear skin again after resuming the oral vitamin D_3. The recurrence of psoriasis with cessation of oral vitamin D intake and clearing again with resumption of oral vitamin D intake was also previously reported by Smith et al in 1988 [19].

Photos depicting the improvement in the skin over time in patient 2 after resuming oral vitamin D_3 at 40,000 IU/day are shown in Figure 1.

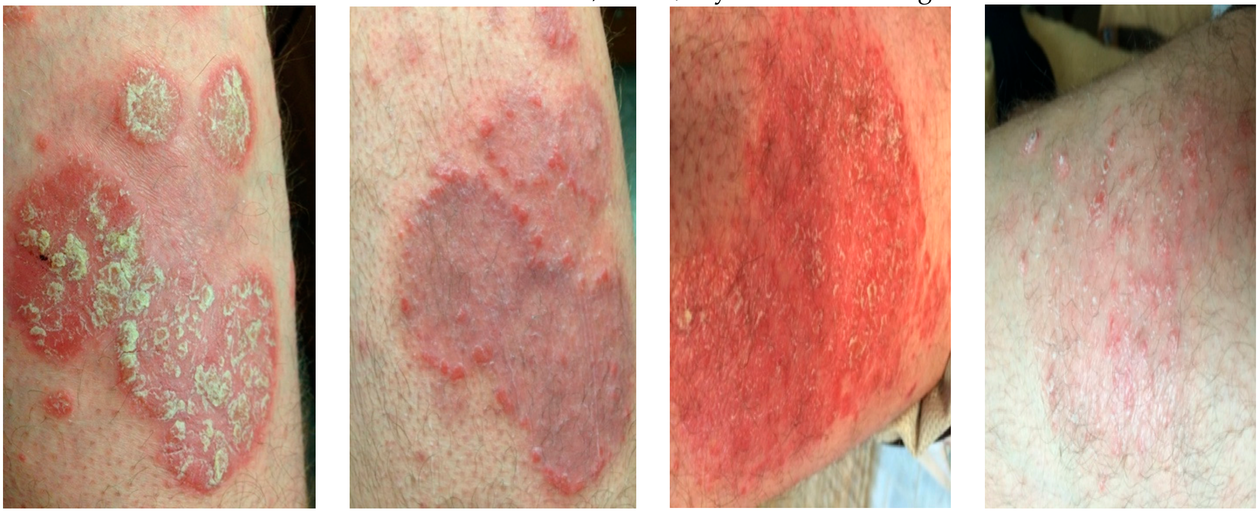

Day 1 Day 28 Day 72 Day 88

Figure 1. Improvement in psoriasis plaques after resuming 40,000 IU/day of vitamin D_3 in patient 2, whose disease recurred after stopping vitamin D_3. Nearly complete clearing of the plaques occurred by 88 days. The yearly cost of treatment with oral vitamin D_3 at a dose of 40,000 IU/day is around $104 per year, based on currently available over the counter pricing at $14/bottle for a USP verified bottle of 400 gel caps with 5000 IU/cap.

2.10. Serum 25(OH)D_3 concentrations after 6 months of taking 35,000 IU/day of oral vitamin D_3 in 25 patients with either plaque psoriasis or vitiligo, 2013 - Finamor [26]

In 2013, results from a 6-month follow-up study using 35,000 IU/day of oral vitamin D_3 to treat 9 patients with psoriasis and 16 patients with vitiligo were published [26]. The goal was to assess the efficacy and safety of prolonged high-dose vitamin D_3 treatment in patients with psoriasis and vitiligo. Psoriasis Area and Severity Index (PASI) scores were obtained at baseline and after treatment. The PASI score significantly improved in all nine patients with psoriasis. Fourteen of 16 patients with vitiligo had 25–75% repigmentation. A significant negative correlation was observed between the PASI scores and serum 25(OH)D_3 concentrations.

In the psoriasis group, mean serum 25(OH)D_3 concentrations increased from 14.9 ± 7.4 ng/ml at baseline to 106.3 ± 31.9 ng/ml at 6 months. Mean serum PTH concentrations decreased from 57.8 ± 16.7 pg/ml at baseline to 28.9 ± 8.2 pg/ml at 6 months.

In the vitiligo group, mean serum 25(OH)D_3 concentrations increased from 18.4 ± 8.9 ng/ml at baseline to 132.5 ± 37.0 ng/ml at 6 months. Mean serum PTH concentrations decreased from 55.3 ± 25.0 pg/ml at baseline to 25.4 ± 10.7 pg/ml at 6 months.

The changes in mean serum 25(OH)D_3 concentrations and PTH concentrations were significant in both groups. Baseline serum urea, creatinine and calcium (total and ionized) concentrations did not differ significantly from those obtained at 6 months. The mean baseline serum calcium concentration was 9.7 mg/dl, and 9.4 mg/dl after 6 months. Urinary calcium excretion increased but stayed within the normal range. The authors noted that "Laboratory or clinical signs of toxicity (hypercalcemia, hypercalciuria or kidney dysfunction) were not observed in any of the 25 participants, including a patient with vitiligo who reached a serum concentration of 25OHD_3 of 202ng/ml." Patients were instructed to minimize calcium intake. "Reduced intestinal calcium by dietary restriction of milk, dairy products and calcium-enriched foods (like oat, rice or soya "milk") has contributed to minimize the calciotropic effects of high daily doses of vitamin D_3 in the current study."

The distribution of serum 25(OH)D_3 concentrations, mean serum 25(OH)D_3, PTH, calcium, urea, and creatinine concentrations, and 24-hour urinary calcium values pre-and post-treatment are shown in table 4.

Table 4. Distribution and mean serum concentrations of 25(OH)D_3, PTH, calcium, urea and creatinine, and 24-hour urinary calcium in 9 patients with psoriasis and 16 patients with vitiligo pre-and 6 months' post-treatment with oral vitamin D_3 at 35,000 IU/day.

	Treatment Group			
**Blood Test**	**Psoriasis Baseline**	**Psoriasis 6 months**	**Vitiligo Baseline**	**Vitiligo 6 months**
25(OH)D_3 ng/ml	14.9 ± 7.4	106.3 ± 31.9	18.4 ± 8.9	132.5 ± 37
# > 20 ng/ml	NR	9	NR	16
# > 50 ng/ml	0	NR	0	NR
# > 100 ng/ml	0	>1	0	>1
PTH pg/ml	57.8 ± 16.7	28.9 ± 8.2	55.3 ± 25	25.4 ± 10.7
Calcium mg/dl	9.7 ± 0.7	9.4 ± 0.7	9.2 ± 0.3	9.2 ± 0.2
Urinary Calcium*	123.6 ± 60	226.8 ± 41.6*	158.3 ± 73.6	230.1 ± 81.4*
Urea mg/dl	35.8 ± 8.3	28.9 ± 9.8	35.5 ± 7.2	33.9 ± 9.9
Creatinine mg/dl	0.9 ± 0.2	0.8 ± 0.2	0.9 ± 0.2	0.9 ± 0.2
N	9	9	16	16

All patients presented with serum 25(OH)D_3 concentrations ≤ 30 ng/ml at baseline. *Urinary calcium increased significantly but remained in the normal range. PTH=parathyroid hormone. NR = not reported. The baseline and 6 month serum 25(OH)D_3 concentration ranges were not reported. # = total number of measurements above the indicated level pre and post treatment. N = total number of patients in each group at each time point.

The pre-treatment number of patients with baseline serum 25(OH)D_3 concentrations > 20 ng/ml was not indicated, but there were likely several among the 25 patients based on the standard deviation of the baseline mean serum 25(OH)D_3 concentrations. All 25 patients had serum 25(OH)D_3 concentrations < 30 ng/ml pre-treatment.

The post-treatment number of patients achieving serum 25(OH)D_3 concentrations > 100 ng/ml was not reported but is significant, based on the mean post treatment serum 25(OH)D_3 concentrations of 106.3 ± 31.9 ng/ml and 132.5 ± 37 ng/ml. The highest serum 25(OH)D_3 concentration observed post-treatment was 202 ng/ml.

"Dietary calcium limited by avoiding dairy products and calcium-enriched foods – like oat, rice or soya "milk" and minimum hydration (2.5 L daily) ensures safety." The authors' main conclusion was "In summary, the present study suggests that, at least for patients with autoimmune disorders like vitiligo and psoriasis, a daily dose of 35,000 IU of vitamin D_3 is a safe and effective therapeutic approach for reducing disease activity."

The yearly cost of treatment with oral vitamin D_3 at a dose of 35,000 IU/day is around $98 per year, based on currently available over the counter pricing at $14/bottle for a USP verified bottle of 400 gel caps with 5000 IU/cap.

2.11. Serum 25(OH)D_2 concentrations in a patient with plaque psoriasis after 42 months of taking 50,000 IU/day of oral vitamin D_2, 2019 - McCullough [28]

In 2019, a paper describing results from supplementing long-term hospitalized patients with 5000 IU/day to 50,000 IU/day of vitamin D_3 for over 7 years was published by one of the authors [28]. This author (PM) made it a standard of care beginning in April 2009 to offer all long-term hospitalized patients under his care at the Drake Center for Post-Acute Care supplementation with oral vitamin D_3 in doses of either 5000 IU/day or 10,000 IU/day, and this was continued after moving to Summit Behavioral Healthcare (SBH) in July 2011. This was done for several reasons:

a) patients receive very little sunshine in the hospital

b) there is very little vitamin D in the food they eat

c) serum 25-hydroxyvitamin D (25(OH)D) production in the skin from UVB phototherapy was first estimated in the 1970s to range from 10,000 to 25,000 IU/day [39, 53, 56, 110-113]

d) vitamin D, sunshine and UVB phototherapy were shown to be effective treatments for several diseases in the 1930s and 1940s, and again beginning in the 1980s as discussed earlier [12-39, 45-54]. In addition, several patients received daily doses of vitamin D_2 or vitamin D_3 ranging from 20,000 to 50,000 IU/day based on specific disease concerns.

There have been over 6000 admissions to SBH since 2011. A recent sampling of patients not on vitamin D_3 (n=777; combination of new admissions and long-term patients who declined supplementation) showed a mean serum 25(OH)D_3 concentration of 27.1 ng/ml (range 4.9 to 74.8 ng/ml). Patients on vitamin D_3 long enough to develop serum 25(OH)D_3 concentrations > 74.4 ng/ml (n=418) had a mean serum 25(OH)D_3 concentration of 118.9 ng/ml (range 74.4 to 384.8 ng/ml). The highest serum 25(OH)D_3 concentrations observed on 10,000 IU/day was 202 ng/ml.

The mean and range of serum calcium concentrations were almost identical in the two groups, despite the wide disparity in serum 25(OH)D_3 concentrations. The average serum calcium concentrations were 9.5 mg/dl (no D_3) vs. 9.6 mg/dl (D_3), with ranges of 8.4 mg/dl to 10.7 mg/dl (no D_3) vs. 8.6 mg/dl to 10.7 mg/dl (D_3), after excluding patients with other causes of hypercalcemia. The average intact parathyroid hormone concentrations were 24.2 pg/ml (D_3) vs. 30.2 pg/ml (no D_3).

There were no adverse events observed in any patients taking 5000 to 10,000 IU/day for several years, in spite of serum 25(OH)D_3 concentrations reaching as high as 202 ng/ml. In addition, several patients, as well one of the authors, having taken daily oral doses of vitamin D ranging from 20,000 to 60,000 IU/day for 2 to 6 years, achieved serum 25(OH)D_3 concentrations as high as 384ng/ml without any complications [28,114].

In our 2019 report we included a case report of a patient admitted with poorly controlled plaque psoriasis whose skin improved dramatically within a few months of starting 50,000 IU/day of oral vitamin D_2 and has remained clear for many months. He is no longer using the topical steroids or medicated shampoos which he was taking at the time of admission and is no longer being seen by specialists in the dermatology clinic at the local medical school. After his skin cleared, the patient chose to leave the dose of vitamin D_2 at 50,000 IU/day, which is provided in a single capsule, and continued on this treatment. His serum calcium and iPTH concentrations have been checked numerous times and have remained normal. No adverse events related to vitamin D supplementation have been observed. His quality of life has improved significantly.

His serum 25(OH)D_2, iPTH and calcium blood concentrations and skin condition are shown in Table 5.

Table 5. Changes in serum 25(OH)D_2, iPTH and calcium concentrations over time in a patient with psoriasis completely cleared on 50,000 IU/day of vitamin D_2 for > 42 months.

Date	25(OH)D_2	iPTH	Calcium	Psoriasis status
2/27/2016	70.5	40	9.5	severe
5/27/2016			9.5	Marked improvement
10/20/2016			9.6	mild
12/3/2016			9.6	skin clear
12/15/2016			9.7	skin clear
1/12/2017			9.4	skin clear
1/28/2017	262		9.5	skin clear
3/6/2017	297.6		9.6	skin clear
4/13/2017	290.8	38	9.6	Derm clinic discontinued
6/10/2017	296.4		9.8	skin clear
9/6/2017			9.4	skin clear
12/6/2017	249.6	29	9.5	skin clear
3/2/2018	308.4	32	9.6	skin clear
6/6/18			9.9	skin clear
7/17/18			9.6	skin clear
9/5/18	290	33	9.7	skin clear

3/5/19	225.2	32	9.4	skin clear
6/25/19			9.3	skin clear

Vitamin D_2 50,000 IU/day was started on 2/25/16, two days before his admission blood work was drawn. Eight serum 25(OH)D_2 concentrations have ranged from 225 ng/ml to 308 ng/ml, 22 serum calcium levels ranged from 9.2 mg/dl to 9.9 mg/dl, and six iPTH levels ranged from 29 pg/ml to 40 pg/ml. A 24-hour urine for calcium and creatinine was collected on 6/9/17. The total 24 hr. calcium excretion was 316.8 mg (normal = 100 to 300 mg/24hr), and the urinary ca/cr ratio was 207 (normal = 0 to 260).

The yearly cost for the vitamin D_2 used was $36.50, as we are able to obtain 50,000 IU capsules of vitamin D_2, with 100 capsules/bottle for $10 a bottle.

3. Changes in serum 25(OH)D_3 concentrations in psoriasis patients treated with UVB phototherapy and sunshine – 1996, 2009, and 2010

The use of phototherapy to treat disease dates back to the 1890s when Finsen developed a method to cure TB with refracted light rays from an electric arc lamp [28,39-44,48-50,52,54]. Several recent reviews give an excellent overview of the evolution of the use of phototherapy for treating human disease, including psoriasis [40-44]. The first documented use of UVB phototherapy in treating psoriasis dates back to Gockerman in the 1920s [41-43]. UVB phototherapy is now a well-established, relatively safe and cost-effective option for treating psoriasis [40-44, 61-64, 115-131].

In this section, we will review four UVB phototherapy psoriasis treatment studies that provided baseline and post-treatment serum 25(OH)D_3 concentrations [61-64]. Significant increases in 25(OH)D_3 from baseline were noted in the UVB phototherapy studies, with several patients obtaining serum 25(OH)D_3 concentrations > 100 ng/ml without any adverse effects while observing significant improvement in their skin. As noted in the oral vitamin D studies, baseline serum 25(OH)D_3 concentrations > 20 ng/ml were also commonly observed in these reports and increased after treatment. One study also included a group of patients treated with sunshine, in which the observed changes in serum 25(OH)D_3 concentrations were significantly lower than those after treatment with UVB therapy [64],

3.1. Serum 25(OH)D_3 concentrations in psoriasis patients after 8 weeks of UVB phototherapy, 1996 - Prystowsky [61]

In 1996 changes in serum 25(OH)D_3 concentrations were assessed in 15 patients with plaque-type psoriasis treated with UVB phototherapy [61]. Seven of these patients were treated with oral calcitriol (0.5 to 2 ug/day), and eight with placebo. Nineteen patients were initially enrolled, but four did not complete the study because of protocol violations. Serum concentrations of 25(OH)D_3 and calcitriol were measured before, during and after treatment in 13 patients. Serum chemistry and hematology laboratory evaluations were also done.

All patients treated with phototherapy showed significant increases in their serum 25(OH)D_3 concentrations. No patient incurred a serious adverse event attributable to calcitriol or phototherapy that necessitated removal from the study. Significant improvement was noted in disease severity in all patients in both groups, with no significant difference between groups.

In the placebo group mean serum 25(OH)D_3 concentrations increased from 37.9 ng/ml at baseline to 96.1 ng/ml after UVB phototherapy. In the calcitriol group mean serum 25(OH)D_3 concentrations increased from 27.3 ng/ml at baseline to 67.1 ng/ml after UVB phototherapy. The authors stated there was no significant difference in the mean increments between the 2 groups.

Serum calcitriol concentrations were unchanged in the placebo treated group and increased in the calcitriol treated group. In the placebo group, the mean serum calcitriol concentration was 38.3 pg/ml (sd=9.9) at baseline and 35.2 pg/ml (sd=19.7) at the end of phototherapy. In the calcitriol group, the mean serum calcitriol concentration was 37.9 pg/ml (sd=8) at baseline and increased to 60.1 pg/ml (sd=24.9) after phototherapy.

The mean serum calcium concentration post-treatment was 9.6 mg/dl in the placebo group, and 9.7 mg/dl in the calcitriol group. Baseline serum calcium concentrations were not provided.

Hypercalcemia was observed in 2 patients in the calcitriol group, but neither had hypercalciuria. The hypercalcemia resolved with reduction in their intake of calcitriol. Three patients in each group developed hypercalciuria (values not provided). There were no adverse events related to hypercalcemia or hypercalciuria. The range and distribution of serum 25(OH)D_3 concentrations and mean 25(OH)D_3, calcium and calcitriol concentrations pre-and post UVB treatment ± oral calcitriol are shown in table 6.

Table 6. Mean, range and distribution of serum 25(OH)D_3 concentrations, and mean serum calcium and calcitriol concentrations before and after UVB phototherapy in 13 patients with plaque psoriasis also treated with placebo (n=7) or calcitriol (n=6).

	Placebo Group		Calcitriol Group	
**Test**	**Pre-UVB**	**Post-UVB**	**Pre-UVB**	**Post-UVB**
25(OH)D_3 ng/ml	37.9	96.1	27.3	67.1
Range ng/ml	20 to 80	45 to 159	15 to 40	45 to 123
# > 20 ng/ml	7	7	4	6
# > 50 ng/ml	2	6	0	4
# > 80 ng/ml	1	4	0	1
# > 100 ng/ml	0	2	0	1
Calcium mg/dl	NR	9.6	NR	9.7
Calcitriol pg/ml	38.8	35.2	37.9	67.1
N	7	7	6	6

The distribution and range of serum 25(OH)D_3 concentrations are estimated from Figure 2 in reference [61]. N= total number of patients in each group. # = total number of measurements above the indicated level pre and post treatment. NR = not reported.

Pre-treatment 11 of 13 patients (85%) appeared to have serum 25(OH)D_3 concentrations > 20 ng/ml. Two of 13 patients (15%) had a baseline serum 25(OH)D_3 concentration > 50 ng/ml, one of whom appeared to be > 80 ng/ml. Post-treatment 13 of 13 patients (100%) were > 20 ng/ml, 10 of 13 (77%) had a serum 25(OH)D_3 concentrations > 50 ng/ml, and 3 patients (23%) had serum 25(OH)D_3 concentrations > 100 ng/ml, two in the placebo group and one in the calcitriol group. Their serum 25(OH)D_3 concentrations ranged from 123 ng/ml to 159 ng/ml.

The authors noted that "because phototherapy for psoriatic plaques produces changes in keratinocytes similar to those described for 1,25-(OH)2D3 (i.e. slowed proliferation and enhanced differentiation), this raises the possibility that one of the mechanisms of action of UVB may be through enhanced vitamin D metabolism."

3.2. Serum 25(OH)D_3 concentrations in psoriasis patients after 1-4 months of NB-UVB phototherapy, 2010 - Ryan [62]

In a 2010 report, serum 25(OH)D_3, ionized calcium, intact parathyroid hormone (iPTH) and alkaline phosphatase concentrations were assessed in 30 patients with plaque psoriasis before and after treatment with narrowband (NB) UVB phototherapy [62]. Comparison was made to a matched untreated control group of 30 patients with plaque psoriasis.

Patients in the treatment group received NB-UVB phototherapy 2 to 3 times a week. Treatment continued until essentially complete clearing of the psoriasis occurred, which took between 25 to 118 days (median 51 days). Baseline PASI scores ranged from 4.2 to 16.1 (median 7.1) in the treatment group, and from 0 to 12.5 (median 3.6) in the control group.

In the NB-UVB group baseline serum 25(OH)D_3 concentrations ranged from 9 ng/ml to 46 ng/ml (median 23 ng/ml). Post-NB-UVB phototherapy, after complete skin clearing, the range of serum 25(OH)D_3 concentrations increased to 32 to 112 ng/ml (median 51 ng/ml).

In the control group, baseline serum 25(OH)D_3 concentrations ranged from 7 ng/ml to 42 ng/ml (median 12 ng/ml) and ranged from 7 ng/ml to 33 ng/ml (median 13 ng/ml) when reassessed at the same time as their matched NB-UVB treated partner ended the study. There was no change in the skin condition or serum 25(OH)D_3 concentrations in the control group. Serum ionized calcium concentrations were normal and remained unchanged in both groups throughout the study (values not provided). None of the patients developed hypercalcemia or any other adverse events. The change in serum 25(OH)D_3 concentrations correlated with the number of exposures to NB-UVB and cumulative UVB dose, but not with treatment response.

The distribution and median serum 25(OH)D_3 concentrations pre-and post UVB treatment are shown in table 7.

Table 7. Range, median and distribution of serum 25(OH)D_3 concentrations pre-and post NB-UVB treatment in 29 psoriasis patients and 29 untreated controls.

Measurement	NB-UVB Group N=29	Control Group N=29
Pre-UVB 25(OH)D_3 range	9 to 46 ng/ml	7 to 42 ng/ml
Median 25(OH)D_3 level	23 ng/ml	12 ng/ml
# > 20 ng/ml	19	NR
# > 40 ng/ml	NR	NR
# < 20 ng/ml	10	NR
Post-UVB 25(OH)D_3 range	32 to 112 ng/ml	7 to 33 ng/ml
Median 25(OH)D_3 level	51 ng/ml	13 ng/ml
# > 20 ng/ml	29	7
# > 40 ng/ml	24	0
# > 51 ng/ml	15	0
# > 72 ng/ml	6	0
# > 100 ng/ml	1+	0
# < 20 ng/ml	0	NR

N=29 patients in each group. NR = not reported. Patients were treated 2 to 3 times a week until essentially complete clearing of the psoriasis occurred, which took between 25 to 118 days. The control group did not receive NB-UVB phototherapy. # = total number of measurements above or below the indicated level pre and post treatment.

Pre-treatment 19 of 29 patients (64%) in the NB-UVB group had serum 25(OH)D_3 concentrations > 20 ng/ml. Post-treatment all 29 patients (100%) had serum 25(OH)D_3 concentrations > 20 ng/ml, 24 patients (83%) were > 40 ng/ml, 15 (50%) were > 51 ng/ml, and 6 (20%) were >72 ng/ml. The number achieving serum 25(OH)D_3 concentrations > 100 ng/ml post-treatment was not reported, but occurred in at least 1 patient, with a peak value of 112 ng/ml.

3.3. Serum 25(OH)D_3 concentrations in psoriasis patients after 8-12 weeks of NB-UVB and BB-UVB phototherapy, 2009 - Osmancevic [63]

In a 2009 report, serum 25(OH)D_3, calcitriol, iPTH, calcium and creatinine concentrations were measured in 68 patients with plaque psoriasis before and after treatment with either broadband UVB (BB-UVB, n=26) or NB-UVB (n=42) phototherapy [63]. All patients were treated with whole body exposure for 8 to 12 weeks, with the doses of UVB adjusted based on the skin phenotype and the erythemal response noted during treatment.

The purpose of the study was to determine if there was a difference in vitamin D production with NB-UVB versus BB-UVB phototherapy. The use of oral or topical vitamin D, vitamin D analogues, or any biologics was prohibited. Patients were treated either in the spring (n=39) or in the winter (n=29).

There was no significant difference in the total number of treatments needed, but the treatment time was four times longer in the NB-UVB group compared to the BB-UVB group. Psoriasis plaques improved in all patients in both groups. Mean PASI scores decreased from 8.8 to 2.3 in the NB-UVB group, and from 9.5 to 3.1 in the BB-UVB group. The improvement in psoriasis was found to correlate positively with the increase in

25OHD levels (p=0.047). It was not stated if any patients achieved complete clearing of their skin lesions.

Serum 25(OH)D_3 concentrations increased in both groups, with a more pronounced increase noted in the BB-UVB versus NB-UVB group. Serum concentrations of calcium, creatinine, and 1,25-dihydroxyvitamin D3 were unchanged, while iPTH concentrations decreased in the BB group. In the BB-UVB group, the baseline mean serum 25(OH)D_3 concentration was 37.9 ± 16.9 ng/ml and increased to 69.4 ± 19.7 ng/ml after treatment. In the NB-UVB group, the baseline mean serum 25(OH)D_3 concentration was 34.8 ± 11.9 ng/ml and increased to 55.3 ± 17.6 ng/ml after phototherapy.

A line plot of individual serum 25(OH)D_3 concentrations before and after treatment for the two groups showed that at least 3 patients in the BB-UVB group had serum 25(OH)D_3 concentrations > 100 ng/ml post-treatment, but the actual values were not indicated. The distribution and mean serum 25(OH)D_3 concentrations pre-and post UVB treatment are shown in table 8.

Table 8. Mean, range and distribution of serum 25(OH)D_3 concentrations before and after BB-UVB (n=26) and NB-UVB (n=42) phototherapy in 68 patients with psoriasis.

Treatment	Pre-UVB 25(OH)D_3 ng/ml Mean ± sd	Post-UVB 25(OH)D_3 ng/ml Mean ± sd
BB-UVB (n=26)	37.9 ± 16.9	69.4 ± 19.7
Range ng/ml	17 to 82	45 to 118
NB-UVB (n=42)	34.8 ± 11.9	55.3 ± 17.6
Range ng/ml	15 to 73	28 to 98
# > 20 ng/ml	65	68
# > 50 ng/ml	5	48
# > 80 ng/ml	1	13
# > 100 ng/ml	0	3
# < 20 ng/ml	3	0

Note: the range and distribution of serum 25(OH)D_3 concentrations are estimated from Figure 2 in reference [63]. # > ng/ml is the sum of patients from both groups. All patients were treated with whole body exposure to UVB for 8 to 12 weeks. The difference in serum 25(OH)D_3 concentrations after treatment was significant between the lamps (p-value=0.008). # = total number of measurements above or below the indicated level pre and post treatment.

Pre-treatment, 65 of the 68 patients (95.6%) had serum 25(OH)D_3 concentrations > 20 ng/ml, while 5 of 68 (7.4%) were > 50 ng/ml, and one was > 80 ng/ml. Post-treatment a total of 48 of 68 patients (70.6%) had serum 25(OH)D_3 concentrations > 50 ng/ml; 13 of 68 (19.1%) were > 80 ng/ml; and 3 of 68 (4.4%) were > 100 ng/ml (values not indicated). No adverse events related to the treatment were noted in any patient.

3.4. Serum 25(OH)D_3 concentrations in psoriasis patients after 15 days of sunshine or 8-12 weeks of NB-UVB or BB-UVB phototherapy, 2010 - Osmancevic [64]

In a 2010 report, serum 25(OH)D_3, calcitriol, PTH, calcium and creatinine concentrations in psoriasis patients were measured before and after treatment with sunshine, NB-UVB and BB-UVB phototherapy [64]. This report was a discussion of data aggregated from 3 studies, including data in the previously discussed report [63]. The two additional studies included a group of 24 post-menopausal women with psoriasis who were treated with whole body BB-UVB phototherapy 2 to 3 times a week for 8 to 12 weeks, and a group 20 psoriasis patients who were treated with whole body heliotherapy (sunshine) daily for 2 weeks. The authors stated that they had 2 main aims:

a.) To increase the knowledge about the effects of phototherapy on vitamin D production during the treatment of psoriasis,

b.) To see if there were differences between the effect of BB-UVB, NB-UVB and heliotherapy on vitamin D synthesis in psoriasis patients.

A similar efficacy was reported with each treatment. An improvement in the PASI score of about 75% was observed in each group. However, the group treated with sunshine required only two weeks to achieve the same clinical improvement as seen after 2 to 3 months of UVB phototherapy.

Serum 25(OH)D_3 concentrations increased in each group. No changes in serum calcium concentrations were noted after any of the phototherapy regimens used. In the BB-UVB group of post-menopausal women, the mean baseline serum 25(OH)D_3 concentration was 36.8 ± 17 ng/ml and increased to 59.6 ± 18.7 ng/ml after phototherapy. In the sunshine group, the mean baseline serum 25(OH)D_3 concentration was 22.9 ± 6.0 ng/ml , which increased to 41.8 ± 6.3 ng/ml after daily sunshine exposure for 15 consecutive days. The range of serum 25(OH)D_3 concentrations after treatment with sunshine was much lower than in the UVB groups. This may be due to the shorter duration of treatment, as well as the fact that the patients used sunscreen on areas of their body susceptible to sunburn.

The mean, range and distribution of serum 25(OH)D_3 concentrations pre-and post BB-UVB and sunshine treatment in the postmenopausal and sunshine groups are shown in table 9.

Table 9. Mean, range and distribution of serum 25(OH)D_3 concentrations pre-and post 8-12 weeks of BB-UVB phototherapy (n=24 postmenopausal women) or 15 days of whole-body sunshine (n=20) exposure in 44 patients with plaque psoriasis.

Treatment	Pre-treatment	Post-treatment
BB-UVB	BB-UVB	BB-UVB
25(OH)D_3 ng/ml	36.8 ± 17	59.6 ± 18.7
Range ng/ml	18 to 88	25 to 90
# > 20 ng/ml	22	24
# > 50 ng/ml	3	17
# > 80 ng/ml	0	5
# > 100 ng/ml	0	0
# < 20 ng/ml	2	0
N	24	24
Sunshine	Sunshine	Sunshine
25(OH)D_3 ng/ml	22.9 ± 6	41.8 ± 6.3
Range ng/ml	18 to 42	30 to 60
# > 20 ng/ml	17	20
# > 50 ng/ml	0	4
# > 80 ng/ml	0	0
# > 100 ng/ml	0	0
# < 20 ng/ml	3	0
N	20	20

The range and distribution of serum 25(OH)D_3 concentrations is estimated from Figure 2 in reference [64]. BB-UVB group was treated with whole body phototherapy 2 to 3 times a week for 8 to 12 weeks. Sunshine group was treated with whole body heliotherapy (sunshine) daily for 2 weeks. N= number of patients in each group. # = total number of measurements above the indicated level pre and post treatment. N = total number of patients in each group at each time point.

In the BB-UVB group, pre-treatment serum 25(OH)D_3 concentrations were > 20 ng/ml in 22 of 24 patients (91.7%) and were > 50 ng/ml in 3 of 24 patients (12.5%). Post-treatment, a total of 17 BB-UVB patients (70.8%) had serum 25(OH)D_3 concentrations > 50 ng/ml, 5 (20.8%) were > 80 ng/ml, and none were above 100ng/ml. In the sunshine group, pre-treatment serum 25(OH)D_3 concentrations were > 20ng/ml in 17 of the 20 patients (85%), and none were > 50 ng/ml. Post-treatment, a total of four patients (20%) had serum 25(OH)D_3 concentrations > 50ng/ml, and none were > 100ng/ml. All patients in the sunshine treated group had serum 25(OH)D_3 concentrations greater than 30ng/ml after two weeks of daily sun exposure, with significant improvement in their clinical condition.
